# Targeted degradation of MDM2 overcomes feedback regulation of p53 signaling in Merkel cell carcinoma models

**DOI:** 10.1172/JCI199049

**Published:** 2026-07-01

**Authors:** Varsha Ananthapadmanabhan, Simone Bruno, Leonard Vonk, Yu-Chen Cheng, Abeba Teshager, Benjamin K. Eschle, Charles L. Howarth, Joana S. Rodrigues, Julia Schnabel, Ananya Kodali, Prafulla C. Gokhale, Rujuta Kshirsagar, Susanne B. Breitkopf, Kirti Sharma, Joao A. Paulo, Yvonne Li, Andrew D. Cherniack, Franziska Michor, Yogesh Chutake, Joyoti Dey, James A. DeCaprio

**Affiliations:** 1 Department of Medical Oncology, Dana-Farber Cancer Institute, Boston, Massachusetts, USA.; 2 Department of Medicine, Brigham and Women’s Hospital and Harvard Medical School, Boston, Massachusetts, USA.; 3 Department of Data Science, Dana-Farber Cancer Institute, Boston, Massachusetts, USA.; 4 Department of Biostatistics, Harvard T.H. Chan School of Public Health, Boston, Massachusetts, USA.; 5 Department of Stem Cell and Regenerative Biology, Harvard University, Cambridge, Massachusetts, USA.; 6 The Broad Institute of MIT and Harvard, Cambridge, Massachusetts, USA.; 7 Experimental Therapeutics Core, Dana-Farber Cancer Institute, Boston, Massachusetts, USA.; 8 Kymera Therapeutics, Inc., 500 North Beacon Street, Watertown, Massachusetts, USA.; 9 Program in Virology, Graduate School of Arts and Sciences, Harvard University, Cambridge, Massachusetts, USA.; 10 Department of Cell Biology, Harvard Medical School, Boston, Massachusetts, USA.; 11 Center for Cancer Evolution, Dana-Farber Cancer Institute, Boston, Massachusetts, USA.; 12 The Ludwig Center at Harvard, Boston, Massachusetts, USA.

**Keywords:** Cell biology, Oncology, Apoptosis, Therapeutics, p53

## Abstract

*MDM2* is transcriptionally activated by the ST-MYCL-Tip60 complex in virus-positive Merkel cell carcinoma (MCC). MDM2 suppresses p53 and is a rational therapeutic target. MDM2 inhibitors face an intrinsic limitation: p53 activation induces MDM2 transcription, creating a feedback loop that blunts inhibitor efficacy. We demonstrate that MDM2 degraders KTX-049 and KT-253 overcome this limitation by collapsing the p53/MDM2 negative feedback loop. KTX-049 was >100-fold more potent than the MDM2 inhibitor DS-3032 across WT p53 MCC cell lines, and this superior potency was quantitatively supported by mechanistic mathematical modeling. In vivo, KT-253 produced deep and durable tumor regressions, including complete responses in patient-derived xenograft models. Acquired resistance was strongly associated with acquisition of *TP53* mutations, confirming on-target pathway pressure. These findings establish feedback architecture as a critical determinant of therapeutic response and position MDM2 degradation as a qualitatively distinct strategy that produces more durable pathway engagement than MDM2 inhibition, providing a preclinical rationale for prioritizing MDM2 degraders in WT *TP53* MCC.

## Abstract 2

Targeted degradation of MDM2 reactivates p53 with 100-fold greater potency than inhibition and achieves durable tumor regression in preclinical Merkel cell carcinoma models.

## Graphical abstract

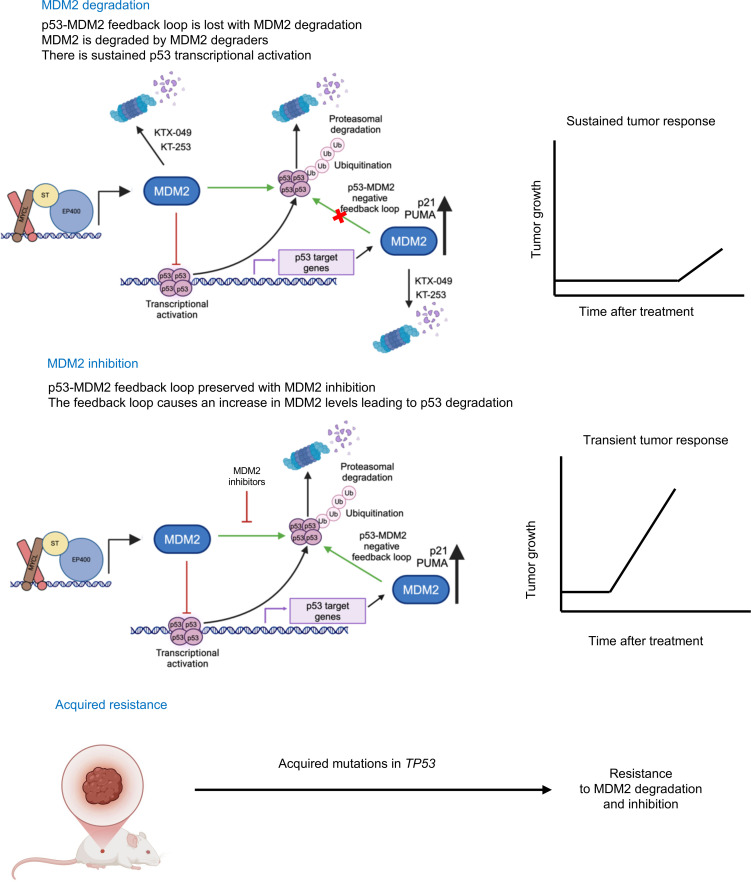

## Introduction

Merkel cell carcinoma (MCC) is an aggressive neuroendocrine carcinoma of the skin characterized by rapid growth and high propensity for metastasis (1–5). Despite its rarity, the incidence of MCC has been steadily increasing, posing marked challenges to its effective management and treatment. The 5-year disease-associated mortality rate remains alarmingly high, at approximately 40%, underscoring the urgent need for other therapeutic strategies (6–11). Current treatment options, including surgery, radiation, and chemotherapy, often yield limited success, particularly in advanced stages of the disease. Immune checkpoint inhibitors, such as PD-1 and PD-L1 antibodies, have shown promise, but their efficacy is often short-lived, and resistance frequently develops (2, 3, 6, 11, 12). Thus, there is a critical need to explore alternative therapeutic options that can offer more durable responses.

A substantial proportion of MCC cases is associated with clonal integration of Merkel cell polyomavirus, which plays a crucial role in the oncogenic process (2, 13). Virus-positive MCC (MCCP) tumors typically exhibit a low mutational burden and retain the WT *TP53* and *RB1* tumor suppressor genes. In contrast, virus-negative MCC (MCCN) tumors often display a high mutational burden with frequent alterations in these genes (2, 4, 14–16). The presence of Merkel cell polyomavirus in MCCP tumors leads to the expression of viral oncoproteins, small T antigen (ST), and a truncated form of large T antigen, which contribute to tumorigenesis by inactivating key tumor suppressor pathways (2, 4, 14–19). Large T antigen binds to and inactivates the retinoblastoma protein RB (2, 4, 17–19). ST forms a specific complex with MYCL and the Tip60 complex to transactivate a large number of genes that contribute to the oncogenesis of MCCP (2, 20).

One of the critical downstream targets of the ST-MYCL-Tip60 complex in MCCP is *MDM2*, an E3 ubiquitin ligase that negatively regulates p53. MDM2 binds to p53 and promotes its degradation, thereby inhibiting its tumor-suppressive function (2, 20–22). MDM2 inhibitors disrupt the interaction between MDM2 and p53, leading to stabilization of p53 (23–25). However, increased p53 activity leads to elevated MDM2 levels, as a result of a negative feedback loop that can limit the efficacy of traditional MDM2 inhibitors by counteracting the intended therapeutic effect (26). Therefore, targeting this feedback loop through different approaches, such as MDM2 degraders, is a promising strategy to enhance p53 activity and improve therapeutic outcomes (27–30).

Here, we investigated the MDM2 degraders KTX-049 and KT-253 in MCC models. We tested whether MDM2 degradation could overcome feedback-mediated limitations of MDM2 inhibition by eliminating MDM2 protein and producing more durable p53 pathway activation. Because MDM2 is embedded within a tightly regulated negative feedback loop, inhibition of its activity may be limited by compensatory MDM2 induction. We therefore hypothesized that targeted degradation of MDM2 could overcome a fundamental liability of inhibition in this feedback-regulated circuit.

## Results

### KTX-049 is 100-fold more potent than DS-3032 in WT p53 MCC.

**Figure 1:**
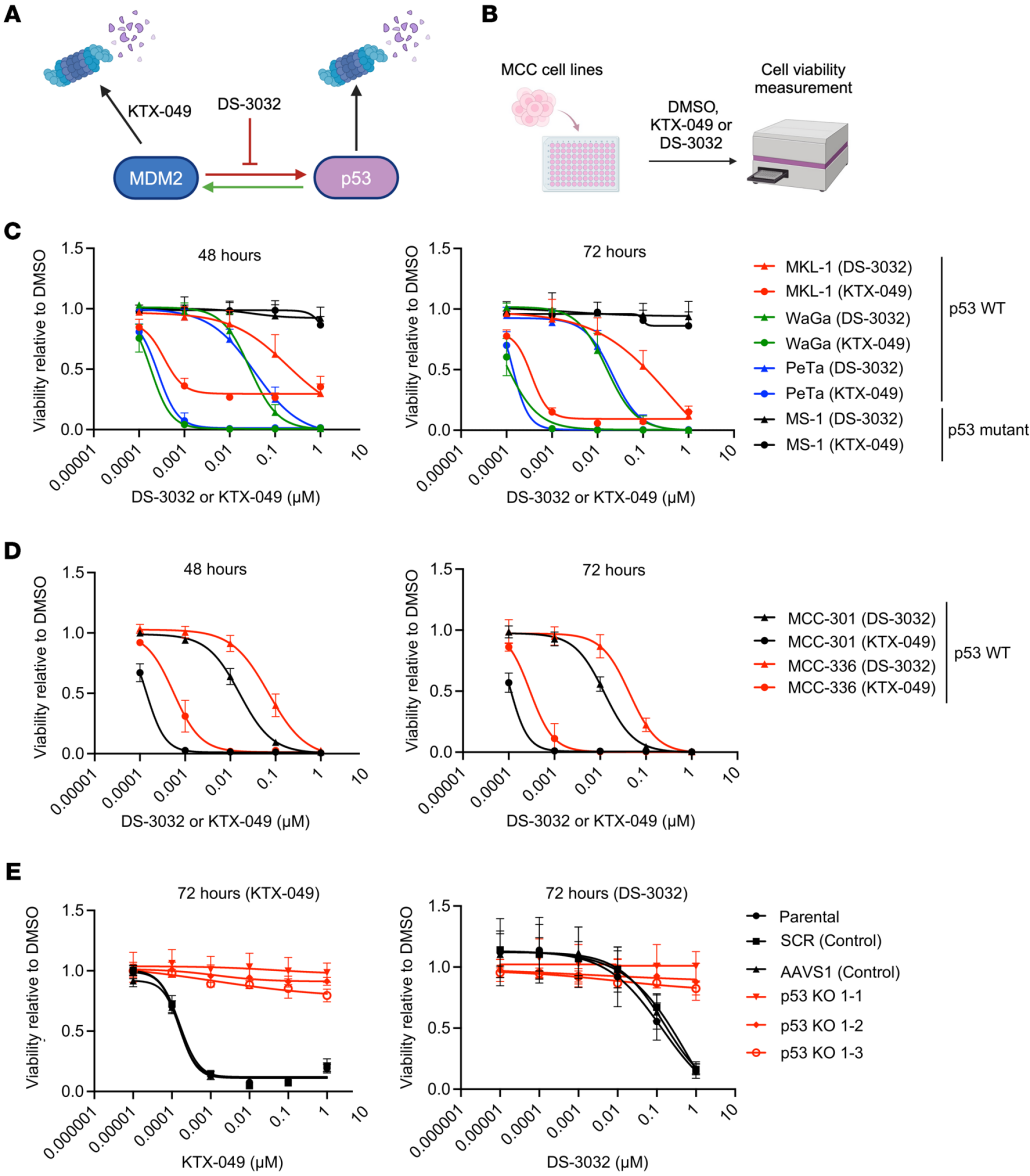
MCCP cell lines with WT p53 are highly sensitive to KTX-049. (**A**) Scheme indicates MDM2 degrader KTX-049 acts on MDM2 and the MDM2 inhibitor inhibits the p53/MDM2 interaction. (**B**) Scheme showing cell lines were treated with DMSO, KTX-049, or DS-3032 followed by viability measurement, as shown in **C**–**E**. (**C**–**E**) MCCP MKL-1, WaGa, PeTa, and MS-1 cell lines (**C**); MCCP PDCLs MCC-301 and MCC-336 (**D**); and MKL-1 parental, SCR control, AAVS1 control, and p53-KO lines 1-1, 1-2, and 1-3 (**E**) were treated with increasing concentrations of KTX-049 or DS-3032, and CellTiter-Glo assay readout was used to determine cell viability at 48 or 72 h. *N =* 3; data are shown as the mean ± SD.

We previously reported that several MCCP cell lines with WT p53 are highly sensitive to the MDM2 inhibitors milademetan (DS-3032) and Nutlin-3A (31). We also tested the effect of AMG232, a clinical-grade MDM2 inhibitor and found AMG232 to be less potent than DS-3032 in MCC cell lines (Supplemental Figure 1A; supplemental material available online with this article; https://doi.org/10.1172/JCI199049DS1). Here, we therefore compared the activity of DS-3032 with that of the MDM2 degrader KTX-049 in 4 established and 2 patient-derived cell lines (PDCLs). Cells were treated with increasing concentrations of the MDM2 inhibitor or degrader for 24, 48, or 72 h, and cell viability was assessed (Figure 1, A and B). At all 3 time points tested, KTX-049 was more potent than DS-3032 in the WT p53 MCC cell lines MKL-1, WaGa, and PeTa and the PDCLs MCC-301 and MCC-336. MS-1 cells with mutant p53 were resistant to both the MDM2 inhibitor and degrader (Figure 1, C and D, Supplemental Figure 1, B and C, and Table 1).

We determined whether WaGa cells were sensitive to brief exposures to KTX-049 or DS-3032 using a washout assay. Cells were treated with drug for 4, 24, or 48 h followed by extensive washing and refeeding with drug-free media before viability was assessed at 72 h. Treatment with KTX-049 for 4 h decreased cell viability more than continuous treatment with DS-3032 for 24 h. Moreover, 24 or 48 h of treatment with KTX-049, followed by washout, led to IC_50_ values comparable to 72 h of continuous KTX-049 treatment, indicating that MCC WaGa cells were highly sensitive to even brief exposure to KTX-049 (Supplemental Figure 1D and Table 2). Together, these data indicate that MDM2 degradation collapses the p53/MDM2 feedback loop, enabling sustained pathway activation after transient exposure, whereas MDM2 inhibition preserves feedback capacity and requires continuous target engagement. Notably, 4 h of KTX-049 treatment exceeded the viability effect of 24 h of continuous DS-3032 treatment (Table 2), suggesting that brief, intermittent dosing schedules may be sufficient for therapeutic efficacy, a property with potential implications for intermittent clinical dosing.

Among the sensitive cell lines, a gradient of absolute IC_50_ values was observed at 48 and 72 h. Notably, KTX-049 was more than 100 times more potent than DS-3032 in all MCCP cell lines tested (Table 1). Next, we tested the requirement of WT p53 using p53-KO cell lines derived from MCC MKL-1 cells (31). Three MCC MKL-1 p53-KO cell lines but not control cell lines that retained p53 were resistant to both compounds, indicating that both KTX-049 and DS-3032 require the presence of WT p53 (Figure 1E and Table 3). Since a few MCCN tumors contain WT p53 (11, 31, 32), we tested the effect of KTX-049 and DS-3032 on the established WT p53 MCCN cell line UISO. The UISO cells were more sensitive to KTX-049 than DS-3032 but had higher IC_50_ values compared with the established MCCP cell lines (Supplemental Figure 2A and Table 1).

### KTX-049 efficiently degrades MDM2 protein and activates a p53 response.

**Figure 2:**
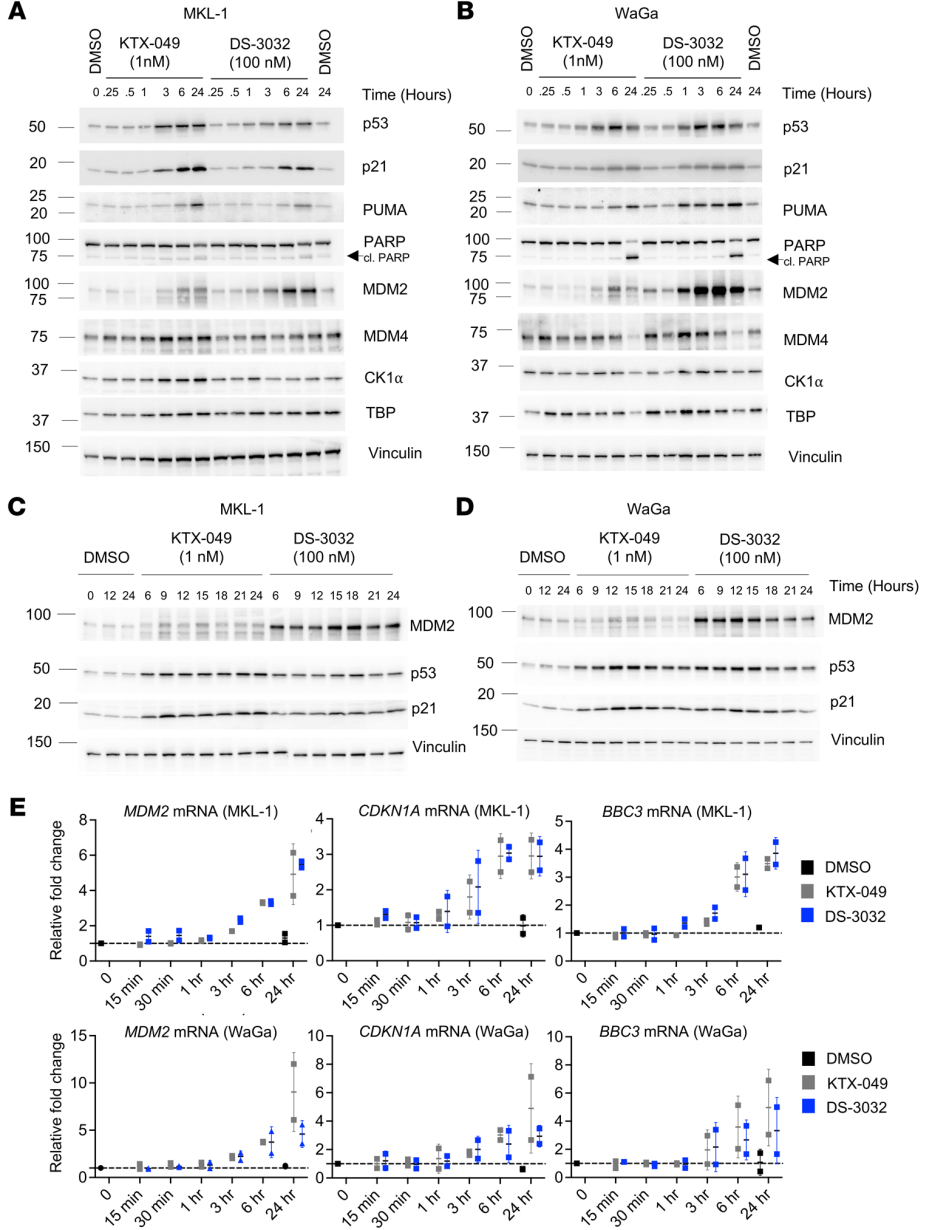
KTX-049 leads to a robust p53 response in MCCP cell lines. (**A** and **B**) MCCP MKL-1 (**A**) and WaGa (**B**) cell lines were treated with DMSO, 1 nM KTX-049, or 100 nM DS-3032, and p53 response was analyzed by Western blotting for the indicated proteins. TBP and Vinculin were used as loading controls. *N =* 2; Western blotting from 1 repeat is shown (see Supplemental Figure 3 for the second repeat). Multiple gels were independently run with an equal volume of the same lysates to analyze the different proteins. The membranes for MDM2 and p21 were stripped and reprobed to analyze levels of Vinculin and PUMA, respectively. (**C** and **D**) Western blotting in MKL-1 and WaGa cells for extended time points. Vinculin was used as a loading control. *N =* 1. (**E**) MKL-1 (top) and WaGa (bottom) cell lines were treated with DMSO, 1 nM KTX-049, or 100 nM DS-3032 followed by analysis of *MDM2*, *CDKN1A*, and *BBC3* mRNA levels by RT-qPCR. Graph indicates relative fold change. *N =* 2; dashed lines indicate mean; data are shown as the mean ± SD.

The effect of the MDM2 degrader KTX-049 and inhibitor DS-3032 on the p53 response in MKL-1 and WaGa cells was assessed by Western blotting and RT-qPCR (Figure 2). To control for the approximately 100-fold difference in potency (Figure 1C and Table 1), cells were treated with 1 nM KTX-049 or 100 nM DS-3032 and compared. The levels of p53 increased within 3 h of treatment in both MKL-1 and WaGa cells (Figure 2, A and B, and Supplemental Figure 3). KTX-049 treatment, but not DS-3032 treatment, led to depletion of MDM2 between 30 min and 1 h (Figure 2, A and B, and Supplemental Figure 3). The p53/MDM2 feedback loop led to higher levels of MDM2 protein with DS-3032 than with KTX-049. MDM2 levels remained lower in KTX-049-treated samples as early as 1 h and up to 24 h of treatment in both cell lines. MDM2 migration patterns differed in response to the degrader compared with the inhibitor (Figure 2, A–D, and Supplemental Figure 3). We further identified that the MDM2 migration patterns are not visible with the inhibitor, as there is increased MDM2 accumulation with the inhibitor, and the separation of different isoforms is limited. We were still able to detect these differences when WaGa cells were treated with proteasomal inhibitors MG132 and Bortezomib, showing that the different isoforms of MDM2 are always present in the cell (Supplemental Figure 4, A and B). However, we did not investigate how the different isoforms of MDM2 contribute to MDM2 function. Pretreatment with proteasomal inhibitors also abolished the loss of MDM2 by KTX-049 (Supplemental Figure 4B). Additionally, the mRNA levels of *MDM2* increased following KTX-049 treatment in both MKL-1 and WaGa cell lines (Figure 2E). Collectively, these data indicate that KTX-049 acts on the *MDM2* protein and not mRNA. In both cell lines, a time-dependent increase in the mRNA and protein levels of downstream effectors of p53 protein, including p21 (*CDKN1A*), PUMA (*BBC3*), and MDM2, was observed following treatment with KTX-049 and DS-3032, indicating an active p53 response (Figure 2, A–E).

Next, we compared p53 response in WaGa cells after brief exposure to 0.1 or 1 nM KTX-049 for 2, 4, or 8 h followed by washout and collection of cells at 24 h. The 1 nM dose of KTX-049 led to a higher accumulation of p53, p21, PUMA, and cleaved PARP than the 0.1 nM dose. Interestingly, an increase in p53, p21, PUMA, and cleaved PARP levels was observed even after a short exposure, indicating that the cells could execute an active p53 response and undergo apoptosis even after treatment for just a few hours (Supplemental Figure 5A). An active p53 response was not observed in p53-KO cell lines (Supplemental Figure 5B). Notably, MDM2 was depleted by KTX-049 in MKL-1 p53-KO cell lines at 1 h but did not increase at 24 h. These results are consistent with the absence of a p53 response (Supplemental Figure 5B).

The ST-MYCL-Tip60 complex transactivates *MDM2* and CK1α (casein kinase 1 alpha 1, *CSNK1A1*), which can affect the activity of MDM4 (2, 14, 20, 32). Since MDM4 and CK1α cooperate with MDM2 in MCCP, we analyzed the protein levels of MDM4 and CK1α. We did not detect an effect on CK1α or MDM4 with DS-3032 or KTX-049 in MKL-1 cells. However, both KTX-049 and DS-3032 decreased MDM4 levels in WaGa and UISO cells at the 24 h time point independent of CK1α (Figure 2, A and B, Supplemental Figure 2B, Supplemental Figure 3, and Supplemental Figure 5, A and B). This MDM4 reduction is consistent with prior reports that p53 activation can suppress MDM4 expression, although the mechanism was not directly tested here (32).

### KTX-049 disruption of the p53/MDM2 feedback loop leads to increased potency.

**Figure 3:**
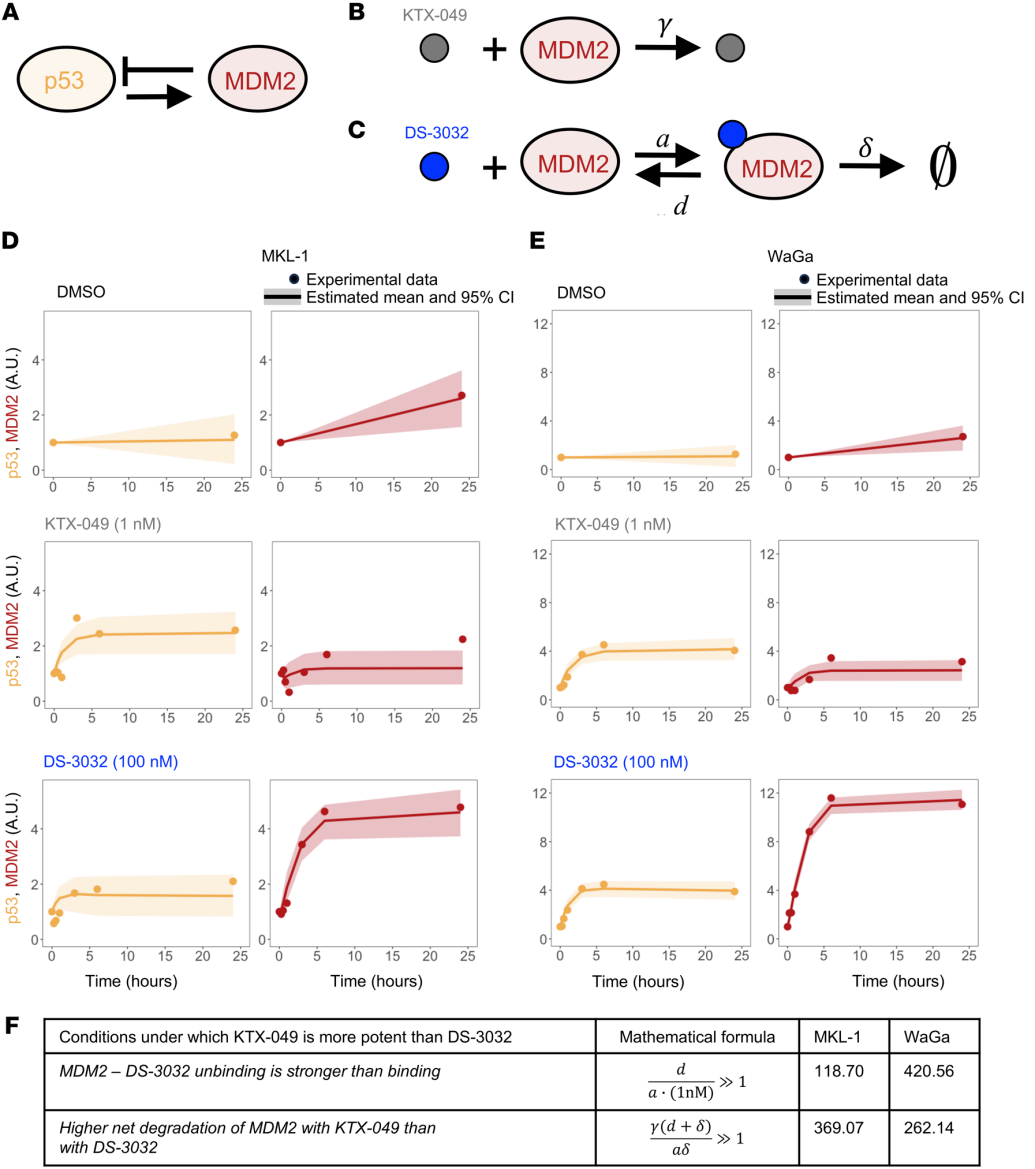
Mathematical modeling shows that KTX-049 collapses the p53/MDM2 feedback loop, making it more potent than DS-3032. (**A**) Diagram of the p53/MDM2 feedback loop circuit. See Supplemental Figure 6A for the reactions and ODEs associated with this diagram. (**B**) Diagram illustrating the effect of KTX-049 on MDM2. See Supplemental Figure 6B for the reactions and associated ODEs. (**C**) Diagram illustrating the effect of DS-3032 on MDM2. See Supplemental Figure 6C for the reactions and associated ODEs. (**D** and **E**) Observed experimental data (dots) and estimated trajectories (solid lines and shaded areas representing the mean and 95% credible interval [CI], respectively) of p53 and MDM2 under DMSO, 1 nM KTX-049, or 100 nM DS-3032 treatment in the MCCP MKL-1 (**D**) and WaGa (**E**) cell lines. The data used for the parameter estimation and shown in these panels correspond to those in Figure 2, A and B. (**F**) Validation of the conditions under which KTX-049 is more potent than DS-3032 for the data in Figure 2, A and B. More precisely, we have *a* = 0.049 nM^–1^ h^–1^, *d* = 5.75 h^–1^, *δ* = 0.92 h^–1^, and *γ* = 2.46 nM^–1^ h^–1^ for MKL-1 and *a* = 0.015 nM^–1^ h^–1^, *d* = 6.13 h^–1^, *δ* = 2.88 h^–1^, and *γ* = 1.22 nM^–1^ h^–1^ for WaGa.

To understand why KTX-049 was more potent than DS-3032, we developed a mechanistic mathematical model that captures the key interactions among MDM2, p53, KTX-049, and DS-3032. This model was parameterized using dose-response data (Figure 2, A and B) to assess the effect of each drug on MDM2 and p53 dynamics. This model includes the induction of *MDM2* transcription by p53 and degradation of p53 by MDM2, forming a negative feedback loop (Figure 3A and Supplemental Figure 6A), which is a well-established circuit for studying the dynamics of these proteins (33–35). This modeling framework formalizes the experimentally observed distinction between feedback-limited p53 activation by MDM2 inhibition and feedback collapse induced by MDM2 degradation.

In our model, KTX-049 acted as an MDM2 degrader, introducing an additional degradation rate for MDM2 (Figure 3B and Supplemental Figure 6B) (36). Conversely, DS-3032 functions as an MDM2 inhibitor, sequestering MDM2 and preventing its interaction with p53 (35, 36). This effect was modeled through a binding-unbinding reaction, forming a DS-3032–MDM2 complex that cannot interact with p53 (Figure 3C and Supplemental Figure 6C).

In the context of our model, the increased potency of KTX-049 compared with that of DS-3032 can be attributed to the stronger unbinding of the DS-3032–MDM2 complex, which releases a notable amount of MDM2 back into the system. This phenomenon resulted in a lower net removal rate of MDM2 during treatment with DS-3032 than with KTX-049. Consequently, the amount of MDM2 available to bind p53 was considerably reduced by KTX-049, effectively disrupting the feedback loop.

To validate this hypothesis, we estimated the rate constants of our models (Supplemental Figure 6) using experimental data (Figure 2, A and B) via Bayesian statistical inference (see Methods) (37). The estimated parameters accurately reflected the MDM2 and p53 dynamics observed experimentally for DMSO, KTX-049, and DS-3032 in both the MKL-1 and WaGa cell lines (Figure 3, D and E). The parameters confirmed that DS-3032–MDM2 unbinding was stronger than binding and that the net removal rate of MDM2 with DS-3032 was lower than that with KTX-049, supporting our hypothesis (Figure 3F). We repeated this analysis with 2 additional experiments (Supplemental Figure 3 and Figure 2, C and D, for experimental data; Supplemental Figure 7 for computational results), consistently obtaining similar results and further validating our hypotheses. Sensitivity analysis indicated that a decrease of up to 20% in the initial drug concentrations by the end of the experiment did not considerably affect the estimated trajectories (Supplemental Figure 8).

### KTX-049 and DS-3032 induce apoptosis in WT p53 MCC.

**Figure 4:**
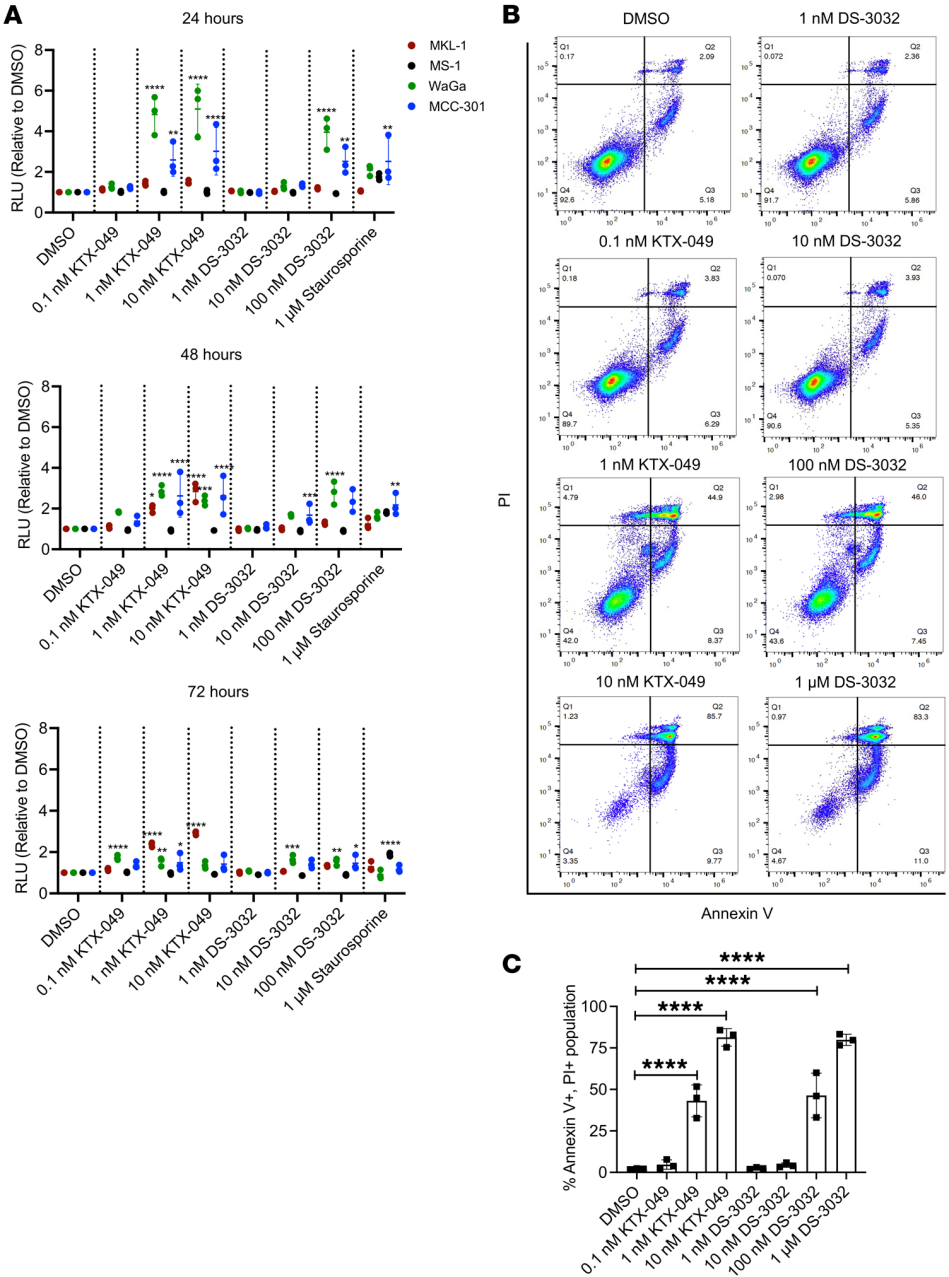
KTX-049 induces apoptosis in MCCP cell lines. (**A**) MKL-1, WaGa, MS-1, and MCC-301 cells were treated with DMSO or indicated concentrations of KTX-049, DS-3032, or staurosporine, and Caspase-Glo 3/7 assay was performed at 24, 48, and 72 h. Graphs indicate relative luminescence compared with DMSO control. *N =* 3; data are shown as the mean ± SD. 2-way ANOVA with Tukey’s multiple-comparison was performed. Significance was calculated comparing KTX-049 or DS-3032 treatment with DMSO treatment for the respective cell line. ****P <0.0001, ***P <0.001, **P <0.01, *P < 0.05. (**B** and **C**) MCCP WaGa cells were treated with DMSO or indicated concentrations of KTX-049 or DS-3032 for 24 h, and annexin V/PI staining was performed. Data from a single representative experiment are shown in B. *N =* 3 in C; data are shown as the mean ± SD. Annexin V+, PI+ shows late apoptotic or dead cells. 2-way ANOVA with Tukey’s multiple-comparison test was performed. Significance for DMSO treatment compared with the respective KTX-049 or DS-3032 treatment is shown. ****P < 0.0001.

Along with an increase in the downstream effectors of p53 in response to KTX-049 and DS-3032 treatment as reported above, we observed increases in the levels of apoptotic markers PUMA and cleaved PARP in the MCCP MKL-1 and WaGa cell lines, indicating an apoptotic response (Figure 2, A and B, and Supplemental Figure 3). In contrast, although the MCCN cell line UISO showed an active p53 response to either KTX-049 or DS-3032, we did not detect cleaved PARP (Supplemental Figure 2B). To confirm the pro-apoptotic response in MCCP cell lines, we used the Caspase-Glo 3/7 assay to test MKL-1, WaGa, MCC-301, and MS-1 cells for caspase-3/7 activity after treatment with KTX-049, DS-3032, or staurosporine as a positive control for 24, 48, and 72 h. KTX-049 and DS-3032 showed a dose-dependent increase in caspase activity across the sensitive cell lines MKL-1, WaGa, and MCC-301 but no activity in the p53-mutant MS-1 cell line (Figure 4A). Similar to the viability assays, we observed 100-fold more potent pro-apoptotic activity of KTX-049 than that of DS-3032 (Figure 1, C and D, Table 1, and Figure 4A). Notably, different MCCP cell lines showed peak caspase-3/7 activity at different times. WaGa and MCC-301 cell lines had peak caspase-3/7 activity at 24 h, with 4- to 5-fold (WaGa) and 2.5- to 3-fold (MCC-301) significantly increased caspase activity in KTX-049– and DS-3032–treated cells compared with their DMSO controls. MKL-1 cells had peak caspase-3/7 activity 3-fold greater than the DMSO control 48 h after treatment with KTX-049. After 72 h of treatment, the cells had lower caspase-3/7 activity compared with the levels at 24 h for WaGa and MCC-301 cells, compared with the levels at 48 h for MKL-1 cells (Figure 4A). As the absence of cleaved PARP in the MCCN cell line UISO indicated lack of caspase activity, we did not screen this cell line for the activation of caspase-3/7 with the Caspase-Glo 3/7 assay (Supplemental Figure 2B). Despite induction of p53 target genes, UISO cells failed to undergo apoptosis, consistent with intrinsic apoptotic resistance in this MCCN context.

Next, we performed annexin V/propidium iodide (PI) staining of WaGa cells treated with KTX-049 or DS-3032. Cells treated with 1 or 10 nM KTX-049 showed apoptotic responses similar to those observed with 100 nM or 1 μM DS-3032, respectively (Figure 4, B and C). We also tested if cells dual positive for annexin V and PI can be detected at earlier time points after treatment with 1 nM KTX-049 or 100 nM DS-3032. However, we observed maximal effect at 24 h (Supplemental Figure 9).

Next, we assessed the effects of KTX-049 and DS-3032 on the cell cycle of WaGa cells by EdU and DAPI staining. Compared with DMSO, treatment with KTX-049 or DS-3032 for 6 h reduced the fraction of cells in the S phase and cells accumulated in the G_0_/G_1_ phase. After 12 h, there were significantly fewer cells in the S phase of the cell cycle and an increased number of cells in the G_0_/G_1_ phase (Supplemental Figure 10, A and B).

### Proteomic analysis reveals a strong p53 response induced by KTX-049 and DS-3032.

**Figure 5:**
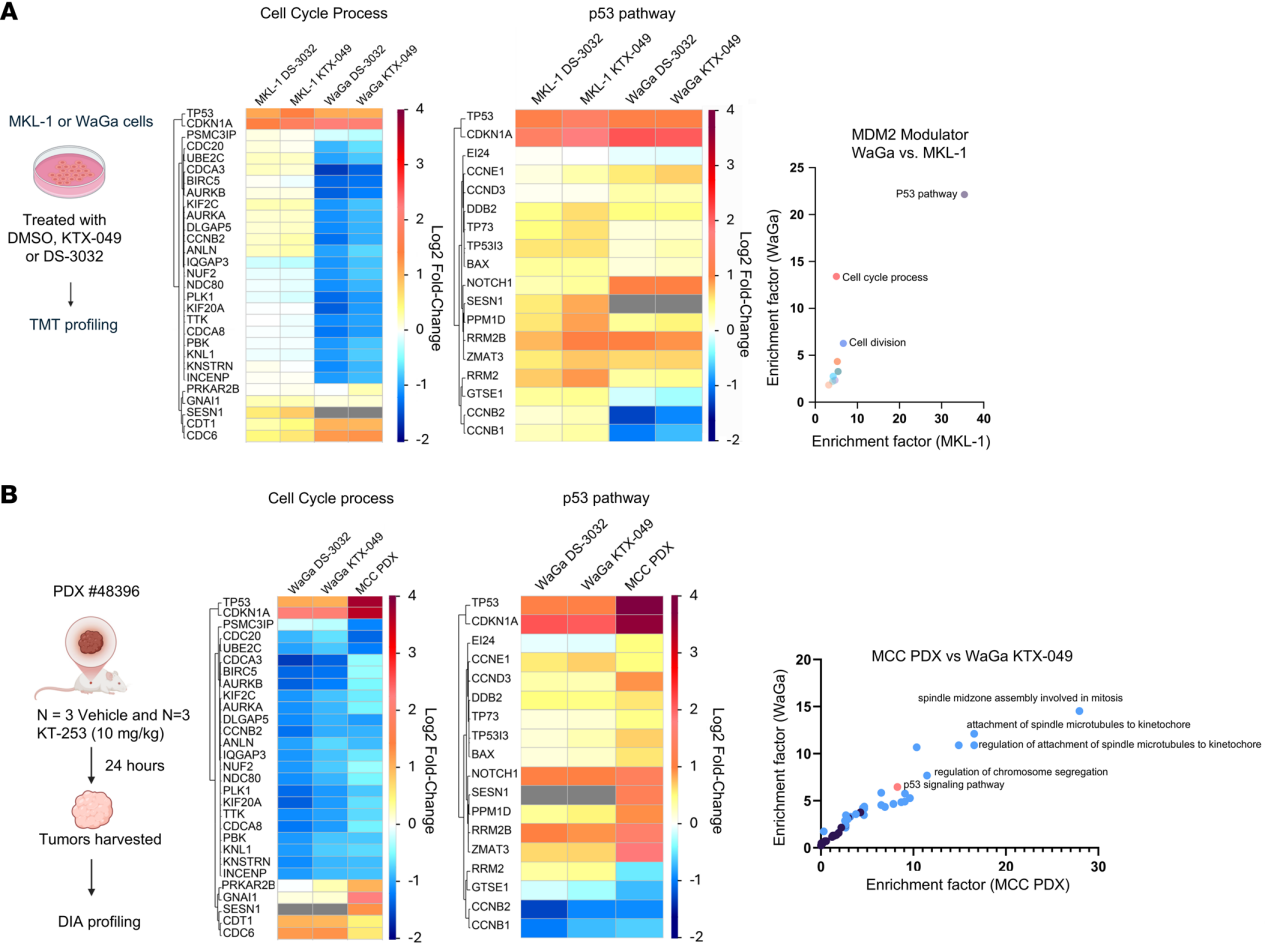
Global proteomics reveals that KTX-049 and KT-253 induce increased levels of direct p53 targets. (**A**) Scheme (left) showing WaGa and MKL-1 cells were treated with DMSO, KTX-049, or DS-3032 followed by TMT profiling. Heatmaps showing the log_2_(fold change) from MKL-1 and WaGa cells treated with KTX-049 or DS-3032 highlight the most significantly regulated proteins with Gene Ontology Biological Process (GOBP) cell cycle process annotation (middle) or with the KEGG p53 signaling annotation (right). The cell cycle process heatmap was filtered to include only proteins with log_2_(fold change) > 1 or < –1, while the p53 signaling heatmap was filtered to include only proteins with log_2_(fold change) > 0.5 or < –0.5. NOTCH1 is not a KEGG p53 signaling pathway protein but is a direct p53 target (38) and was also included in the heatmap. A pathway analysis was performed for WaGa and MKL-1 cells, and the enrichment factors for identified GOBP and KEGG terms common to both cell lines were plotted as a scatterplot (right). (**B**) Mice implanted with PDX 48396 were treated with vehicle or KT-253 for 24 h before tumor harvesting, processing, and analysis by DIA-based global proteomics (left). The corresponding heatmaps highlight the most regulated proteins with the GOBP cell cycle process annotation (middle) or KEGG p53 signaling annotation (right), with log_2_(fold change) filtering criteria as described above. Both heatmaps show the direct comparison of log_2_(fold change) between WaGa cells and PDX 48396 (referred to as MCC PDX) treated with KT-253. As above, a pathway analysis was performed to determine significantly enriched GOBP and KEGG annotation terms, and the enrichment factors for terms identified for WaGa and the PDX 48396 model were plotted as a scatterplot (**B**, below). Terms associated with mitosis or cell cycle are in blue, those associated with p53 signaling are in red, and all others are purple.

To assess the impact of KTX-049 and DS-3032 on the proteome of WaGa and MKL-1 cells, lysates were collected after 1 or 24 h of treatment and tandem mass tag-based (TMT-based) proteome profiling was performed. Proteome-wide changes were observed at 24 h but not after 1 h of treatment with KTX-049 or DS-3032 compared with the baseline control (Figure 5A and Supplemental Figure 11). The top differentially expressed proteins included *CDKN1A* (p21) and *TP53* (p53), consistent with the Western blotting results shown in Figure 2 and Supplemental Figure 3. Many additional, highly validated (38), proteins encoded by p53 response genes were upregulated in both MKL-1 and WaGa cell lines, including RRM2B, PPM1D, and NOTCH1 (Figure 5A and Supplemental Figure 11) (38). Increased levels of NOTCH1 were confirmed by Western blotting in MKL-1 cells treated with DS-3032 or KTX-049 (Supplemental Figure 12). Pathway analysis of the proteome revealed significant enrichment of the p53 signaling pathway with KTX-049 and DS-3032 in MKL-1 cells and KTX-049 treatment in WaGa cells (Supplemental Figure 13, A and B). Notably, mitotic proteins were downregulated in response to KTX-049 and DS-3032 in WaGa cells but not to the same extent in MKL-1 cells (Figure 5A and Supplemental Figure 13). These differences may reflect the fact that WaGa cells were already undergoing apoptosis and had an impaired cell cycle in response to treatment for 24 h (Figure 2 and Supplemental Figure 10).

### KT-253 is highly effective in MCC patient- and cell line–derived xenograft models.

We then assessed the clinical-grade MDM2 degrader KT-253 for in vivo studies of MCC (27). To assess the impact of the MDM2 degrader on the tumor proteome, a single dose of KT-253 was administered to 3 animals in the MCC patient-derived xenograft (PDX) 48396 model, and data from these mice were compared with 3 tumor-bearing animals receiving vehicle treatment. Tumors were harvested 24 h after treatment, and data-independent acquisition–based (DIA-based) proteome profiling was performed. Tumors from KT-253–treated animals revealed a robust p53 response that was comparable to the p53 response to KTX-049 in vitro. In addition to p53 response, mitotic protein levels were significantly downregulated in vivo, similar to those observed in the WaGa in vitro model (Figure 5B and Supplemental Figure 14).

**Figure 6:**
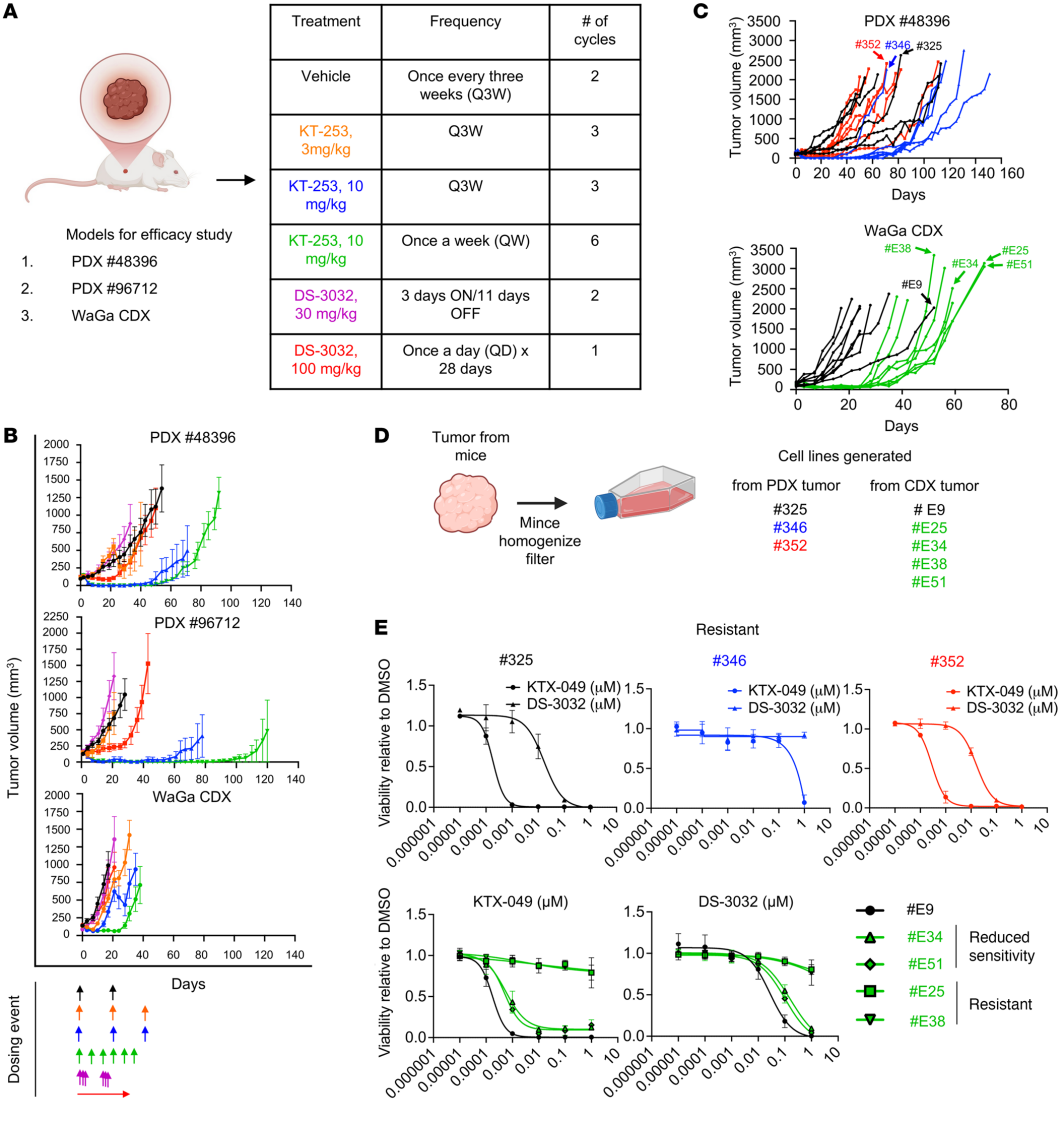
KT-253 is highly effective in MCCP models with WT p53 in vivo. (**A**) Scheme shows in vivo models used, and table indicates dosing groups used for PDX and CDX studies. (**B**) Average tumor volumes plotted for PDX 48396 when 5–6 mice were alive per treatment group, PDX 96712 when all 6 mice were alive per treatment group, and WaGa CDX model when all 8 mice were alive per treatment group. Dosing event is denoted by arrows below, and colors match dosing groups shown in **A**. (**C**) Individual tumor trajectories for the PDX 48396 model (above) and the WaGa CDX model (below). Colors indicate treatment groups shown in **A**. Individual tumors from which cells were derived are marked with arrows. (**D**) Scheme shows cell lines derived from tumors in the in vivo models. (**E**) Cell lines derived from the PDX 48396 model (above) or the WaGa CDX model (below) were treated with different concentrations of KTX-049 or DS-3032, and CellTiter-Glo assay readout was used to determine cell viability at 72 h. *N =* 3; data are shown as the mean ± SD. Colors indicate in vivo treatment groups from which cells were derived.

We compared the in vivo efficacy of the KT-253 MDM2 degrader with that of the inhibitor DS-3032 in 2 PDX MCC models and 1 cell line–derived xenograft (CDX; WaGa) MCC model (Figure 6, A and B). PDX models 48396 and 96712 have been previously described and found to be highly sensitive to DS-3032 (31). These PDX tumors were not treatment naive. Prior treatment information of patient tumors from whom the PDXs were derived was also previously reported (39). Tumor-bearing mice were randomly assigned to the following treatment groups: (a) vehicle once every 3 weeks (Q3W) for 2 cycles, (b) KT-253 (3 mg/kg) Q3W for 3 cycles, (c) KT-253 (10 mg/kg) Q3W for 3 cycles, (d) KT-253 (10 mg/kg) once a week (QW) for 6 weeks, (e) DS-3032 (30 mg/kg) once a day (QD) for 3 days on and 11 days off for 2 cycles, and (f) DS-3032 (100 mg/kg) QD for 28 days (Figure 6A). The intermittent schedule for DS-3032 was similar to that used in an acute lymphocytic leukemia xenograft model (27).

Treatment with DS-3032 at 30 mg/kg QD had no considerable effect compared with the vehicle control in the PDX models. The higher dose of DS-3032 (100 mg/kg/QD) led to a significant reduction in the mean tumor volume compared with the vehicle control (Figure 6B, Supplemental Figure 15B, and Tables 4–7). KT-253 at a lower dose (3 mg/kg Q3W) showed some response in each cycle before the tumors began to regrow. However, the average tumor volume showed no marked difference compared with the vehicle control on day 21 (PDX 96712) or day 33 (PDX 48396), but the tumors rebounded immediately and there was no long-term response at this low dose of KT-253 in the PDX models (Tables 4–7 and Supplemental Figure 15, A and B). The higher dose of KT-253 (10 mg/kg) with either the QW or the Q3W dosing schedule demonstrated significant tumor growth inhibition with regressions in both PDX models (Figure 6, B and C, Supplemental Figure 15, A and B, and Tables 4–7). Remarkably, in the PDX 96712 model, at least 3 of the 6 mice were tumor free in both KT-253 treatment groups 10 mg/kg Q3W and QW at the time of study termination (days 148–214) (Supplemental Figure 15, A and B). In the other PDX 48396 model, all tumors eventually regrew after treatment was stopped (Figure 6, B and C, and Supplemental Figure 15, A and B). We did not observe any striking BW changes in the mice, indicating the absence of treatment-induced toxicities (Supplemental Figure 16).

In the WaGa CDX xenograft subcutaneous model, DS-3032 at 30 or 100 mg/kg was comparable to that of the vehicle control (Figure 6B and Tables 8 and 9). KT-253 at 3 mg/kg Q3W was modestly active with some regression, but the tumors rebounded quickly, similar to the PDX models. KT-253 at 10 mg/kg led to a significant reduction in tumor volume, with QW dosing being more effective than Q3W (Figure 6B, Supplemental Figure 15, A and B, and Tables 8 and 9).

### Acquired resistance to the MDM2 degrader.

In the PDX model 48396 treated with KT-253 at 10 mg/kg Q3W, we observed that the tumor in mouse 346 regrew faster than tumors in other mice within the same treatment group (Figure 6C and Supplemental Figure 15, A and B). To gain insight into the apparent resistance to the MDM2 degrader, the tumor was harvested at the endpoint and a cell line was established in vitro using a previously described protocol (39) (Figure 6D). The control cell lines were derived from tumors harvested from mice treated with vehicle (cell line 325) or DS-3032 (cell line 352) (Figure 6, C and D). We treated the 3 cell lines with KTX-049 or DS-3032 for 72 h and assessed their effect on cell viability in vitro. Control cell lines 325 and 352 were highly sensitive to both treatments with a similar range of absolute IC_50_ values as the MCC-301 cell line derived from the same PDX model (Figure 6E and Tables 1 and 10). In contrast, the cell line 346 did not respond to DS-3032 and had a minimal response to KTX-049 in vitro, indicating that it was resistant to MDM2 perturbation (Figure 6E and Table 10).

Next, we performed Western blotting to assess the p53 response of the cell line 346 to KTX-049 and DS-3032 compared with that of the control cell lines 325 and 352. As shown in Supplemental Figure 17A, tumors 325 and 352 responded to KTX-049 and DS-3032, with increased levels of p21, PUMA, MDM2, and cleaved PARP. However, 346 showed much lower levels of p21, PUMA, cleaved PARP, and MDM2 at baseline, and levels did not increase with treatment. Notably, 346 had consistently higher MDM4 levels compared with 325 or 352 (Supplemental Figure 17A).

To gain insight into the mechanism underlying the reduced sensitivity of cell line 346, we performed whole-exome sequencing (WES) and used MCC-301 and 325 cell lines as controls (Supplemental Figure 18, A and B). A *BCOR* nonsense mutation (p.R1514X) was identified in MCC-301, 325, and 346. The BCOR mutation in MCC-301 has been reported previously (39). This mutation was not detected in the matched germline control (c-301), indicating that it is an acquired somatic mutation (Supplemental Figure 18A). The 346 cell line, resistant to KTX-049 and DS-3032 treatment, contained 3 *TP53* mutations: 1 at a high allelic frequency (AF) (p.R282Q, AF = 0.662) and 2 (p.R175H and p.E298X) at lower levels (AF = 0.038 and 0.205) (Supplemental Figure 18, A and B). Mutations in ARID1B and MTOR were detected at low frequencies (Supplemental Figure 18A).

The WaGa CDX MCC tumor model demonstrated regrowth during ongoing KT-253 treatment at every dose and schedule, suggesting acquired resistance (Figure 6, B and C, and Supplemental Figure 15, A and B). Using the approach described above, we established 4 cell lines, E25, E34, E38, and E51, derived from tumors harvested at the endpoint of the KT-253 treatment group and 1 cell line, E9, from a tumor in the vehicle-treated group (Figure 6, C and D). Notably, the tumors in the E9 mice grew much later than the other tumors in the vehicle-treated group (Figure 6C and Supplemental Figure 15, A and B). Consistent with this delayed growth, the E9 cell line showed reduced cleaved caspase-3 induction compared with the parental WaGa line in response to both drugs (Supplemental Figure 19B), indicating that in vivo xenograft passaging itself selects for partial apoptotic tolerance independent of drug treatment and suggesting that the degree of acquired resistance in KT-253–treated CDX lines may be measured against a baseline that is already shifted relative to the parental cell line.

Five cell lines derived from the WaGa CDX model were treated with KTX-049 or DS-3032 for 72 h. The E9 cell line derived from a tumor in the vehicle-treated group was sensitive to both KTX-049 and DS-3032 in vitro with absolute IC_50_ values comparable to those of the parental WaGa cell line (Figure 1, Figure 6E, and Tables 1 and 11). Two of the cell lines derived from tumors in the KT-253–treated group, E34 and E51, had reduced sensitivity to KTX-049 and DS-3032 in vitro with approximately 5-fold higher absolute IC_50_ values compared with the E9 control. The cell lines E25 and E38 were completely resistant to treatment in vitro (Figure 6E and Table 11).

We performed Western blotting analysis of WaGa CDX-derived cell lines treated with KTX-049 or DS-3032 in vitro. For all 5 cell lines, we observed that MDM2 levels were reduced in the presence of KTX-049. The E9 control group showed higher MDM4 levels than the parental WaGa group did. However, only the E9, E34, and E51 cell lines, but not E25 and E38, responded with increased levels of p53, p21, and PUMA. Notably, E25 and E38 had higher basal levels of p53 than other cell lines (Supplemental Figure 17B). Only the E9 control line showed increased levels of cleaved PARP and cleaved caspase-3 in response to both drugs. We then assessed the ability of WaGa CDX-derived lines to undergo apoptosis using annexin V/PI staining after treatment with DMSO, KTX-049, or DS-3032 for 48 h. The E9 cell line had reduced levels of apoptotic cells compared with the WaGa parental control. In contrast, the E34, E51, E25, and E38 cell lines showed a complete lack of apoptosis in response to either KTX-049 or DS-3032 (Supplemental Figure 19A). We compared the vehicle control line E9 with the parental WaGa cell line and observed decreased levels of cleaved caspase-3 in response to KTX-049 or DS-3032 treatment (Supplemental Figure 19B).

We then performed WES on the parental WaGa cell line and 5 WaGa CDX-derived cell lines to assess acquired mutations. An oncogenic mutation in *TP53* (p.F270C) was detected at low AF of 0.082 in the WaGa parental cell line. This mutation was enriched at higher allelic frequencies in resistant cell lines E25 (AF = 0.737) and E38 (AF = 0.532). E38 cells contained 2 additional *TP53* mutations (p.R273P, AF = 0.096; p.C135F, AF = 0.034) and a mutation in *RUNX1*. The partially sensitive cell lines E34 and E51 had 2 different low-frequency mutations detected in *TP53*, namely, p.S241F (AF = 0.305 and 0.082, respectively) and p.N239D (AF = 0.096 and 0.25, respectively). E34 cells also had an additional mutation in *TP53* (p.F270C) at a low AF (Supplemental Figure 18, A and B).

**Figure 7:**
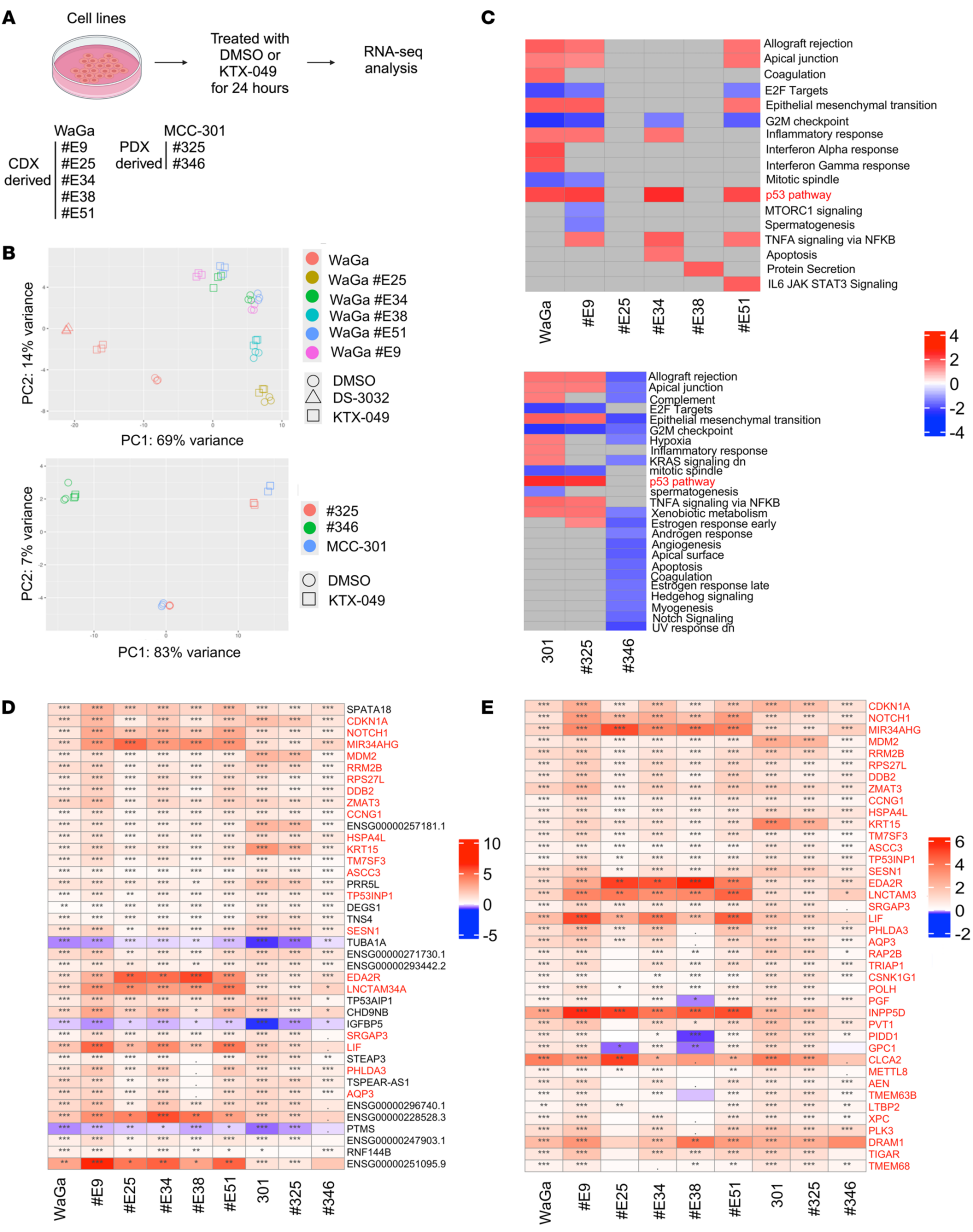
Impaired transcriptional activation of a distinct set of direct p53 target genes correlates with resistance to MDM2 degrader. (**A**) Cell lines used for RNA-seq analysis. (**B**) Principal component analysis plots for WaGa and WaGa CDX cell lines (top) and MCC-301 and PDX 48396 cell lines (bottom). Note that only the WaGa cell line was treated with both KTX-049 and DS-3032. (**C**) Heatmaps of normalized enrichment scores from GSEA for WaGa and WaGa CDX cell lines (top) and MCC-301 and PDX 48396 cell lines (bottom). The p53 pathway is highlighted in red. (**D**) Top 40 differentially expressed genes in KTX-049 treated samples compared with the respective DMSO controls are shown based on how often a gene was significant (*q* value). Direct p53 targets with a target gene reg score of ≥35 are shown in red. (**E**) Heatmap shows top 40 differentially expressed direct p53 targets genes (by *q* value) in KTX-049–treated samples compared with their respective DMSO controls. Adjusted *P* values were calculated by fgsea based on an adaptive, multilevel, split Monte-Carlo scheme. ***P < 0.001, **P < 0.01, *P < 0.05, *P* < 0.1.

To assess the impact on the transcriptome, we performed RNA-seq of MKL-1 and WaGa cells treated with DMSO, KTX-049, or DS-3032 and PDX and CDX cell lines treated with DMSO or KTX-049 in vitro (Figure 7A and Supplemental Figure 20A). Principal component analysis revealed that MKL-1 DMSO-treated cells clustered distinctly from KTX-049– or DS-3032–treated cells (Supplemental Figure 20B). The WaGa parental cell line also showed differences with treatment, but all WaGa parental samples clustered distinctly from the WaGa CDX-derived cell lines (Figure 7B), consistent with our observation that E9 behaved differently from the WaGa parental cells (Supplemental Figure 19, A and B). Interestingly, KTX-049–treated samples from MCC-301 and 325 cell lines formed a distinct cluster, and DMSO-treated samples from both cell lines formed another distinct cluster (Figure 7B). Conversely, 346 KTX-049– and DMSO-treated samples clustered together, clearly distinct from both MCC-301 and 325 (Figure 7B). Significant pairwise comparisons and GSEA showed that in both MKL-1 and WaGa cells, direct p53 response genes were upregulated after treatment with KTX-049 or DS-3032 (Supplemental Figure 20, C–E). GSEA showed significant enrichment of the p53 pathway genes in WaGa, E9, E34, E51, MCC-301, and 325 cell lines but not in the resistant E25, E38, or 346 cell lines (Figure 7C). Differential gene expression revealed that several genes were similarly upregulated or downregulated with treatment across the different cell lines, and the resistant cell lines did not completely lack the activation of p53 targets (Figure 7D and Supplemental Figure 21, A and B). Moreover, several direct p53 response genes were differentially regulated across cell lines. The direct p53 target gene *EDA2R* was significantly upregulated in response to KTX-049 treatment in the resistant E25 and E38 cell lines as well as in the less sensitive E34 and E51 cell lines when compared with the levels in the WaGa and E9 cell lines (Figure 7, D and E). However, similar to the WaGa analysis, 346 did not show upregulation of *EDA2R* in response to KTX-049 treatment (Figure 7, D and E, and Supplemental Figure 21, A and B). *GPC1* expression was downregulated after treatment in resistant E25 and E38 cell lines. Similarly, the resistant 346 cell line also displayed downregulation of the direct p53 target gene *GPC1* compared with MCC-301 and 325 controls (Figure 7E and Supplemental Figure 21B). Additionally, the resistant 346 cell line showed lower expression of several *TP53* target genes than the MCC-301 and 325 controls. Some of these genes were *FAS*, *SERPINE1*, *TAP1*, *GASK1B*, *EPHA2*, *TRIM22*, *PLEKHG1*, *TNFRSF10B*, and *PROCR* (Figure 7, D and E, and Supplemental Figure 21, A and B). Collectively, these data reveal 2 mechanistically distinct resistance phenotypes: in the PDX-derived 346 line, dominant *TP53* mutations abolish p53 transcriptional activity broadly, whereas in the CDX-derived E25 and E38 lines, p53 retains partial transcriptional competence, as evidenced by *EDA2R* upregulation, but fails to execute the apoptotic program, suggesting that resistance can arise either at the level of p53 itself or downstream at the level of apoptotic effector engagement.

## Discussion

Our study demonstrated that both the MDM2 inhibitor DS-3032 and the degrader KTX-049 were highly specific and effective in activating the p53 response in MCC (Figures 2 and 5). The remarkable potency of KTX-049 and KT-253, as revealed in our study, was attributed, at least in part, to their ability to effectively disrupt the p53/MDM2 feedback loop (Figure 3). MDM2 inhibitors such as DS-3032 often face limitations owing to this feedback mechanism, which can undermine their efficacy. Our findings, supported by mathematical modeling, indicate that MDM2 degraders can circumvent these limitations by reducing MDM2 levels more effectively, thereby enhancing p53 response. This disruption enables sustained p53 activation and apoptosis. More broadly, these findings establish feedback architecture as a determinant of therapeutic response and position MDM2 as a case in which protein degradation overcomes limitations of inhibition in autoregulated circuits.

We previously reported that MDM2 inhibition is a promising strategy for reactivating *TP53* in MCC tumors with WT *TP53* (31, 32). Notably, a phase Ib/II study in patients with WT p53 MCC treated with the MDM2 inhibitor navtemadlin (KRT-232, AMG232) with or without anti-PD1/anti–PD-L1 has recently been completed (ClinicalTrials.gov NCT03787602), and the results will be reported soon (40). In general, the high frequency of hematological and gastrointestinal adverse events has limited the clinical usefulness of MDM2 inhibitors (24, 25, 40). Therefore, there is a need to develop and test potent inhibitors with fewer adverse effects. Importantly, the responses to the MDM2 inhibitor DS-3032 and degraders KTX-049 and KT-253 were highly similar when assessed by Western blotting, RNA-seq, and total proteomics in vitro and in vivo. While MDM2 inhibition and degradation engage overlapping p53 transcriptional programs, they differ substantially in magnitude, durability, and sensitivity to feedback regulation. These findings suggest that the dominant biological activity of the degraders reflects engagement of the p53/MDM2 axis rather than additional off-target effects.

Several MDM2 degraders have recently been developed and shown activity in preclinical models of leukemia and breast cancer (27–30). KT-253, a Cereblon-recruiting MDM2 degrader, has been reported to be more effective at picomolar concentrations than nanomolar or low micromolar concentrations reported for other degraders (27–29). KT-253 is currently in a phase I trial for high-grade myeloid malignancies, ALL, lymphoma, and solid tumors, including MCC (ClinicalTrials.gov NCT05775406) (27). MDM2 degraders may also be relevant in other WT TP53 cancers, but this requires context-specific evaluation.

Despite these promising results, our study highlights the challenge of drug resistance, which is primarily associated with TP53 mutations. This resistance underscores the critical role of WT p53 in the efficacy of MDM2-targeted therapies. WES analysis of resistant tumors revealed enrichment of TP53 mutations, which were sufficient to confer resistance or reduced sensitivity to MDM2 degraders (Supplemental Figure 18). This finding emphasizes the need for a thorough assessment of p53 status, which could be crucial for stratifying patients and tailoring treatment plans. Notably, the primary mode of resistance observed with KT-253 was the enrichment of *TP53* mutations, implicating the p53/MDM2 axis as the primary mode of activity. Similarly, when comparing the transcriptome and proteome responses of the MDM2 degrader to the inhibitor, we found obvious overlap with a few differences, implying that the degrader’s mechanism of action, activating p53, did not considerably differ from that of the inhibitor (Figures 5 and 7). These findings indicate that acquired resistance reflects loss of downstream p53 transcriptional competence rather than escape from MDM2 degradation itself. Importantly, resistance did not arise from failure of MDM2 degradation but instead reflected loss of effective p53 transcriptional competence across critical apoptotic and cell cycle programs, thereby confirming that degrader efficacy depends on intact pathway execution rather than target engagement alone.

Clinical development of MDM2 degraders will require stratification by TP53 status. Because acquired TP53 mutations confer cross-resistance to MDM2 degraders and inhibitors (Table 10), MDM2 degraders are unlikely to overcome resistance driven by acquired TP53 mutations after prior MDM2 inhibitor therapy. This approach would be particularly beneficial in regions with high UV exposure and predominance of MCCN tumors, where WT *TP53* is uncommon.

Our data reveal 2 mechanistically distinct resistance phenotypes that point toward specific combination strategies. In the 346 PDX-derived line, dominant *TP53* mutation abolishes p53 function broadly; in the CDX-derived E25 and E38 lines, p53 retains partial transcriptional activity but fails to commit to apoptosis, a phenotype that may be addressed by combining MDM2 degraders with BH3 mimetics such as venetoclax, with which KT-253 has already been shown to synergize (27). Additionally, combinations targeting other MCC-relevant pathways, including MDM4, LSD1, HDAC, EZH2, and USP7 inhibitors, could further enhance efficacy in the broader tumor context (2, 6, 14, 32, 39, 41–44). Given the picomolar-to-low-nanomolar concentrations at which KT-253 is effective, therapeutic synergy with these agents may be achievable at doses that minimize overlapping toxicity.

In summary, targeted degradation of MDM2 produces more durable pathway engagement than MDM2 inhibition in WT TP53 cancers. By eliminating MDM2 protein, degraders collapse the p53/MDM2 negative feedback loop and enable p53 transcriptional activation after transient exposure. Quantitative proteomics and mathematical modeling converge to show that this durability reflects fundamental differences in feedback control rather than simple differences in potency. In vivo, these properties translate into deep and sustained tumor regressions, while acquired resistance arises from loss of p53 transcriptional competence rather than escape from MDM2 degradation itself, a hallmark of true on-pathway pressure rather than off-target escape. Together, these findings establish feedback architecture as a critical determinant of therapeutic response and position targeted protein degradation as a rational strategy for overcoming intrinsic limitations of inhibition in feedback-regulated oncogenic pathways.

## Methods

### Sex as a biological variable.

In our study, female NSG mice were used for PDX and CDX studies, which allows randomization of mice based on tumor volume prior to treatment start and avoids singly housing male mice. Since the tumors implanted were studied, it is unlikely that the sex of the mice would contribute to the results observed.

### Cell lines.

Established MCC cell lines MKL-1, WaGa, MS-1, and UISO were gifts from Masahiro Shuda (University of Pittsburgh, Pittsburgh, Pennsylvania, USA), Jürgen Becker (University of Medicine at Essen, Essen, Germany), and Roland Houben (University Hospital Würzburg, Würzburg, Germany) as mentioned previously (20). MKL-1 (RRID:CVCL_2600, male, 26Y), WaGa (RRID:CVCL_E998, male, 67Y), PeTa (RRID:CVCL_LC73, male, 65Y), MS-1 (RRID:CVCL_E995, female, 59Y), and UISO (RRID:CVCL_E996, female, 46Y) cell lines were cultured in RPMI medium (Gibco) supplemented with 10% FBS, 1% penicillin and streptomycin, and 1% GlutaMAX (Gibco). MCC-301, MCC-336 (obtained from PDX tumors expanded in mice, as described in ref. 39; a gift from Catherine J. Wu, Dana-Farber Cancer Institute), and PDX-derived cell lines were cultured in NeuroCult NS-A medium (StemCell Technologies, 05750) with 10% proliferation supplement (StemCell Technologies, 05753), 1% penicillin and streptomycin, EGF, FGF (StemCell Technologies, 78003.1 and 78006.1; 10 μL of 100 μg/mL stock solutions in 50 mL medium), and 0.02% Heparin (StemCell Technologies, 07980). This medium is referred to as NSA-complete (NSA-C) medium in Supplemental Methods. Accutase (StemCell Technologies, for suspension cell lines that clump) or trypsin (only for the UISO cell line) was used as a dissociating agent to obtain single-cell suspensions for cell counting. Cells were routinely tested for mycoplasma contamination using an e-Myco Mycoplasma PCR detection kit (Bulldog-Bio, 25233).

### Chemicals/compounds.

KTX-049 and KT-253 predissolved solutions were provided by Kymera Therapeutics. DS-3032 was obtained from MedChemExpress and was provided by Kymera Therapeutics as a predissolved solution in DMSO or in powder form (HY-101266). AMG232 was obtained from VWR (75837-714) and dissolved in DMSO. MG132 and Bortezomib were obtained from SelleckChem (S2619 and S1013) and dissolved in DMSO.

### Viability assays.

Viable trypan blue cell counts were determined using the Luna automated cell counter (Logos Biosystems). One thousand live cells were seeded per well of a 96-well plate in cell culture media, and DS-3032, AMG232, or KTX-049 was added at the final desired concentration. The cells were treated for the desired time followed by the addition of CellTiter-Glo 2.0 reagent (Promega, G9242) and incubation according to the manufacturer’s protocol. Luminescence was measured using a FLUOstar Omega plate reader (BMG Labtech). Viability was calculated relative to DMSO-treated samples and plotted on a graph using GraphPad Prism (RRID:SCR_002798) version 10.4.

### Western blotting.

Cells were collected by centrifugation, washed with cold PBS supplemented with protease (EMD Millipore, 539-131) and phosphatase inhibitors (EMD Millipore, 524624), and processed immediately or stored at –80°C until processing. Cell pellets were lysed in EBC buffer (50 mM Tris-HCl, pH 8.0, 200 mM NaCl, 0.5% NP-40, and 0.5 mM EDTA) supplemented with protease and phosphatase inhibitors and 2-mercaptoethanol (Bio-Rad, 1610710). Cell lysates were clarified by centrifugation at 20,000*g* for 20 min at 4°C, and protein content was measured using the Bradford assay (Bio-Rad, 5000006). Protein content was normalized, 6× reducing buffer (Boston Bioproducts, BP-111R) was added to the lysates to a final 1× concentration, and the lysates were boiled at 95°C for 5 min. Lysates were run in a 4%–20% SDS-PAGE gel and transferred onto a 0.2 μm nitrocellulose membrane (Bio-Rad, 1620112). The membranes were blocked with 5% milk and 0.01% sodium azide in TBST and incubated overnight with primary antibodies diluted in blocking buffer. The next day, membranes were washed 3 times with TBST buffer followed by incubation with appropriate HRP secondary antibody diluted in 1% milk in TBST for 1 h, washed 3 times with TBST buffer, and imaged using the Syngene G-Box.

Western blot band quantification used for mathematical modeling was performed using the GeneTools software from Syngene (RRID:SCR_022505). Raw and normalized band quantifications are available in the Supporting Data Values file.

### RT-qPCR.

MKL-1 and WaGa cells were treated with DMSO, KTX-049, or DS-3032. Cells were collected by centrifugation at the desired time point and resuspended in TRIzol reagent (Life Technologies, 15596026) followed by incubation at room temperature for 5 min. The homogenized samples were stored at –80°C until processing. RNA was isolated from the samples following the RNA isolation protocol, and cDNA was synthesized using a cDNA synthesis kit (Life Technologies, 4368814) following the manufacturer’s instructions. qPCR was performed using the cDNA-specific qPCR primers listed in Table 12 and Brilliant III Ultra-Fast qPCR Master Mix (Agilent, 600882) on an AriaMx machine (Agilent). Ct values were used to calculate the relative fold changes in expression using the ΔΔCt method.

### Sample preparation for TMT-based proteome profiling.

MKL-1 and WaGa cells were treated with DMSO, KTX-049, or DS-3032 in triplicate for the desired periods, and the cells were collected by centrifugation. Cell pellets were lysed using lysis buffer containing 8 M urea, 200 mM EPPS (pH 8.5), protease inhibitors (Thermo Fisher Scientific, Pierce A32953), and syringe lysis. Protein lysates were clarified by centrifugation, and protein concentrations were estimated using the Pierce BCA Protein assay (catalog 23227). 100 μg protein per sample was reduced using 5 mM neutralized TCEP (Pierce, 77720) for 15 min, alkylated using 10 mM iodoacetamide (Thermo Fisher Scientific, Pierce A39271) for 30 min in the dark, quenched using 5 mM DTT for 15 min, and subjected to methanol chloroform precipitation. Samples were then profiled using TMT-based proteome profiling.

### DIA-based proteomics analysis of PDX samples.

PDX tumor samples were harvested from mice treated with 10 mg/kg KT-253 or vehicle for 24 h (3 mice per group). Half of each tumor was collected and lysed in 500 μL ice-cold lysis buffer (Pierce RIPA lysis buffer, 1× HALT protease/phosphatase inhibitor, and 1× Pierce universal nuclease) by homogenization in a BeatBox (PreOmics) set to high power for 20 min. The lysates were centrifuged at 13,000 RCF for 10 min at 4°C to remove insoluble material. Protein concentrations were determined using a Pierce Rapid Gold BCA kit. Protein lysates for proteomics were processed for mass spectrometry analysis using an Opentrons Flex liquid handling robot adapted to perform the single-pot solid-phase sample preparation method (41). In quadruplicate, 50 μg of total protein from each lysate was reduced, alkylated, and digested into peptides using trypsin and LysC endopeptidase. Tryptic peptides were desalted with Affinisep C18 StageTips, resuspended, separated by reverse-phase nano–liquid chromatography with an IonOpticks Aurora Ultimate XT 25 cm × 75 μm C18 UHPLC column, and analyzed online using a Thermo Fisher Scientific Orbitrap Astral mass spectrometer operated in narrow-window DIA mode (42).

Raw data were processed using DIA-NN (RRID:SCR_022865, version 1.8.1) (43) and searched against a comprehensive SwissProt sequence database (released January 15, 2019) in a library-free setting. Downstream bioinformatics analysis was performed using the R statistical programming language. Evidence was filtered for precursors present in at least 60% of all samples with a minimum of 2 distinct amino acid sequences detected per protein. Protein quantification was then performed at the gene level using the MaxLFQ algorithm (44). An unpaired limma test (45) was used to compare KT-253 and vehicle-treated samples. Statistical results were only reported if a protein was quantified in at least 50% of all replicate samples in both test groups.

To determine significance, a weighted significance cutoff was applied that factors in both statistical significance and fold change. Proteins were deemed statistically significant on either tail when they fell outside of the cutoff function defined as follows: f(x) = y0 + 1/((x/a)^2 − x0), where x is log_2_ fold change, y is –log_10_(*P* value), y0 = –log_10_(0.05), x0 = 0.5, and a = 1.55.

### In vivo efficacy studies.

7- to 9-week-old female NSG mice (The Jackson Laboratory) were subcutaneously implanted with 1 of the 3 xenograft models in the right flank: PDX 48396 tumor fragments (*N =* 55 mice, 6/group), PDX 96712 tumor fragments (*N =* 50 mice, 6/group), WaGa CDX, and 10 million cells in PBS containing 50% matrigel (*N =* 55 mice, 8/group).

For all efficacy studies, tumor volumes and BWs were recorded twice weekly. Mice that had tumor growth were randomized on a rolling admission basis into 1 of the 6 efficacy treatment groups once tumor volumes reached 74.1–214.2 mm^3^ (PDX 48396), 61.4–268.4 mm^3^ (PDX 96712), and 67.5–196.0 mm^3^ (WaGa CDX). All dosed animals were maintained and observed and were not excluded until they met the endpoint. Mice that met the endpoints, such as excessive tumor volume (>2,000 mm^3^) or morbidity, were euthanized. The KT-253 solution was provided by Kymera and administered intravenously. DS-3032 was formulated as a suspension in 5% methylcellulose (400 cP) in water and administered orally.

For a subset of mice from each efficacy study, tumors were harvested at the endpoint. One-third was fixed in 10% formalin, one-third was flash frozen, and the other third was collected fresh into PBS and shipped to the lab for further analysis. Percent tumor growth inhibition (TGI) was calculated using the following formula: [–100 × ((VT day X – VT day 0)/(VC day X – VC day 0))] + 100, where VT is the mean tumor volume of treated group on day 0 or X (any day after day 0), and VC is the mean tumor volume of control group on day 0 or X. Percent regression was calculated as follows: [100 – ((V day X/V day 0)*100)), where V is mean tumor on day 0 or X.

### Parameter estimation in the mechanistic mathematical model.

Inference was performed using Stan (RRID:SCR_018459, v2.34.1) in the R package rstan (v2.32.6) (46, 47). Specifically, we considered the Western blot data (DMSO condition) shown in Figure 2, A and B. We implemented a Stan model incorporating the ordinary differential equation (ODE) system associated with the p53/MDM2 negative feedback loop (Supplemental Figure 6) and used the DMSO dataset to infer the posterior distributions of the ODE model parameters. These posterior distributions were used as priors for DS-3032 and KTX-049. These prior distributions are defined as follows. Let *ϴ* = (*α_P_*, *γ_P_*, *κ*, *K_1_*, *α_M_*, *α_M_*, *K_2_*, *γ_M_*, *n*) with independent priors *ϴ*_i_~𝓝_[0,∞)_ (*μ_i_*, *σ_i_*^2^), *i* = 1,..., 9, where 𝓝_[0,∞)_ denotes a normal distribution truncated to the non-negative real line. For the MKL1 cell line (Figure 2A), we have *μ* = (1.54, 1.56, 1.47, 1.19, 1.66, 2.48, 1.35, 1.78, 4.07) and *σ* = (1.01, 0.82, 0.83, 0.77, 1.07, 1.54, 0.78, 0.75, 2.39), and for the WaGa cell line (Figure 2B), we have *μ* = (2.92, 1.32, 1.28, 1.33, 2.62, 2.64, 1.26, 1.50, 3.91) and *σ* = (1.24, 0.73, 0.74, 0.79, 1.43, 1.55, 0.75, 0.61, 2.39). New parameters related to the interactions of DS-3032 and KTX-049 with MDM2 (Supplemental Figure 6, B and C) were assigned broad normal priors to allow flexibility in the estimation (48). We used the experimental data for DS-3032 and KTX-049 (Figure 2, A and B) to estimate the model parameters specific to these cases. In Figure 3, D and E, we present the estimated trajectories, showing the mean and 95% credible interval, and present the mathematical conditions under which KTX-049 is more potent than DS-3032 along with their numerical evaluation, using the mean values of the estimated posterior parameter distributions. For parameter estimation, we assumed that KTX-049 and DS-3032 concentrations remained approximately constant throughout the experiment (24 h). We repeated this analysis with 2 additional experiments (Supplemental Figure 3 and Figure 2, C and D, for experimental data; Supplemental Figure 7 for computational results). For the first experiment (Supplemental Figure 3), for the MKL1 cell line, we used *μ* = (3.33, 1.26, 1.15, 1.50, 3.18, 3.07, 1.19, 0.88, 3.96) and *σ* = (1.41, 0.73, 0.65, 0.77, 1.61, 1.62, 0.76, 0.37, 2.49), and for the WaGa cell line, we used *μ* = (3.19, 1.28, 1.17, 1.45, 2.95, 2.77, 1.22, 1.23, 3.89) and *σ* = (1.28, 0.71, 0.69, 0.79, 1.51, 1.62, 0.75, 0.52, 2.26). For the second experiment (Figure 2, C and D), for the MKL1 cell line, we used *μ* = (2.16, 1.55, 1.43, 1.24, 1.46, 2.26, 1.47, 1.88, 4.07) and *σ* = (0.97, 0.76, 0.81, 0.79, 0.91, 1.53, 0.76, 0.72, 2.33), and for the WaGa cell line, we used *μ* = (1.95, 1.56, 1.38, 1.26, 1.11, 2.20, 1.55, 1.96, 4.12) and *σ* = (0.96, 0.81, 0.82, 0.81, 0.76, 1.50, 0.74, 0.74, 2.39). Finally, we conducted a sensitivity analysis (Supplemental Figure 7), which demonstrated that a decrease of up to 20% in the initial drug concentrations by the end of the experiment did not notably affect the estimated trajectories. Specifically, this decrease introduced only a small difference compared with the original estimated trajectory, quantified as 8.59% ± 3.34% for MDM2 and 3.68% ± 1.55% for p53, where the values represent the mean percentage difference ± SD.

### Mathematical model data analysis.

Data manipulation and plotting were performed in R (v4.3.2) using tidyverse (v2.0.0) and MATLAB (RRID:SCR_001622, v24.2.0.2773142, R2024b Update 2) (49–51).

### Statistics.

GraphPad Prism was used for statistical analysis, and methods used are denoted in figure and table legends. *P* values of less than 0.05 were considered significant. Tumor volume comparisons (Tables 4, 6, and 8 and Supplemental Figure 15) and in vitro caspase-3/7 and annexin V/PI assays (Figure 4) were analyzed by 2-way ANOVA with Tukey’s multiple-comparison test. Statistical tests used for Figure 5 are denoted in the supplemental materials. Adjusted *P* values in Figure 7 were calculated by fgsea based on an adaptive, multilevel, split Monte-Carlo scheme.

### Study approval.

The Dana-Farber Cancer Institutional Animal Care and Use Committee approved this in vivo study. Appropriate ethical regulations for animal research were followed.

### Data availability.

The data generated in this study are included in the main article and supplemental information. Values for data points in graphs are reported in the Supporting Data Values file. Materials generated in this study are available upon request from the corresponding author. The WES datasets were deposited in the Sequence Read Archive (PRJNA1241785). RNA-seq datasets (GSE292992) from this study were deposited in Gene Expression Omnibus database. Raw data for TMT profiling in MKL-1 and WaGa cells were deposited in PRIDE (RRID:SCR_003411) under accession number PXD061890. The raw data for the DIA analysis of PDX tumor samples were deposited in the PRIDE database under accession number PXD065666. This article does not report any original code.

## Author contributions

Conceptualization: VA, YC, and JAD. Data curation, formal analysis, and investigation: VA, SB, LV, AT, BKE, CLH, JSR, JS, and AK. Methodology: VA, SB, LV, YCC, AT, BKE, JAP, CLH, JSR, JS, AK, PCG, RK, SBB, KS, YL, ADC, FM, YC, JD, and JAD. Writing, first draft: VA and JAD. Writing, review and editing: VA, SB, LV, YCC, AT, BKE, JAP, CLH, JSR, JS, AK, PCG, RK, SBB, KS, YL, ADC, FM, YC, JD, and JAD.

## Conflict of interest

ADC received research funding from Bayer and consulted for Karyo Verse. FM is a cofounder of and has equity in Harbinger Health and Zephyr AI and serves as a consultant for both companies. FM is on the board of directors of Recursion Pharmaceuticals. JAD received research support from Kymera Therapeutics, Inc., and consulted for Mariana Oncology, Inc. CLH, RK, SBB, KS, YC, and JD are employees of Kymera Therapeutics, Inc.

## Funding support

This work is the result of NIH funding, in whole or in part, and is subject to the NIH Public Access Policy. Through acceptance of this federal funding, the NIH has been given a right to make the work publicly available in PubMed Central.

- US Public Health Service grants R35CA232128 and P01CA203655 to JAD.
- The Bridge Project, a partnership between the Koch Institute for Integrative Cancer Research at MIT and Dana-Farber/Harvard Cancer Center (to JAD).
- Kymera Therapeutics, Inc., research funding to JAD.
- Dana-Farber Cancer Institute Center for Cancer Evolution (to FM and SB).

## Supplementary Material

Supplemental data

Unedited blot and gel images

Supporting data values

## Figures and Tables

**Table 12:**
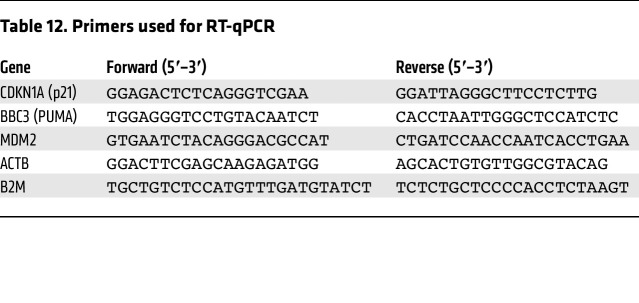
Primers used for RT-qPCR

**Table 7:**
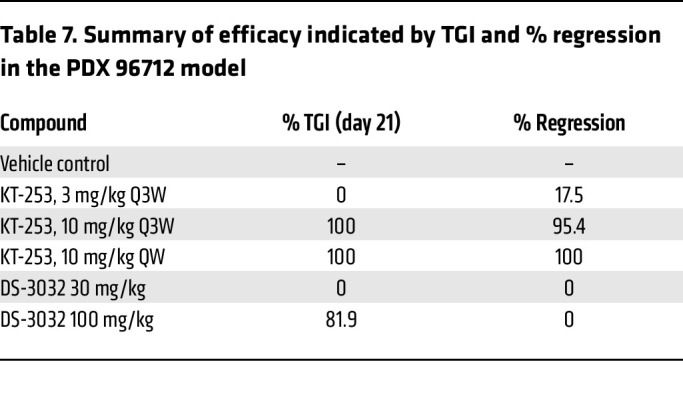
Summary of efficacy indicated by TGI and % regression in the PDX 96712 model

**Table 6:**
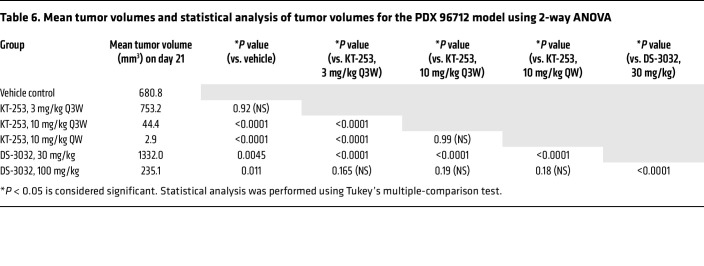
Mean tumor volumes and statistical analysis of tumor volumes for the PDX 96712 model using 2-way ANOVA

**Table 5:**
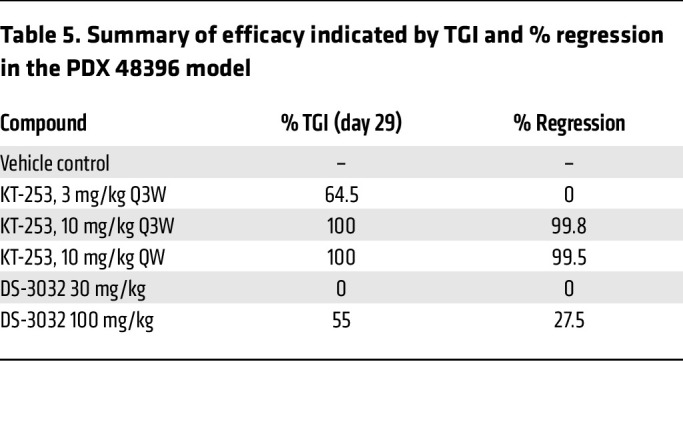
Summary of efficacy indicated by TGI and % regression in the PDX 48396 model

**Table 4:**
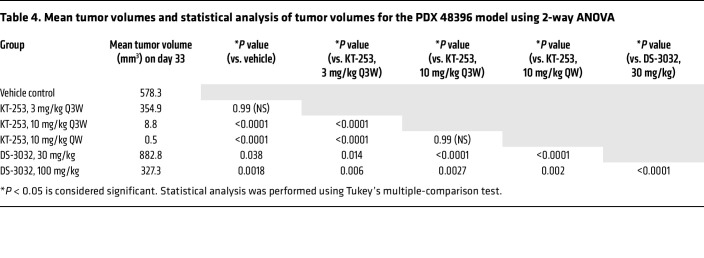
Mean tumor volumes and statistical analysis of tumor volumes for the PDX 48396 model using 2-way ANOVA

**Table 3:**
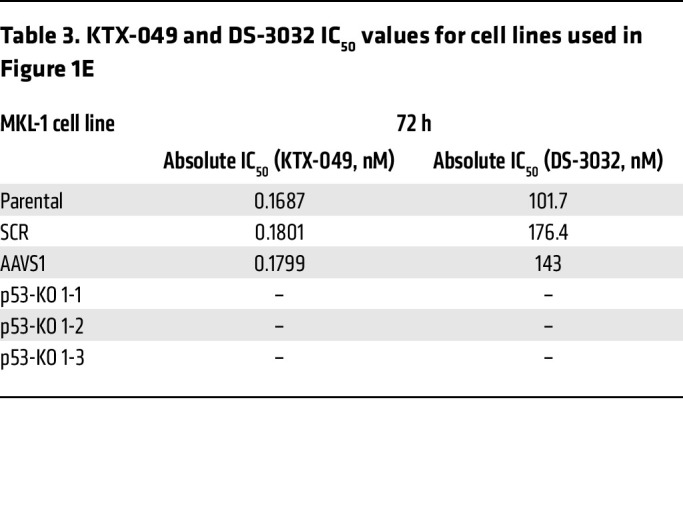
KTX-049 and DS-3032 IC_50_ values for cell lines used in Figure 1E

**Table 2:**
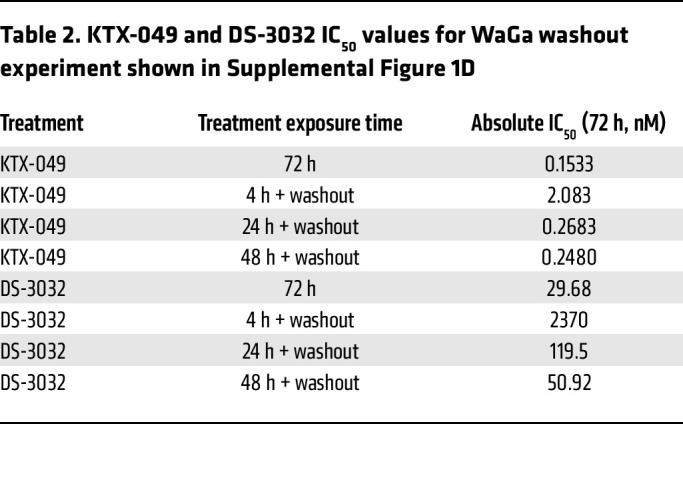
KTX-049 and DS-3032 IC_50_ values for WaGa washout experiment shown in Supplemental Figure 1D

**Table 1:**
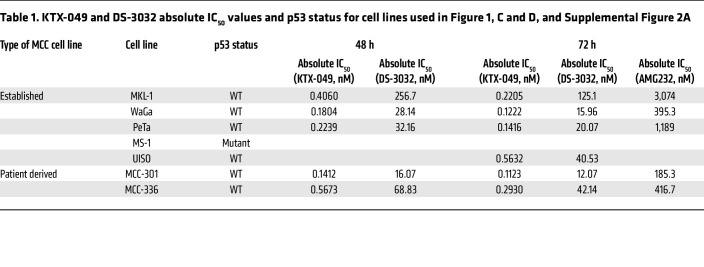
KTX-049 and DS-3032 absolute IC_50_ values and p53 status for cell lines used in Figure 1, C and D, and Supplemental Figure 2A

**Table 11:**
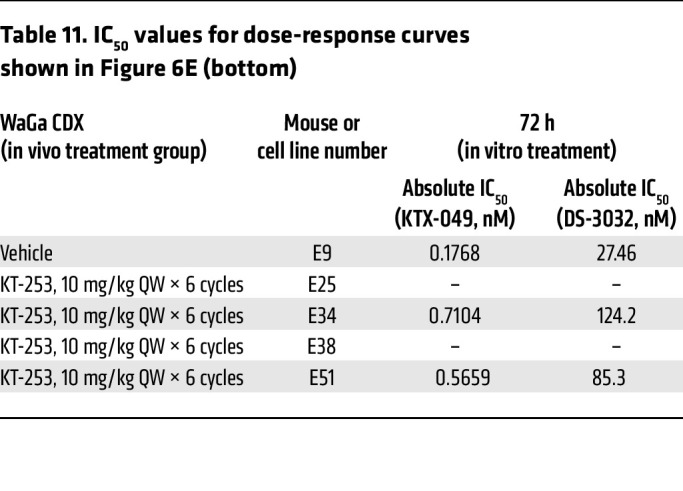
IC_50_ values for dose-response curves shown in Figure 6E (bottom)

**Table 10:**
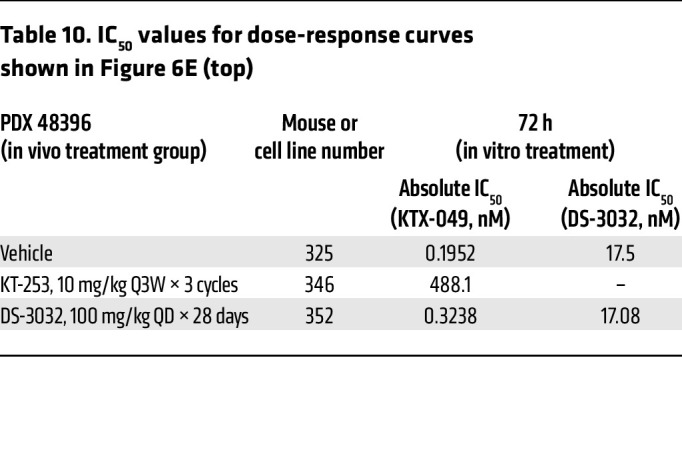
IC_50_ values for dose-response curves shown in Figure 6E (top)

**Table 9:**
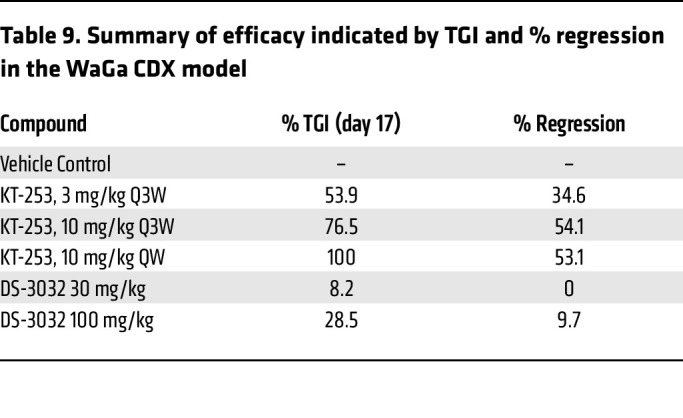
Summary of efficacy indicated by TGI and % regression in the WaGa CDX model

**Table 8:**
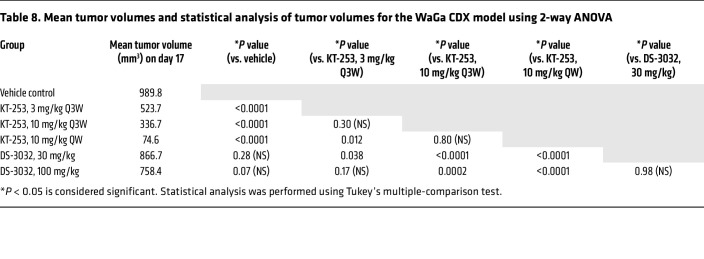
Mean tumor volumes and statistical analysis of tumor volumes for the WaGa CDX model using 2-way ANOVA

## Version 1 07/01/2026

Electronic publication
