# Supplementary material for: Targeted degradation of MDM2 overcomes feedback regulation of p53 signaling in Merkel cell carcinoma models
Source: J Clin Invest. 2026 Jul 1;136(13):e199049. doi: 10.1172/JCI199049 (PMC13318125; doi:10.1172/JCI199049)
Supplement: Supplemental data [file jci-136-199049-s256.pdf]

Supplemental Figures

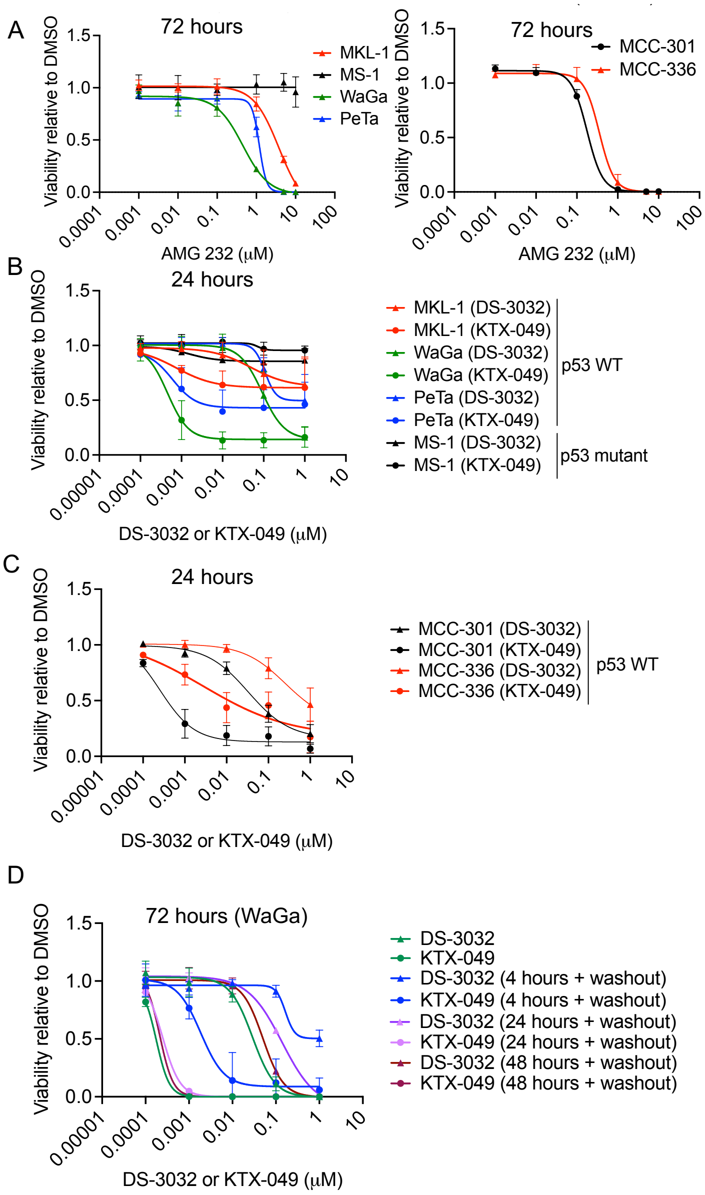

Supplemental Figure 1

MCCP MKL-1, WaGa, PeTa and MS-1 cell lines (A, B), MCCP PDCL cell lines MCC-301 and MCC-336 (A, C) were treated with different concentrations of AMG 232 in (A), KTX-049 or DS-3032 in (B, C). The Cell Titer Glo assay readout was used to determine cell viability after 24 h or 72 h as indicated. (D) WaGa cells were treated with KTX-049 or DS-3032 for 4, 24, or 48 h, washed out or treated for 72 h, and cell viability was measured using the Cell Titer Glo assay. N=3. Error bars indicate the standard deviation.

A

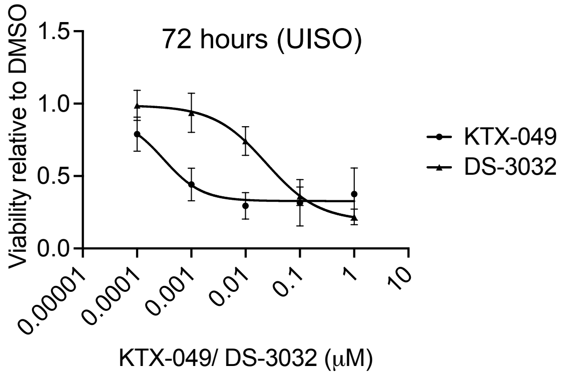

B

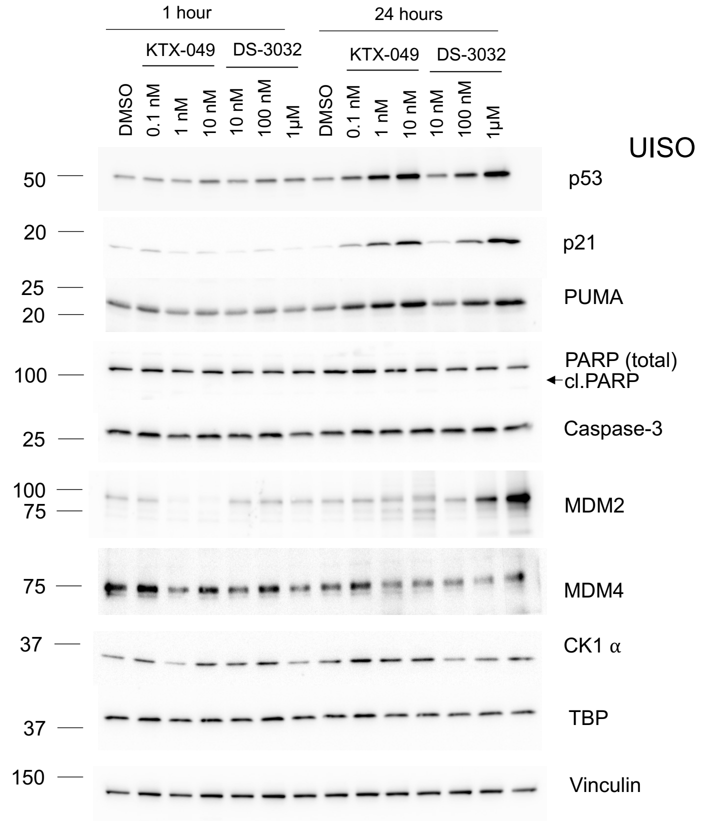

Supplemental Figure 2

(A) UISO cells were treated with different concentrations of KTX-049 or DS-3032 and the Cell Titer Glo assay readout was used to determine cell viability at 72 h. N=3. Error bars indicate the standard deviation. (B) MCCN UISO cells were treated with DMSO, 1 nM KTX-049 or 100 nM DS-3032 for 1 or 24 h, and the p53 response was analyzed using WB for the indicated proteins. TBP and Vinculin were used as loading controls. N=2, WB from one repeat is shown. Multiple gels were run with equal volumes of the same lysates to analyze the indicated proteins. Membranes for MDM2 and p21 were stripped and re-probed to analyze the levels of Vinculin and PUMA respectively.

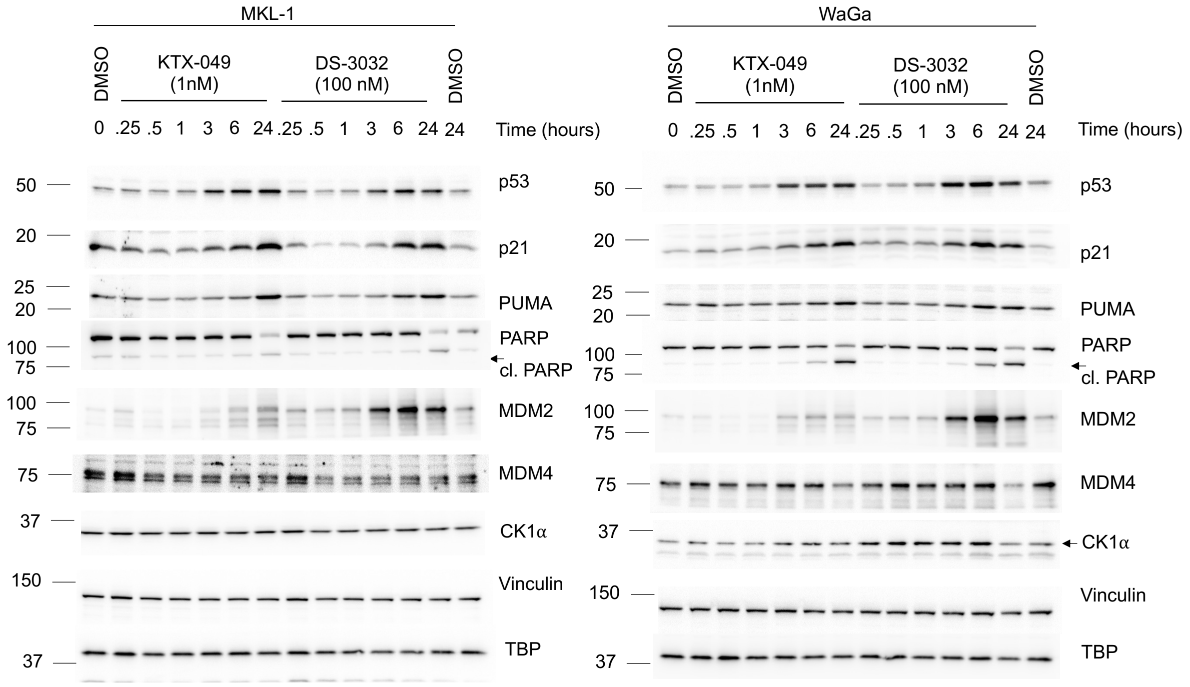

Supplemental Figure 3

The second biological repeat for WB is shown in Fig. 2A and B for MKL-1 and WaGa cells. Multiple gels were run with equal volumes of the same lysate to analyze the indicated proteins. As in Fig. 2A and 2B, the MDM2 and p21 membranes were stripped and re-probed to analyze the levels of Vinculin and PUMA, respectively.

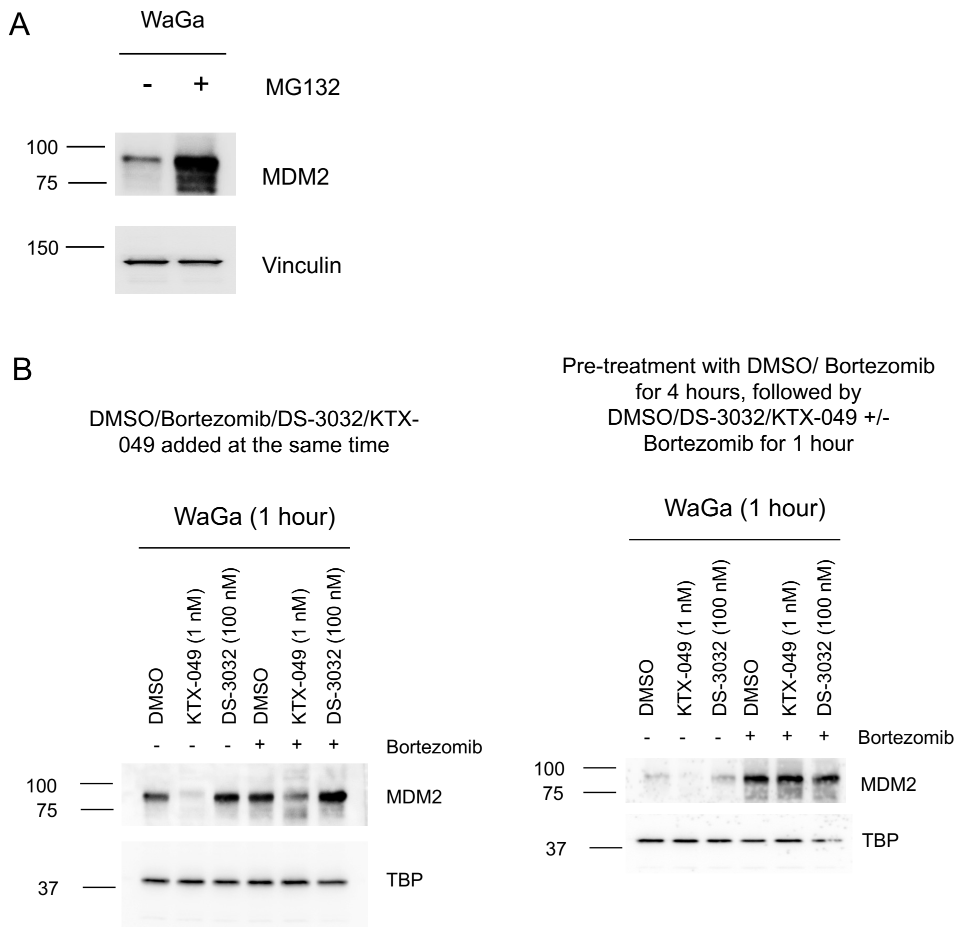

Supplemental Figure 4

(A) WB showing MDM2 migration patterns after treatment of WaGa cells with 50 μg/mL MG132 for 4h. Vinculin was used as a loading control (B) WB showing MDM2 migration patterns when cells were treated with KTX-049 or DS-3032 +/- Bortezomib (left) or when cells were treated with Bortezomib for 4 h followed by treatment with KTX-049 or DS-3032 +/- Bortezomib for 1h.

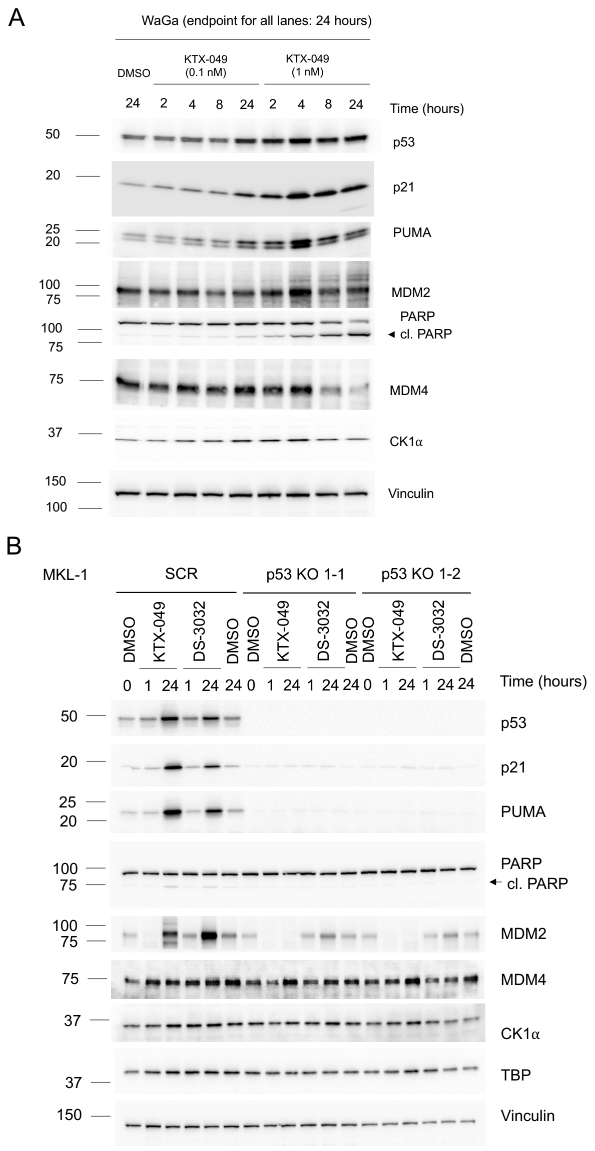

Supplemental Figure 5

(A) WaGa cells were treated with DMSO, 0.1 nM KTX-049 or 1 nM KTX-049 for 2, 4, and 8 h followed by washout or for 24 h followed by collection at 24 h and WB analysis of the indicated proteins. Vinculin was used as a loading control. Multiple gels were run with equal volumes of the same lysates to analyze the indicated proteins. Membranes for MDM2 and p21 were stripped and re-probed to analyze the levels of Vinculin and PUMA respectively. N=1. (B) MKL-1 SCR or p53 KO 1-1 and 1-2 cell lines were treated with

DMSO, 1 nM KTX-049 or 100 nM DS-3032 for 1 or 24 h, and the p53 response was analyzed using WB analysis for the indicated proteins. TBP and Vinculin were used as loading controls. N=2, WB from one repeat is shown. Multiple gels were run with equal volume of the same lysates to analyze the indicated proteins. Membranes for MDM2 and p21

A p53-MDM2 feedback loop circuit model

Chemical reactions

$$\emptyset \underset{\gamma_P}{\overset{\alpha_P}{\rightleftharpoons}} \mathrm{P}, \quad \mathrm{P} \xrightarrow{k_P(M)} \emptyset,$$

$$\emptyset \underset{\gamma_M}{\overset{\alpha_M}{\rightleftharpoons}} \mathrm{M}, \quad \emptyset \xrightarrow{k_M(P)} \mathrm{M},$$

$$\text{with } k_P(M) = \kappa \frac{M}{K_1 + P}$$

$$\text{and } k_M(P) = \bar{\alpha}_M \frac{P^n}{K_2^n + P^n}.$$

ODEs

$$\dot{P} = \alpha_P - (\gamma_P + \kappa \frac{M}{K_1 + P})P$$

$$\dot{M} = \alpha_M + \bar{\alpha}_M \frac{P^n}{K_2^n + P^n} - \gamma_M M$$

B KTX-049 degrader model

Chemical reactions

$$\mathrm{M} + \mathrm{D} \xrightarrow{\gamma} \mathrm{D}$$

ODEs

$$\dot{M} = -(\gamma D)M$$

C DS-3032 inhibitor model

Chemical reactions

$$\mathrm{M} + \mathrm{I} \underset{d}{\overset{a}{\rightleftharpoons}} \mathrm{C},$$

$$\mathrm{C} \xrightarrow{\delta} \emptyset$$

ODEs

$$\dot{M} = -(aI)M + dC$$

$$\dot{C} = aIM - (d + \delta)C$$

Supplemental Figure 6

Chemical reactions and Ordinary Differential Equations (ODE's) associated with the p53-MDM2 feedback loop circuit model (A), KTX-049 degrader model (B), and the DS-3032 inhibitor model (C). In (A), the promotion of p53 degradation by MDM2 was modeled by a Michaelis-Menten function (1) with constant K_1(A.U.), and the induction of MDM2 transcription by p53 as a Hill function (1) with constant K_2(A.U.) and coefficient *n* (2). Furthermore, parameters α_P (A.U.), γ_P (h^{-1}), α_M (A.U./h), γ_P (h^{-1}), κ (h^{-1}), $\bar{\alpha}_M$ (A.U./h) denote the reaction rate constants associated with the reactions listed in the panel, P represents p53, and M represents MDM2. In (B), D represents the KTX-049 degrader, and γ ($\mathrm{nM}^{-1}\mathrm{h}^{-1}$) represents the rate of MDM2 degradation by KTX-049. In (C), I denotes the DS-3032 inhibitor and C denotes the complex between MDM2 and DS-3032, DS-3032: MDM2, which cannot interact with p53. Furthermore, a ($\mathrm{nM}^{-1}\mathrm{h}^{-1}$) and d ($\mathrm{h}^{-1}$) represent the rate of binding and unbinding of MDM2 and DS-3032, respectively, and δ (h^{-1}) represents the

decay rate of C, which accounts for the degradation and dilution due to cell growth. In all panels, for species X, we use italics, X, to denote concentration.

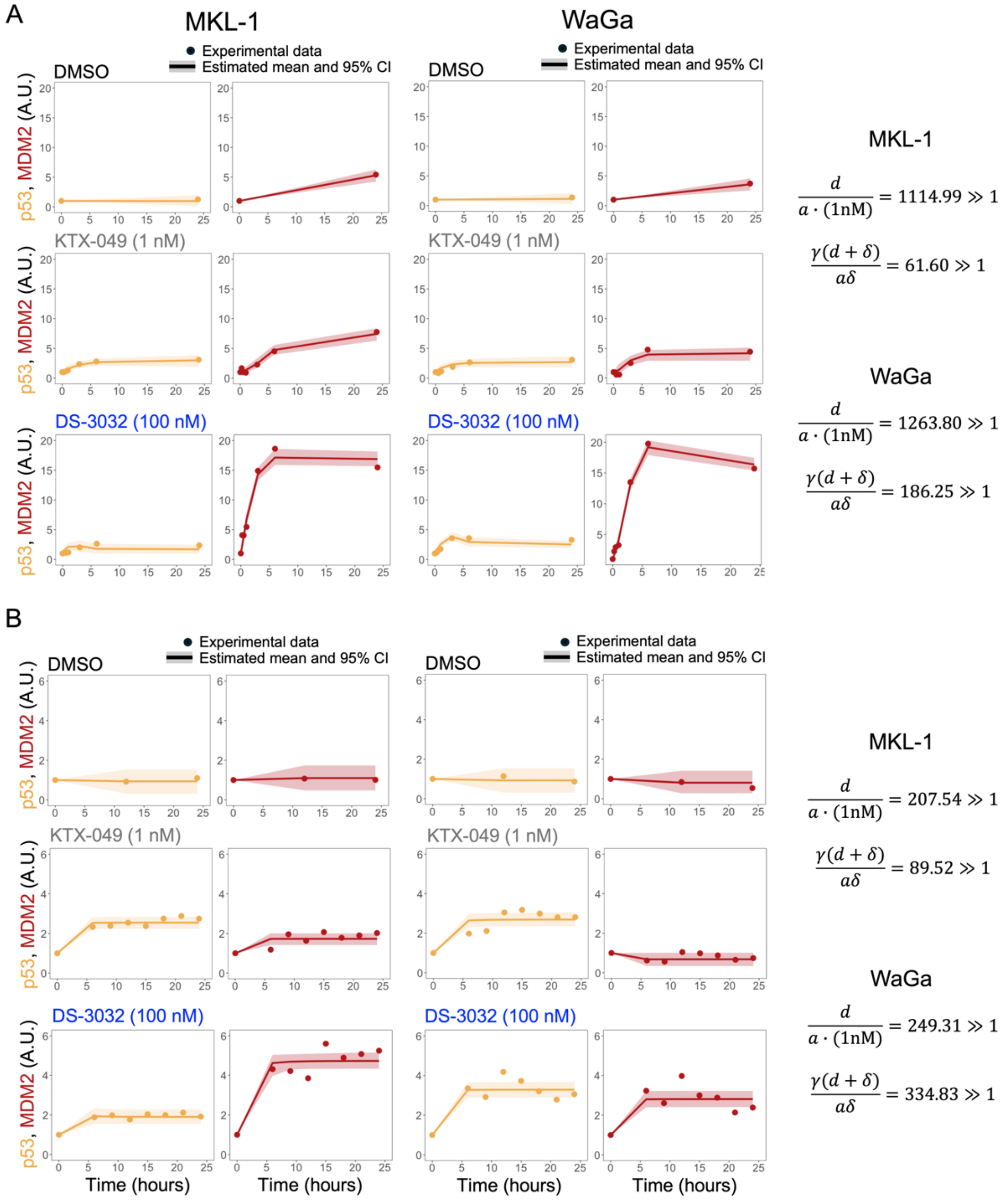

Supplemental Figure 7

(A and B) Observed experimental data (dots) and estimated trajectories (solid lines and shaded areas representing the mean and the 95% credible interval (CI), respectively) of p53 and MDM2 in DMSO, 1 nM KTX-049 or 100 nM DS-3032 in MCCP MKL-1and WaGa cell lines. The experimental data used for the estimation in panel (A) corresponds to those

presented in Supplemental Fig. 3, whereas the data used for the estimation in panel (B) correspond to those presented in Fig. 2C and 2D. The validation of the conditions under which KTX-049 was more potent than DS-3032 is reported on the right hand-side of each panel. More precisely, in (A), we have $a = 0.0053\ \mathrm{nM^{-1}h^{-1}}$, $d = 5.85\ \mathrm{h^{-1}}$, $\delta = 4.16\ \mathrm{h^{-1}}$, $\gamma = 0.16\ \mathrm{nM^{-1}h^{-1}}$ for MKL-1 and $a = 0.0046\ \mathrm{nM^{-1}h^{-1}}$, $d = 5.79\ \mathrm{h^{-1}}$, $\delta = 4.07\ \mathrm{h^{-1}}$, $\gamma = 0.43\ \mathrm{nM^{-1}h^{-1}}$ for WaGa. In (B), we have $a = 0.028\ \mathrm{nM^{-1}h^{-1}}$, $d = 5.81\ \mathrm{h^{-1}}$, $\delta = 3.38\ \mathrm{h^{-1}}$, $\gamma = 1.25\ \mathrm{nM^{-1}h^{-1}}$ for MKL-1 and $a = 0.024\ \mathrm{nM^{-1}h^{-1}}$, $d = 5.92\ \mathrm{h^{-1}}$, $\delta = 3.50\ \mathrm{h^{-1}}$, $\gamma = 3.98\ \mathrm{nM^{-1}h^{-1}}$ for WaGa.

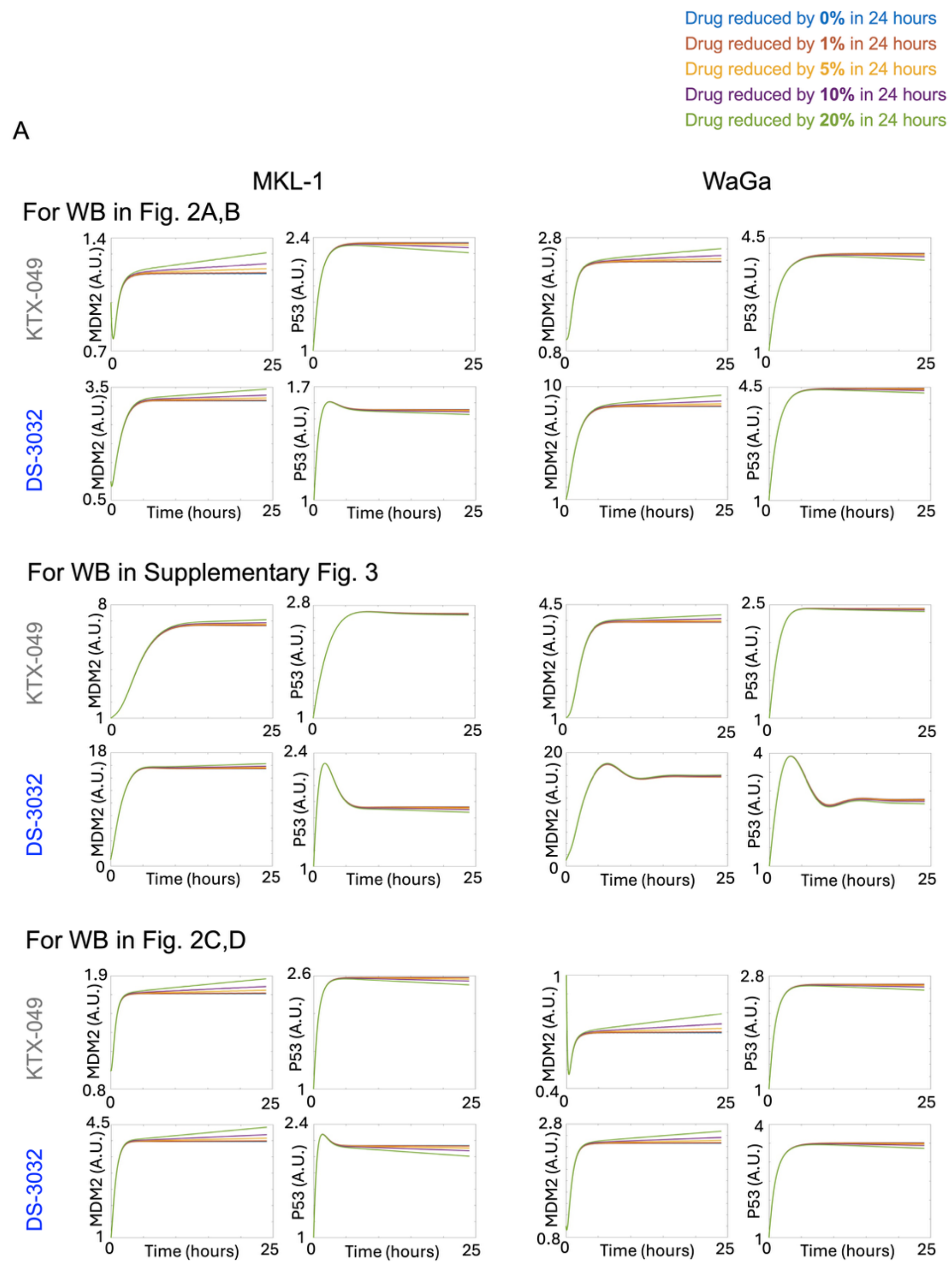

Supplemental Figure 8

Sensitivity analysis to determine the effects of variable drug concentrations on MDM2 and p53 dynamics. Here, deterministic trajectories were obtained by simulating the ODEs in Supplemental Fig. 6 with the mean of the estimated parameters obtained for each condition (KTX-049 and DS-3032), in Figs 2A-D and Supplemental Fig. 3, and cell line (MKL-1, WaGa). For each case, the dynamics of the drug were modeled as a decreasing exponential $X(t) = e^{(-r*t)}$, where $X = KTX-049, DS-3032$ and with r chosen such that $X(t = 24\ hours)/X(t = 0)$ = 1, 0.99, 0.95, 0.9, and 0.8

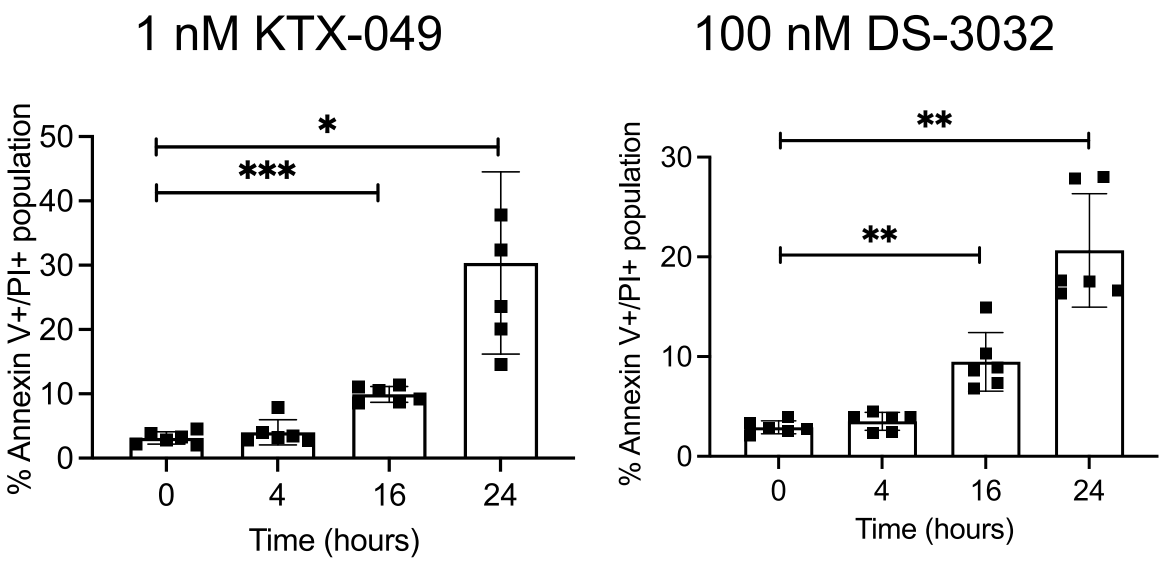

Supplemental Figure 9

MCCP WaGa cells were treated with 1 nM KTX-049 or 100 nM DS-3032 for 4, 16, or 24 h and Annexin V/PI staining was performed. 0h treated samples indicate DMSO controls collected at 24h. Graph indicates mean values from N=6. Error bars indicate standard deviation. Annexin V +, PI + shows late apoptotic or dead cells. 2-Way ANOVA with Tukey's multiple comparison test was performed . Significance for 0 hour treatment as compared to the treatment at 4 hour, 16 hour or 24 hour time-point are shown. **** indicates p-value was <0.0001. *** indicates p-value was <0.001, ** p-value <0.01, * p-value < 0.05. Only significant comparisons as compared to the 0 hour time-point are shown.

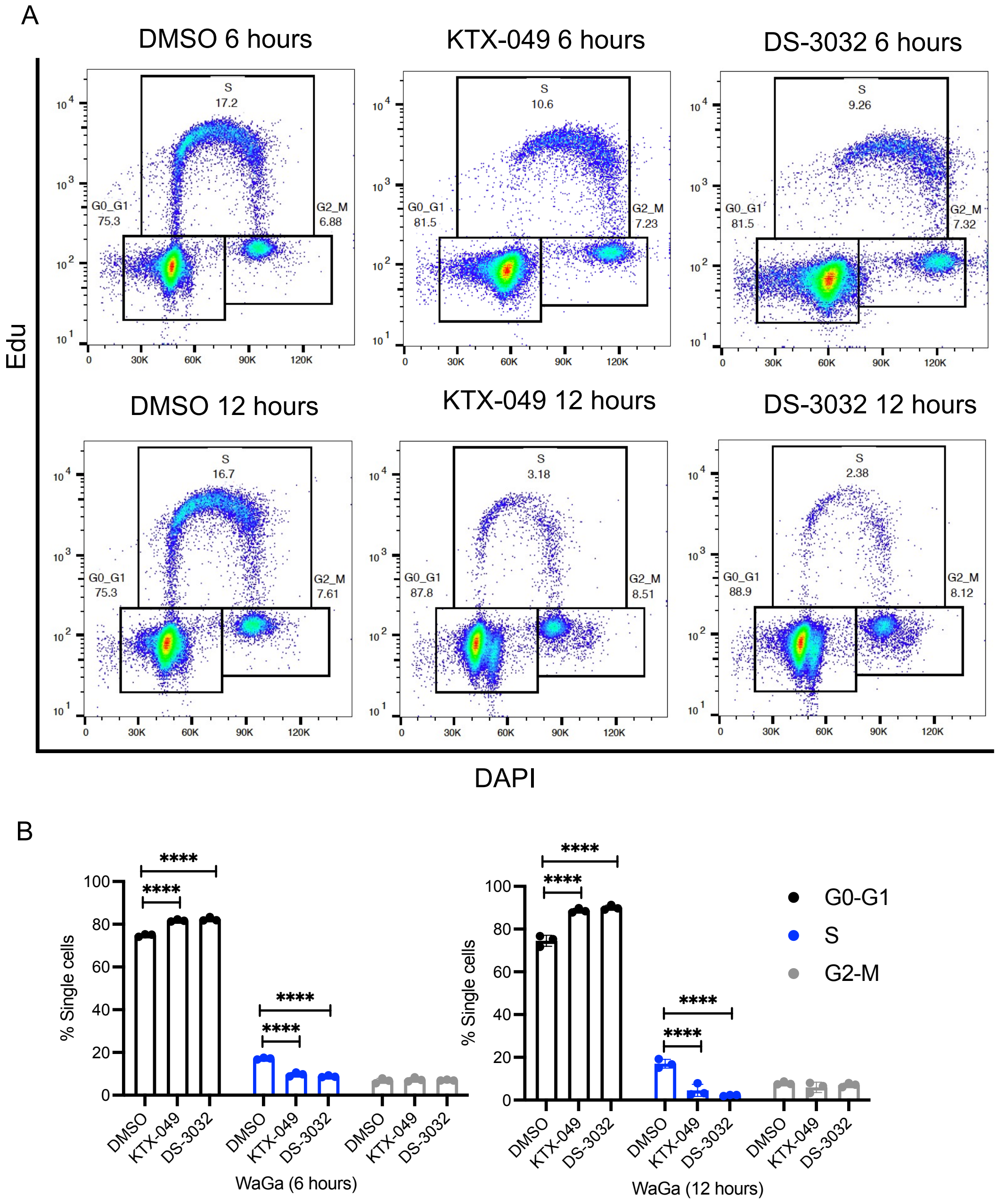

Supplemental Figure 10

MCCP WaGa cells were treated with DMSO, KTX-049 (1 nM) or DS-3032 (100 nM) for 6 h or 12 h, pulsed with EdU for the last hour and cell cycle profiles were analyzed. (A) Cell cycle profiles of cells treated for 6 h or 12 h from one representative experiment. (B) The graph indicates the mean values from N=3. Error bars indicate standard deviation. 2 Way ANOVA with Tukey's multiple comparison statistical tests was performed. **** indicates p-value was <0.0001. Only significant changes as compared to DMSO control are denoted.

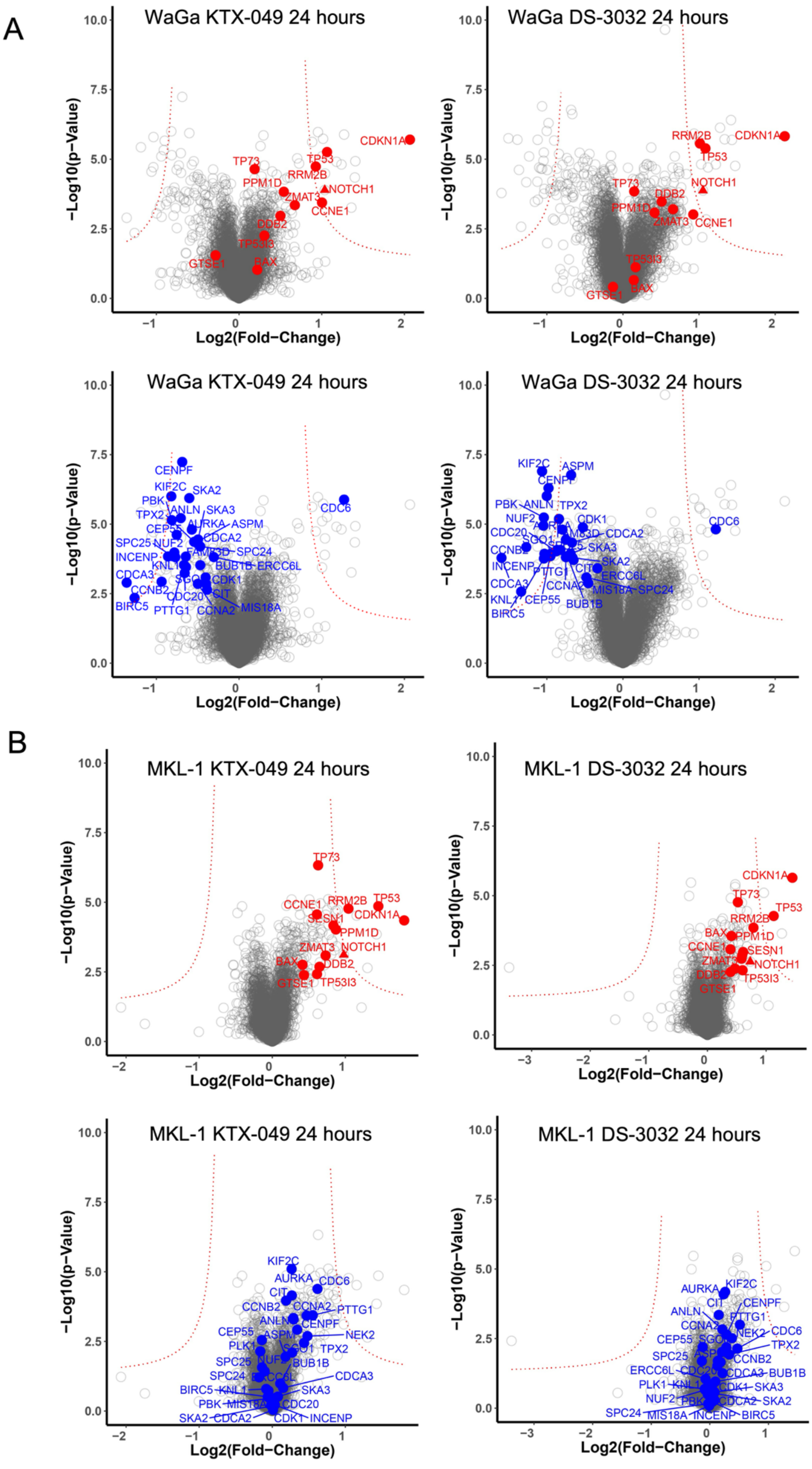
A
WaGa KTX-049 24 hours
WaGa DS-3032 24 hours
WaGa KTX-049 24 hours
WaGa DS-3032 24 hours
B
MKL-1 KTX-049 24 hours
MKL-1 DS-3032 24 hours
MKL-1 KTX-049 24 hours
MKL-1 DS-3032 24 hours
−Log10(p−Value)
Log2(Fold−Change)
CDKN1A
TP53
TP73
RRM2B
PPM1D
NOTCH1
ZMAT3
CCNE1
DDB2
TP53I3
GTSE1
BAX
SESN1
CENPF
KIF2C
PBK
SKA2
ANLN
SKA3
TPX2
AURKA
ASPM
CEP55
CDCA2
SPC25
NUF2
INCENP
KNL1
FAM83D
SPC24
BUB1B
ERCC6L
CDCA3
SGO1
CDK1
CCNB2
CIT
CDC20
BIRC5
PTTG1
CCNA2
MIS18A
CDC6
NEK2
PLK1

Supplemental Figure 11

Volcano plots highlight significantly up-regulated proteins belonging to the KEGG p53 pathway (top, shown in red) or down-regulated proteins belonging to the KEGG mitotic pathway (bottom, shown in blue) with 24 hour KTX-049 or DS-3032 treatment in (A) WaGa cells and (B) MKL-1 cells compared to proteins detected in DMSO treated cells. NOTCH1 is not a KEGG p53 upregulated gene but is a direct p53 target (3) and is shown as a red triangle.

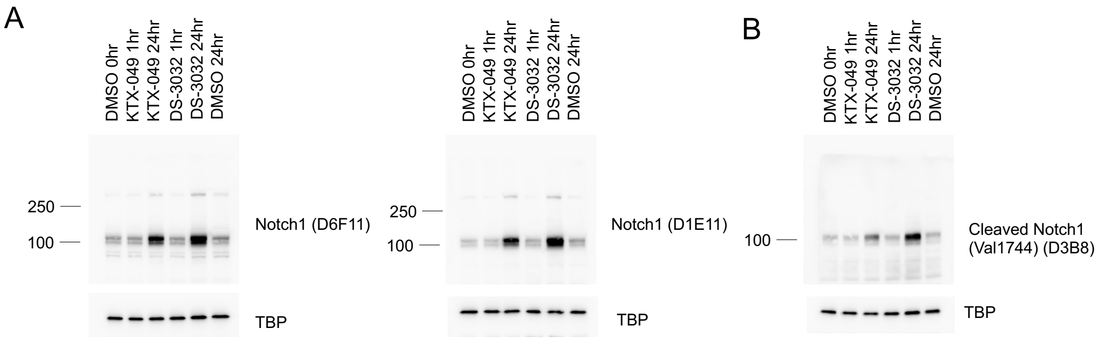

Supplemental Figure 12

MKL-1 cells were treated with DMSO, 1 nM KTX-049 or 100 nM DS-3032 for 24 h and Notch1 levels were detected using three independent antibodies against NOTCH1. Equal volumes of lysates were run three times and the membranes were probed with three different antibodies. TBP was used as a loading control. Antibodies in A detect both the full-length and cleaved forms of Notch1. The antibody in B detected only cleaved Notch1. N=1.

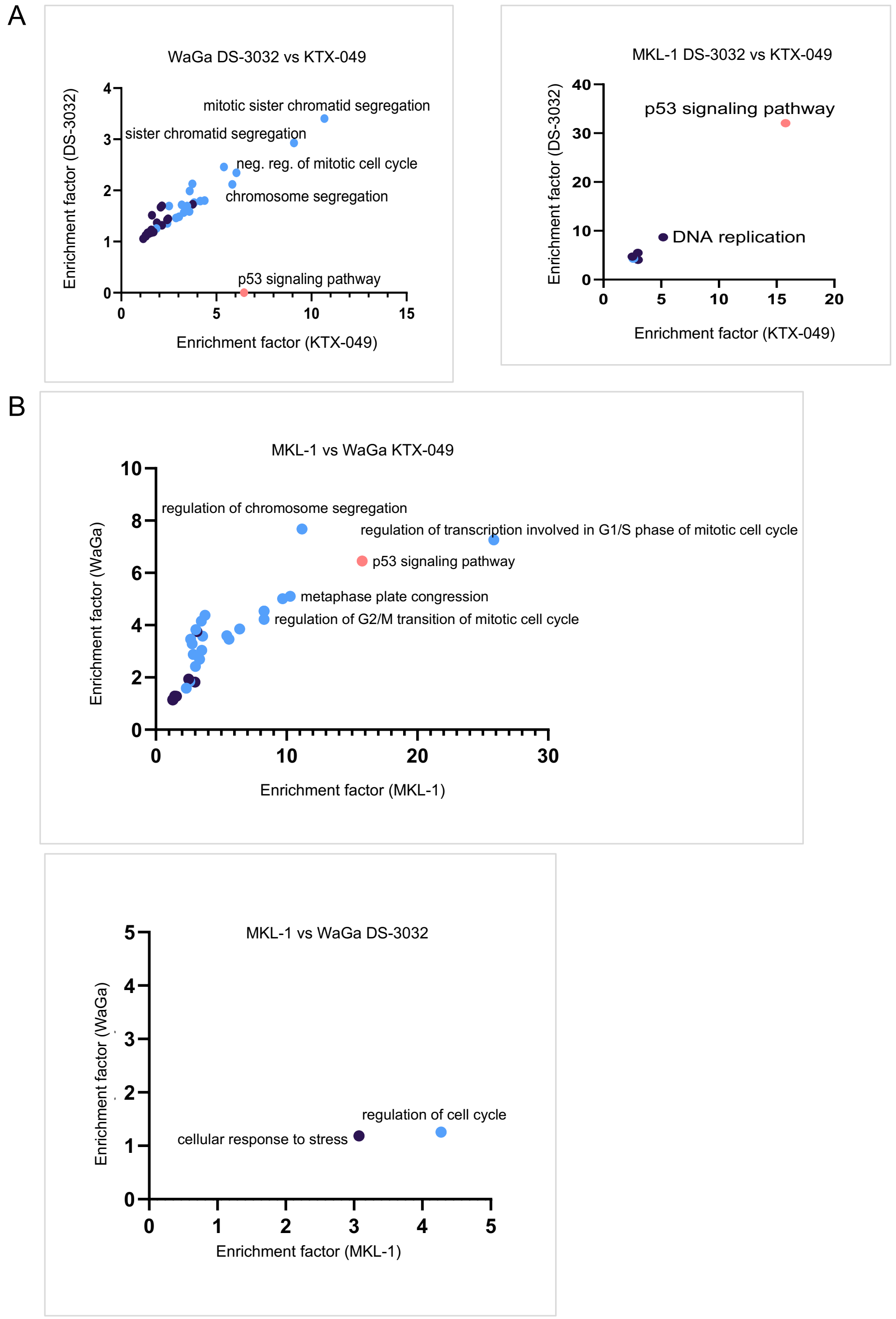

Supplemental Figure 13

Pathway analysis of proteins upregulated or downregulated in WaGa or MKL-1 cells in response to KTX-049 and DS-3032 treatment. (A) Pathways enriched in DS-3032 versus KTX-049 for WaGa (left) and MKL-1 (right). (B) Pathways enriched in WaGa versus MKL-1 cells treated with KTX-049 (top) and DS-3032 (bottom).

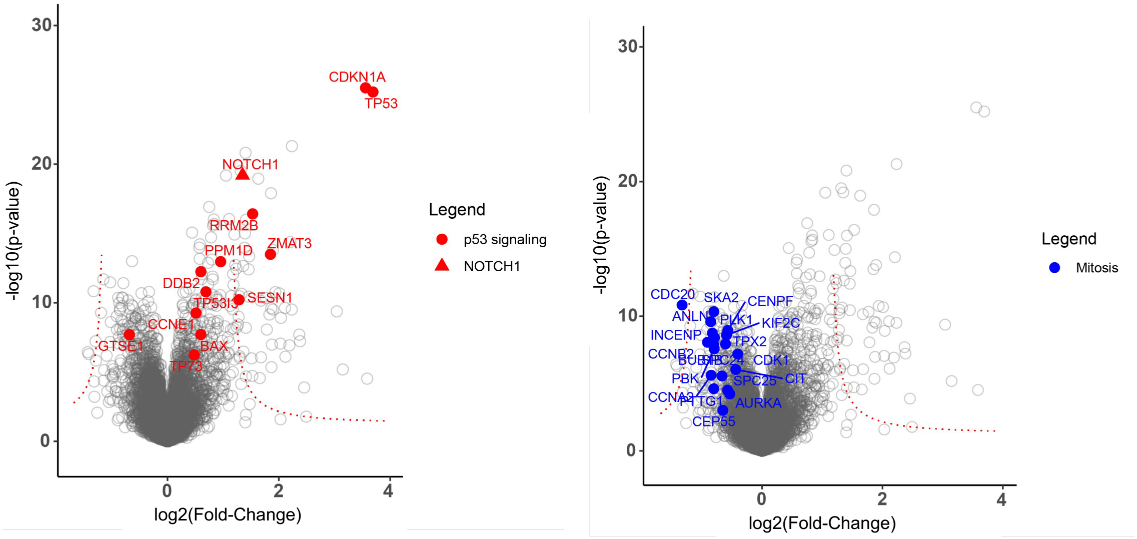

Supplemental Figure 14

Volcano plots show significantly up-regulated or down-regulated proteins in PDX tumors after 24 h of KT-253 treatment compared to proteins detected in tumors after vehicle treatment. Red indicates KEGG p53 response proteins and blue indicates KEGG mitotic proteins.

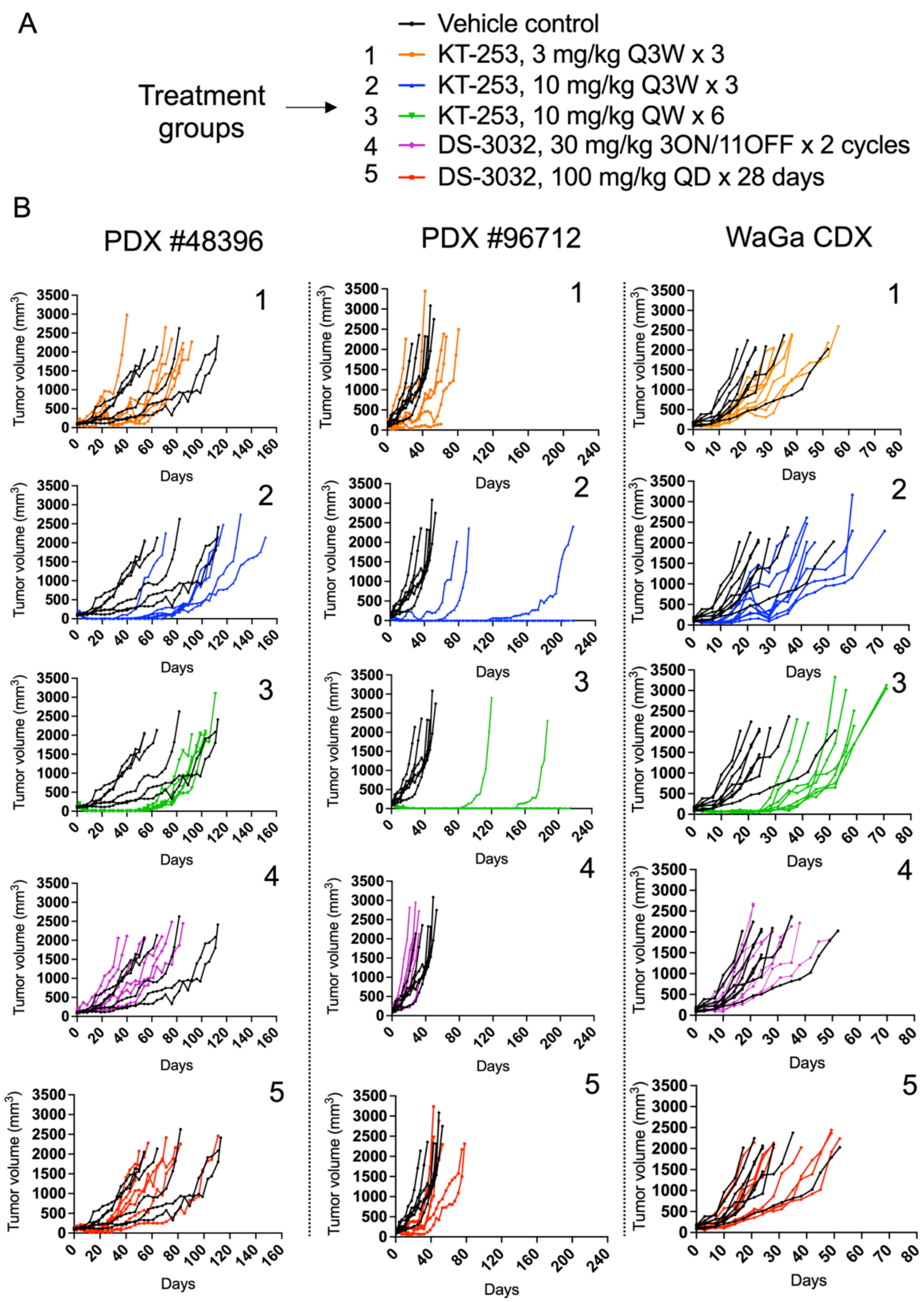

Supplemental Figure 15

Individual tumor trajectories for the PDX and CDX models. (A) Treatment groups denoted by numbers are compared to vehicle control as shown in B. (B) Individual tumor trajectories for the indicated PDX and CDX models.

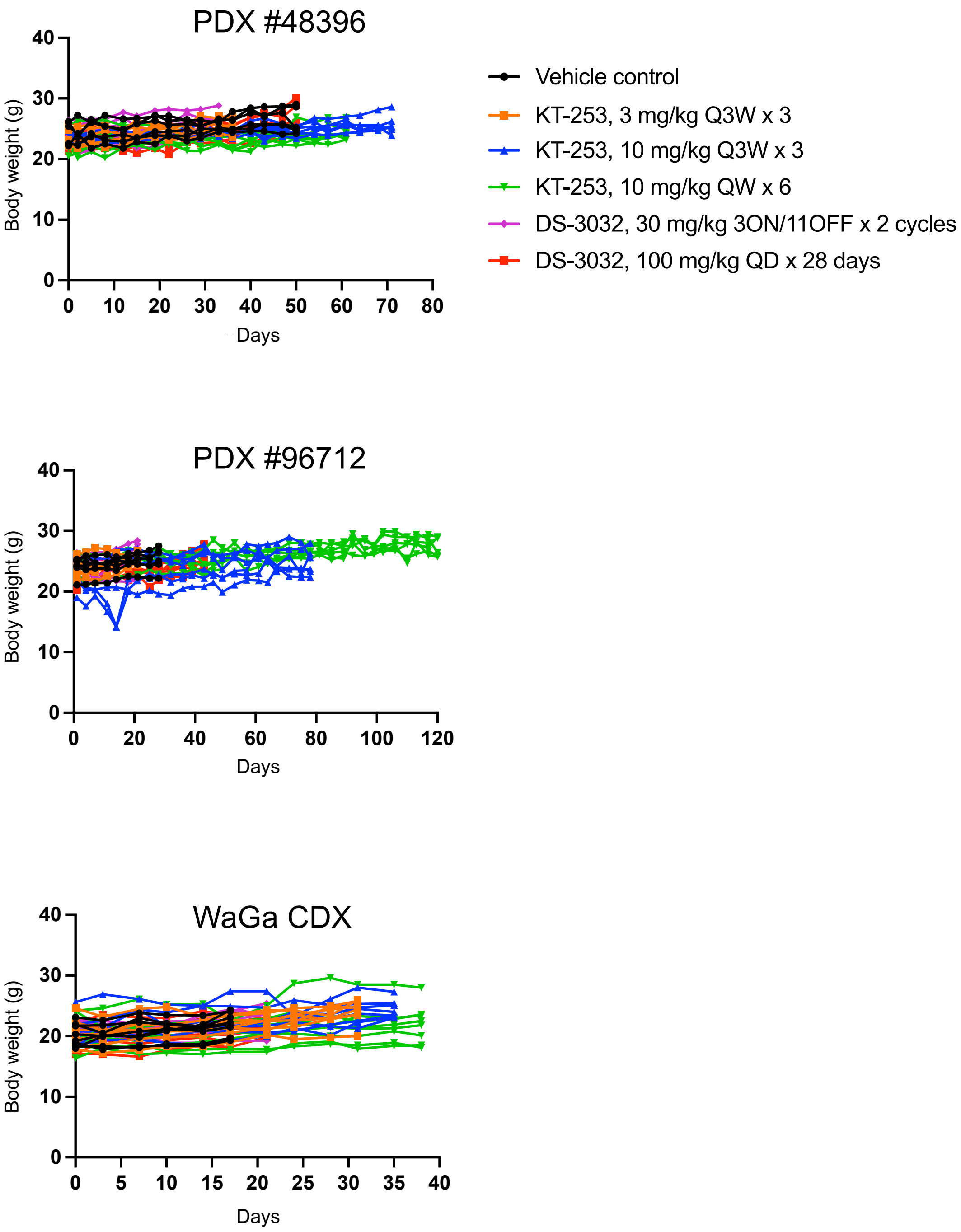

Supplemental Figure 16

Individual body weight trajectories of mice in the PDX and CDX models. Data was plotted till all mice were alive in every treatment arm- 6 in each arm for PDX models and 8 in each arm for CDX model.

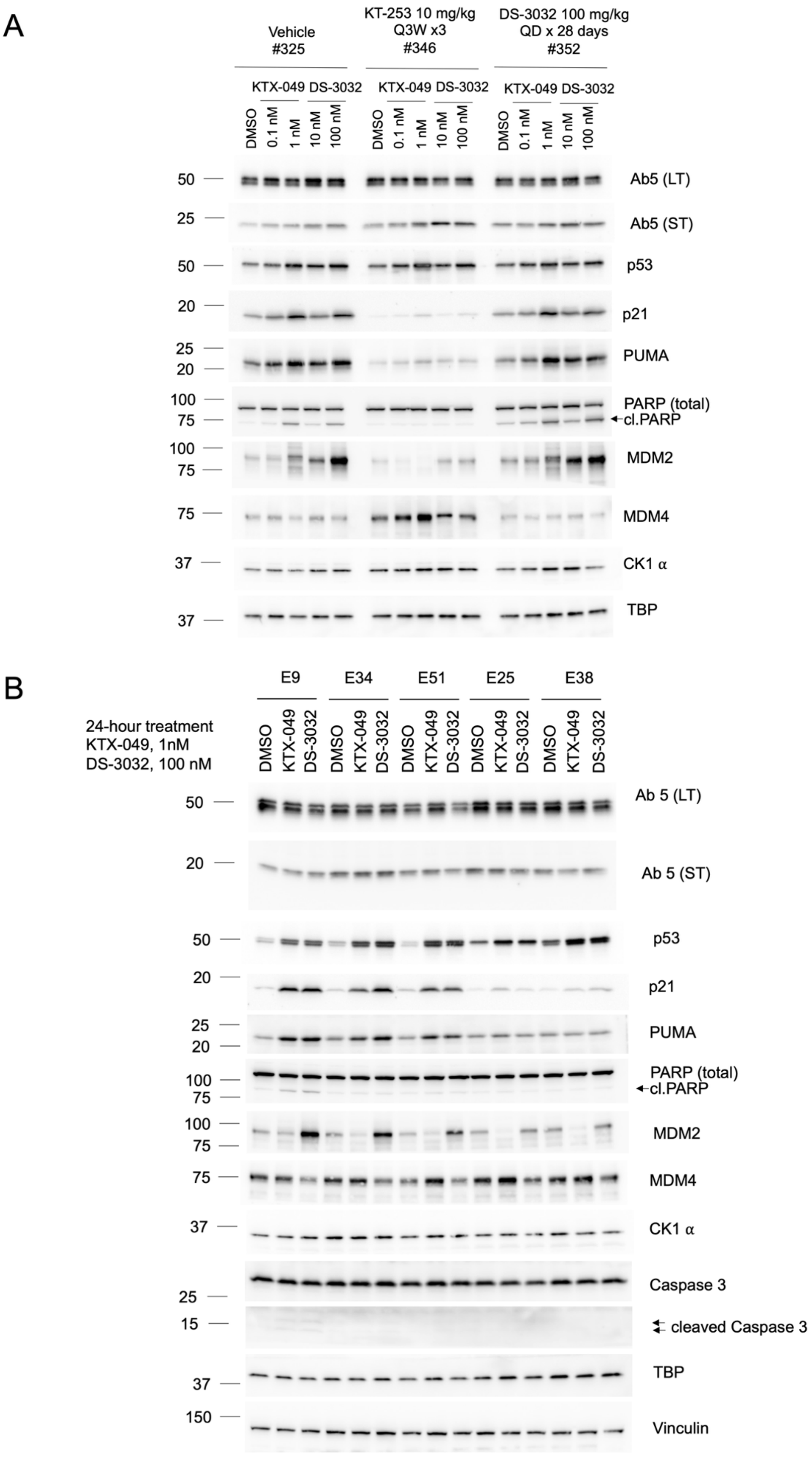
A
Vehicle
#325
KT-253 10 mg/kg
Q3W x3
#346
DS-3032 100 mg/kg
QD x 28 days
#352
KTX-049
DS-3032
DMSO
0.1 nM
1 nM
10 nM
100 nM
Ab5 (LT)
Ab5 (ST)
p53
p21
PUMA
PARP (total)
cl.PARP
MDM2
MDM4
CK1 α
TBP
50
25
20
100
75
37
B
E9
E34
E51
E25
E38
24-hour treatment
KTX-049, 1nM
DS-3032, 100 nM
Ab 5 (LT)
Ab 5 (ST)
Caspase 3
cleaved Caspase 3
Vinculin
15
150

Supplemental Figure 17

(A) PDX #48396 or WaGa CDX derived cell lines were treated with DMSO and the indicated concentrations of KTX-049 or DS-3032 for 24 h and the p53 response was analyzed using WB analysis for the indicated proteins. TBP and/or Vinculin were used as loading controls. Merkel cell polyomavirus ST and LT antigens expressed in MCCP cell lines were detected using the antibody Ab5 and are shown as Ab 5 (LT) and Ab 5 (ST) respectively. Multiple gels were run with equal volumes of the same lysate to analyze the indicated proteins. The membrane for p21 was stripped and re-probed to analyze PUMA levels. N=1 for PDX derived cell lines and N=2 for CDX derived cell lines. Blots are shown from a single representative experiment.

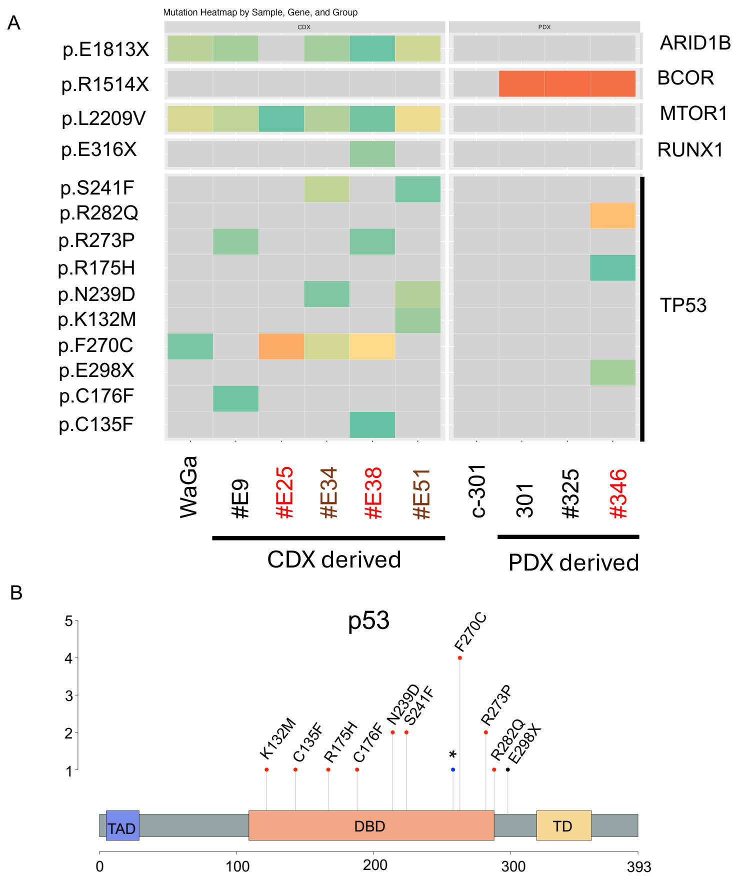

Supplemental Figure 18

Figure showing (A) oncogenic/likely oncogenic variants detected in the CDX derived (left) and PDX derived cell lines (right). C-301 represents matched normal germline for control MCC-301. The cell lines resistant to MDM2 inhibition or degradation in-vitro are shown (red) and the cell lines with partial sensitivity to treatment are shown in brown. (B) Lollipop plot of p53 protein showing location of mutations called from WES of CDX and PDX samples in A classified as oncogenic or likely oncogenic by OncoKB database. The height of each mutation refers to the number of samples it was detected in panel A. The canonical *TP53* transcript NM_000546 was chosen as a reference with domains derived from the PFAM database. TAD is transactivation domain, DBD is DNA binding domain and TD is tetramerization domain. Variant classifications were standardized and color-coded by functional class. The asterisk shows the position of the hemizygous deletion of amino acids 251-253 found in MS-1 cell line in a previous study (4). The MS-1 cell line was not sequenced in this study. Red denotes missense mutation, black denotes nonsense mutation and blue denotes an in-frame deletion.

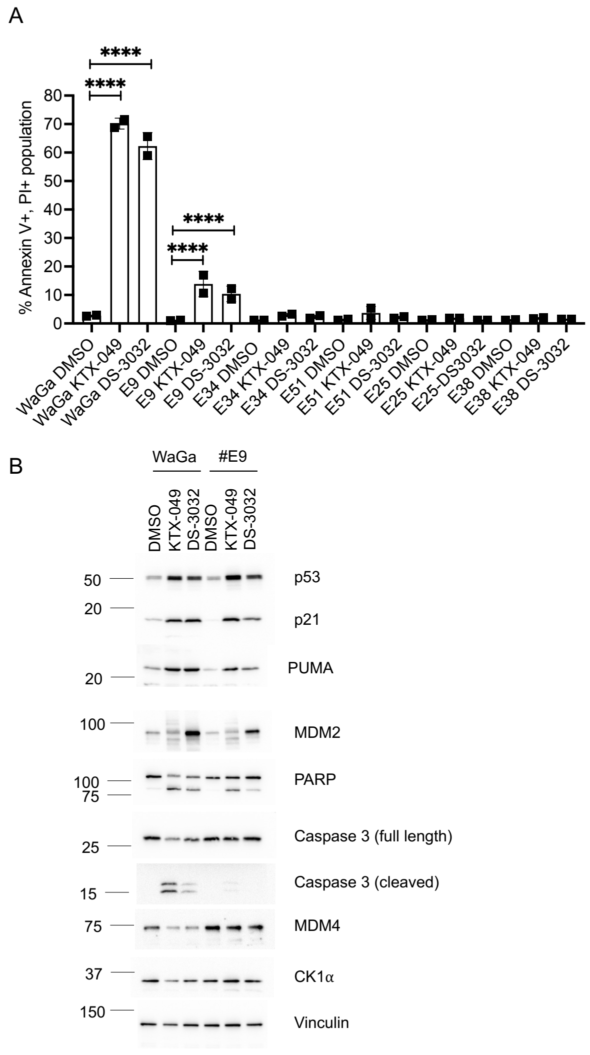

Supplemental Figure 19

(A) WaGa or WaGa CDX derived cell lines were treated with DMSO, 1 nM KTX-049 or 100 nM DS-3032 for 48 h and Annexin V/PI staining was performed. The graph shows the mean values for Annexin V+/PI+ population, N=2. Error bars indicate standard deviation. 2-Way ANOVA with Tukey's multiple comparison test was performed . Significance for treatment with KTX-049 or DS-3032 as compared to the DMSO control for each cell line are shown. **** indicates p-value was <0.0001. (B) WaGa and CDX derived cell line #E9 were treated with DMSO, 1 nM KTX-049 or 100 nM DS-3032 for 24 h and the p53 response was analyzed using WB analysis for the indicated proteins. Vinculin was used as a loading control. N=1. Multiple gels were run with equal volumes of the same lysate to analyze the indicated proteins. Membranes for MDM2 and p21 were stripped and re-probed to analyze the levels of Vinculin and PUMA respectively.

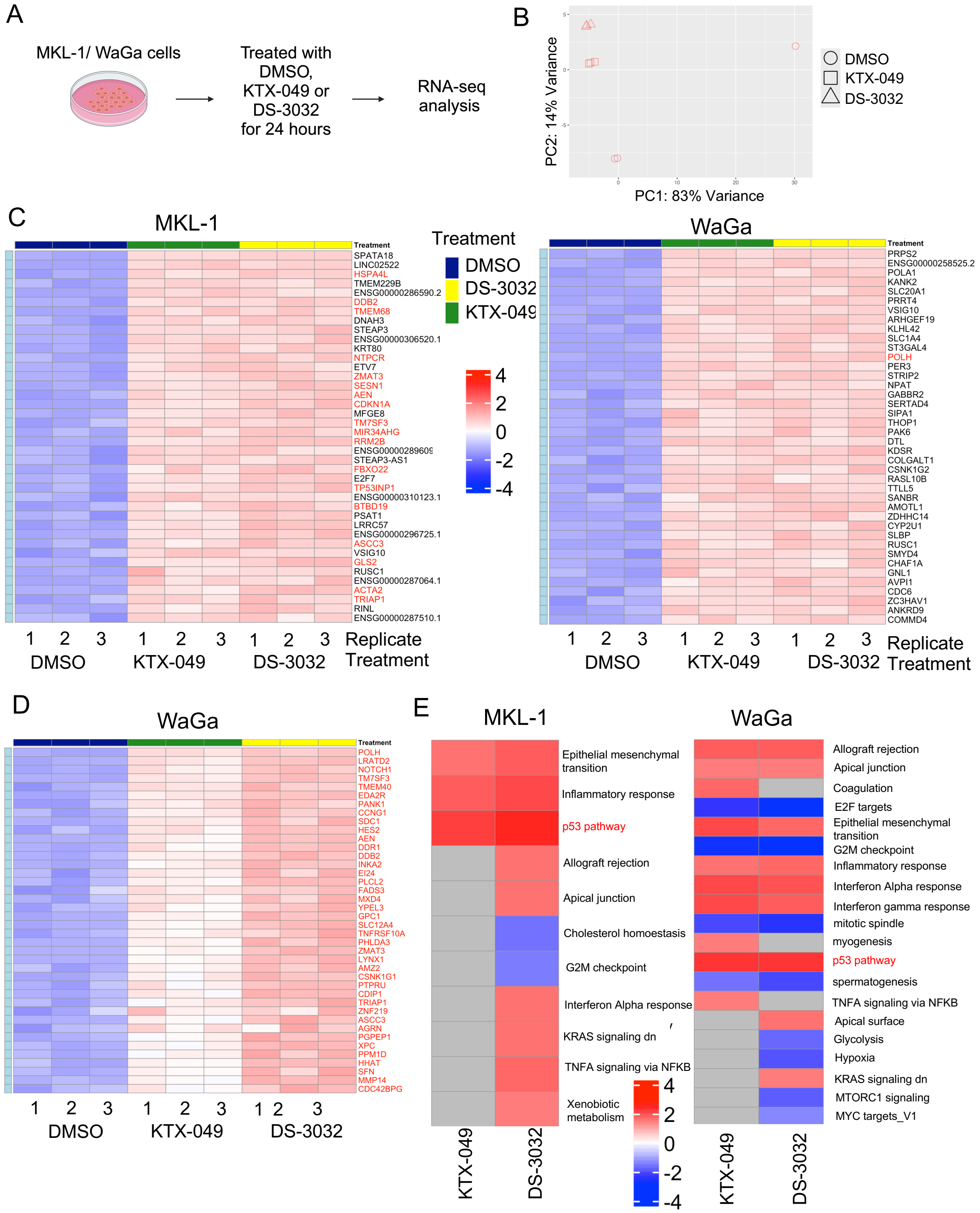
A
MKL-1/ WaGa cells
Treated with DMSO, KTX-049 or DS-3032 for 24 hours
RNA-seq analysis
B
PC2: 14% Variance
PC1: 83% Variance
DMSO
KTX-049
DS-3032
C
MKL-1
Treatment
SPATA18
LINC02522
HSPA4L
TMEM229B
ENSG00000286590.2
DDB2
TMEM68
DNAH3
STEAP3
ENSG00000306520.1
KRT80
NTPCR
ETV7
ZMAT3
SESN1
AEN
CDKN1A
MFGE8
TM7SF3
MIR34AHG
RRM2B
ENSG0000028960S
STEAP3-AS1
FBXO22
E2F7
TP53INP1
ENSG00000310123.1
BTBD19
PSAT1
LRRC57
ENSG00000296725.1
ASCC3
VSIG10
GLS2
RUSC1
ENSG00000287064.1
ACTA2
TRIAP1
RINL
ENSG00000287510.1
1 2 3 1 2 3 1 2 3 Replicate
DMSO KTX-049 DS-3032 Treatment
Treatment
DMSO
DS-3032
KTX-049
4
2
0
-2
-4
WaGa
Treatment
PRPS2
ENSG00000258525.2
POLA1
KANK2
SLC20A1
PRRT4
VSIG10
ARHGEF19
KLHL42
SLC1A4
ST3GAL4
POLH
PER3
STRIP2
NPAT
GABBR2
SERTAD4
SIPA1
THOP1
PAK6
DTL
KDSR
COLGALT1
CSNK1G2
RASL10B
TTLL5
SANBR
AMOTL1
ZDHHC14
CYP2U1
SLBP
RUSC1
SMYD4
CHAF1A
GNL1
AVPI1
CDC6
ZC3HAV1
ANKRD9
COMMD4
1 2 3 1 2 3 1 2 3 Replicate
DMSO KTX-049 DS-3032 Treatment
D
WaGa
Treatment
POLH
LRATD2
NOTCH1
TM7SF3
TMEM40
EDA2R
PANK1
CCNG1
SDC1
HES2
AEN
DDR1
DDB2
INKA2
EI24
PLCL2
FADS3
MXD4
YPEL3
GPC1
SLC12A4
TNFRSF10A
PHLDA3
ZMAT3
LYNX1
AMZ2
CSNK1G1
PTPRU
CDIP1
TRIAP1
ZNF219
ASCC3
AGRN
PGPEP1
XPC
PPM1D
HHAT
SFN
MMP14
CDC42BPG
1 2 3 1 2 3 1 2 3
DMSO KTX-049 DS-3032
E
MKL-1
Epithelial mesenchymal transition
Inflammatory response
p53 pathway
Allograft rejection
Apical junction
Cholesterol homoeostasis
G2M checkpoint
Interferon Alpha response
KRAS signaling dn
TNFA signaling via NFKB
Xenobiotic metabolism
KTX-049
DS-3032
4
2
0
-2
-4
WaGa
Allograft rejection
Apical junction
Coagulation
E2F targets
Epithelial mesenchymal transition
G2M checkpoint
Inflammatory response
Interferon Alpha response
Interferon gamma response
mitotic spindle
myogenesis
p53 pathway
spermatogenesis
TNFA signaling via NFKB
Apical surface
Glycolysis
Hypoxia
KRAS signaling dn
MTORC1 signaling
MYC targets_V1
KTX-049
DS-3032

Supplemental Figure 20

(A) Schematic showing the layout of RNA-seq experiments. MKL-1 and WaGa cells were treated with DMSO, 1 nM KTX-049 or 100 nM DS-3032 for 24 h, RNA was collected and RNA-seq analysis was performed. (B) PCA plot for MKL-1 samples. (C) Heatmaps showing the top 40 differentially regulated genes after pairwise comparisons between MKL-1 (left) and WaGa (right) cells. The light blue square on the left side of the heatmap indicate two significant pairwise comparisons of the gene in each row. Direct p53 target genes with a target gene reg score ≥ 35 are highlighted in red. (D) Heatmap showing the top 40 differentially regulated direct p53 targets with a target gene reg score ≥ 35 in WaGa cells. (E) Heatmap showing GSEA enrichment analysis of differentially regulated genes after treatment in MKL-1 (left) and WaGa (right) sets.

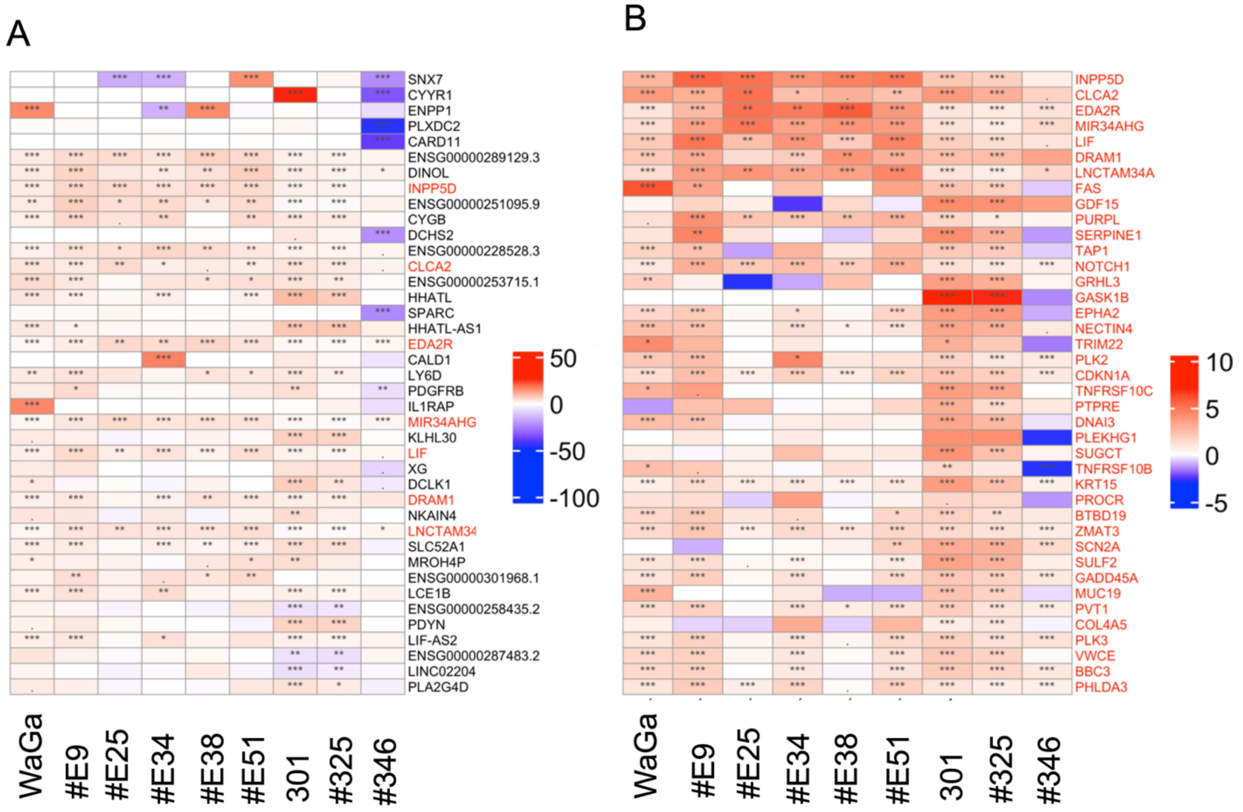

Supplemental Figure 21

(A) The top 40 differentially expressed genes in KTX-049 treated samples as compared to the respective DMSO controls are shown based on log2FC values. Direct p53 targets with a target gene reg score of ≥ 35 are shown in red. (B) Heatmap shows the top 40 differentially expressed direct p53 target genes (by log2FC) in KTX-049 treated samples as compared to their respective DMSO controls.

Supplemental Methods

Antibodies used for Western Blotting

Primary antibodies used include: p53-DO1 (Santacruz Biotechnology, sc-126, RRID:AB_628082), p21 (Cell Signaling Technology, 2946S, RRID:AB_2260325), PUMA (Cell Signaling Technology, 4976S, RRID:AB_2064551), MDM2 (Cell Signaling Technology, 86934S, RRID:AB_2784534), MDM4 (Abcam, 243859), PARP (Cell Signaling Technology, 9542S, RRID:AB_2160739), Caspase-3 (Cell Signaling Technology, 9662S, RRID:AB_331439), Ab5 (DeCaprio Lab, described in (5)), Vinculin (Sigma Aldrich, V9131, RRID:AB_477629), TBP (Cell Signaling Technology, 8515S, RRID:AB_10949159), Notch 1 antibodies D1E11 and D6F11 (Cell Signaling Technology, 3608T, RRID:AB_2153354 and 4380T, RRID:AB_10691684) and cleaved Notch 1 D3B8 (Cell signaling Technology, 4147T, RRID:AB_2153348).

Caspase 3/7 Glo assay

Cells were plated and treated in a 96 well format for viability assays followed by the addition of the Caspase 3/7 reagent (Promega # PAG8093) according to the manufacturer's protocol and luminescence was measured. The relative luminescence was then calculated.

Annexin V/PI staining

One million cells were seeded in a 2 mL volume with DMSO, with the indicated concentrations of KTX-049 or DS-3032 for the indicated times. Cells were collected by centrifugation, washed with PBS, and incubated with Annexin V/PI (BD Biosciences #556547) according to the manufacturer's protocol followed by flow cytometry analysis using a BD Fortessa cytometer. Flow cytometry data were analyzed using the licensed version of FlowJo software (RRID:SCR_008520).

Cell cycle analysis

One million cells were seeded and treated with DMSO or the indicated concentrations of KTX-049 or DS-3032. Cells were incubated with 10 μM EdU (Click Chemistry Tools #1149-100) for one hour before the end of the treatment and collected by centrifugation. Cell pellets were washed with PBS, incubated with 4% formaldehyde (Life Technologies #28906) in PBS for 15 min at room temperature, washed three times with 1% BSA in PBS and fixed using ice cold 70% ethanol. The cells were stored at -20°C until processing. Before staining, the cells were washed twice with PBS and incubated with ClickIT reaction cocktail containing $CuSO_4$, THPTA (Sigma Aldrich #762342), Sodium Ascorbate and CalFluor 647 fluorophore azide (Click Chemistry Tools #CCT-1372) for 30 mins in the dark at room temperature [ClickIT protocol was adapted from (6, 7)]. The cells were collected by centrifugation, washed three times with PBS and incubated with DAPI (1 μg/mL) and RNase A (0.1 μg/mL) solution in PBS for 30 min at room temperature. The stained cell suspensions were passed through tubes with strainer caps, followed by flow cytometry

analysis using a BD Fortessa cytometer. The acquired data were analyzed using the licensed version of the FlowJo software (RRID:SCR_008520).

Trypsin and LysC digestion for TMT based proteome profiling

Dried samples were resuspended in 200 mM EPPS (pH 8.5) and digested at room temperature for 14 h with LysC protease at a 100:1 protein:protease ratio. Trypsin was then added at a 100:1 protein:protease ratio and the reaction was incubated for 6 h at 37°C.

Tandem mass tag labeling

The TMTpro reagent (0.8 mg) was dissolved in anhydrous acetonitrile (40 μL), which 7 μL was added to the peptides (50 μg) with 13 μL of acetonitrile to achieve a final concentration of approximately 30% (v/v). After incubation at room temperature for 1 h, the reaction was quenched with hydroxylamine to a final concentration of 0.3% (v/v). TMTpro-labeled samples were pooled in a 1:1 ratio across all samples. For each experiment, the pooled sample was vacuum centrifuged to near dryness and subjected to C18 solid-phase extraction (SPE) (Sep-Pak, Waters).

Off-line basic pH reversed-phase (BPRP) fractionation

We fractionated the pooled, labeled peptide sample using BPRP HPLC (8) and an Agilent 1260 pump equipped with a degasser and a UV detector (set at 220 and 280 nm wavelengths). Peptides were subjected to a 50-min linear gradient from 5% to 35%

acetonitrile in 10 mM ammonium bicarbonate pH 8 at a flow rate of 0.6 mL/min over an Agilent 300Extend C18 column (3.5 μm particles, 4.6 mm ID and 220 mm in length). The peptide mixture was fractionated into 96 fractions, which were consolidated into 24 super-fractions (9), of which 12 non-adjacent fractions were analyzed. The samples were subsequently acidified with 1% formic acid and vacuum centrifuged to near dryness. Each super-fraction was desalted via StageTip, dried again via vacuum centrifugation, and reconstituted in 5% acetonitrile and 5% formic acid for LC-MS/MS processing.

Liquid chromatography and tandem mass spectrometry

Mass spectrometry data were collected using an Orbitrap Fusion Lumos mass spectrometer coupled with a Proxeon NanoLC-1200 UHPLC. The 100 μm capillary column was packed with 35 cm of Accucore 150 resin (2.6 μm, 150 Å; ThermoFisher Scientific) at a flow rate of 450 nL/min. The scan sequence began with an MS1 spectrum (Orbitrap analysis, resolution 60,000, 350-1350 Th, automatic gain control (AGC) target set to “standard”, maximum injection time set to “auto”). Data were acquired ~90 minutes per fraction. The hrMS2 stage consisted of fragmentation by higher energy collisional dissociation (HCD, normalized collision energy 35%) and analysis using Orbitrap (AGC 200%, maximum injection time 120 ms, isolation window 0.5 Th, resolution 50,000). Data were acquired using the FAIMSpro interface with the dispersion voltage (DV) set to 5,000V, the Compensation voltages (CVs) were set at -30V, -50V, and -70V, and the TopSpeed parameter was set at 1 s per CV.

Data analysis

The spectra were converted to mzXML using MSConvert (10). The database search included all entries from the human UniProt reference database (downloaded June 2024, RRID:SCR_002380). The database was concatenated with one composed of all protein sequences in the database in reverse order. Searches were performed using a 50-ppm precursor ion tolerance for total protein level profiling. Product ion tolerance was set to 0.03 Da. These wide mass tolerance windows were chosen to maximize sensitivity in conjunction with comet searches and linear discriminant analysis (11, 12). TMTpro labels on lysine residues and peptide N-termini +304.207 Da), as well as carbamidomethylation of cysteine residues (+57.021 Da) were set as static modifications, while oxidation of methionine residues (+15.995 Da) was set as a variable modification. Peptide-spectrum matches (PSMs) were adjusted to a 1% false discovery rate (FDR) (13, 14). PSM filtering was performed using a linear discriminant analysis, as described previously (11) and then further assembled to a final protein-level FDR of 1% (14). Proteins were quantified by summing the reporter ion counts across all matching PSMs, as previously described (15). Reporter ion intensities were adjusted to correct for isotopic impurities of the different TMTpro reagents according to the manufacturer's specifications. The signal-to-noise (S/N) measurements of the peptides assigned to each protein were summed and these values were normalized so that the sum of the signal for all proteins in each channel was equivalent to account for equal protein loading. Finally, each protein abundance measurement was scaled, such that the summed signal-to-noise ratio for that protein across all channels was 100, thereby generating a relative abundance (RA) measurement.

Pathway analysis for TMT profiling

Pathway analysis was performed using the proteomics analysis software Perseus (16–18) to determine the terms and annotations associated with significantly regulated proteins after KTX-049 or DS-3032 treatment in WaGa or MKL1 cells. KEGG (RRID:SCR_012773) and GO Biological Process (GOBP) terms were assigned to all the proteins. The data were log transformed, null values were removed and a multiple sample test (ANOVA) was performed to compare KTX-049, DS-3032, and DMSO samples, followed by a post hoc Tukey's Honest Significant Difference (HSD) test to determine the magnitude and direction of all significantly regulated proteins. The results of this test were fed into a hierarchical clustering algorithm to generate a heatmap and clusters were automatically assigned. Finally, the Fischer exact test was used to compare terms associated with the clusters to the whole proteome background. This analysis resulted in the assignment of KEGG and GOBP terms, wherever possible, to the clusters of proteins that were significantly regulated among the three treatment groups. The volcano plots in Supplemental Fig. 9 were annotated with proteins based on KEGG pathways (p53 signaling or mitotic signaling) and only proteins with a significance cut-off of $-Log_{10}$(p-value) > 3 were labeled in the plot.

MCC301 (PDX #48396) Single Dose PK

From the 55 mice implanted with MCC301 (PDX #48396), n=6 animals were enrolled in a PK study where they received a single dose of either vehicle or 10 mg/kg KT-253 once tumor volumes reached 262.8-621.9 mm3. After dosing, tumors were harvested at the 24 h endpoint and snap frozen in liquid nitrogen. Frozen flash samples were stored at -80°C until DIA-based proteomics analysis was performed.

Generation of cell lines from tumors

The isolation protocol was adapted from Lee et al., 2022 (7). One third of PDX or CDX tumors harvested at the end-point were stored on ice for a maximum of 2 h before processing. Tumors were washed twice with ice cold PBS and minced into fine pieces using a scalpel. Tumor pieces were homogenized in NSA-C medium supplemented with Amphotericin B (1:100, Gibco, #15290026), Collagenase IV and Hyaluronidase (Sigma Aldrich #C0130 and #H3506 at 2 mg/ml final concentration) by placing on a rotator in a mammalian cell incubator for 3 to 4 h. The homogenous solution was passed through a 70 or 100 mM filter to remove any debris. The filtered cell suspension was centrifuged, and the cell pellet was resuspended in NSA-C medium supplemented with Amphotericin B. Amphotericin B was used for approximately one week after cell isolation. For all in vitro assays, cells were cultured in NSA-C medium.

RNA-seq

Treatment, collection and sequencing

MKL-1, WaGa, PDX- or CDX-derived cells were treated with DMSO, KTX-049, or DS-3032 for 24 h. Cells were collected by centrifugation, resuspended in TRIzol LS reagent and incubated at room temperature for five min. The homogenized samples were incubated with chloroform followed by centrifugation. The aqueous layer was transferred to an RNAeasy spin column and the Qiagen kit protocol for RNA extraction was followed (Qiagen # 74134). RNA QC analysis, mRNA library preparation (poly A enrichment), and sequencing (PE150, 15G raw data per sample) using NovaSeq X plus Series were carried out by Novogene Corporation.

Sequencing Analysis

Paired-end RNA sequencing data were aligned to hg38 by applying STAR (RRID:SCR_004463, v2.7.10) in 1-pass mapping with GENCODE (RRID:SCR_014966, v47) as the gene transcript set (19). The number of reads mapped to each gene was calculated by differential expression analysis. Genes with counts $\geq$ 10 in at least three samples were retained for further analysis. Differential expression analysis was performed using DESeq2 (SCR_015687, v1.42.1) (20) and Q-values were derived using Qvalue (v2.34.0) to control the false discovery rate (21). For exploratory data analysis, replicates were treated as individual samples and results were filtered to a 0.5 % threshold and sorted by difference in average expression between treatment groups. Further analyses were based on an adjusted DESeq2 run which accounted for replicate variability by estimating dispersion across replicates and modeling mean expression per condition.

KTX-049 and DMSO treated samples were compared and differentially expressed genes were determined for each cell line. The Q-values were calculated to assess statistical significance. Two methods were employed for gene selection: counting how often a gene was differentially expressed based on a Q-value threshold of 0.05 or summing the absolute log2 fold changes across comparisons to select genes with the highest total changes. Selected genes were ordered by descending total absolute log2 fold change across all comparisons. Heatmaps were generated, limiting the results to the top 40 genes based on these criteria, with P53 target genes highlighted in red if their expression score was 35 or higher, as reported in the targetgenereg.org database (3). Gene set enrichment analysis was performed using fgsea (v1.28.0) (22) R package. Benjamini-

Hochberg adjusted p-values, calculated by fgsea, were used to filter computed normalized enrichment scores to a 5% threshold.

Whole Exome sequencing

Treatment, collection and sequencing

PDX- or CDX-derived cell lines were collected by centrifugation and genomic DNA was isolated using a kit (Qiagen # 69504). Genomic DNA from the blood of subject 301 was available sand was sent for sequencing. DNA sample QC, DNA library preparation (Agilent V6), and sequencing (PE150, 12G raw data per sample, NovaSeq) were performed by Novogene Corporation.

Preprocessing / Variant Discovery

Bam files were processed with samtools (RRID:SCR_002105, v1.11) (23) and Picard (RRID:SCR_006525, v2.6) (24), and the R package XenofilteR (RRID:SCR_026196 , v1.6) was used to remove mouse reads from PDX models (25). Data quality was assessed using CollectHsMetrics from Picard (24).

GATK Mutect2 (RRID:SCR_026692 , v4.2.6.1) (26–28) was run in tumor-only mode for somatic short variant discovery. Following GATK recommendations, the hg38 1000 genomes panel of normal curated by the Broad Institute and the gnomAD germline resource were used with Mutect2 to reduce false positives and avoid calling common germline variants. The raw variant calls were subsequently filtered with FilterMutectCalls from GATK (RRID:SCR_001876) using default options. Variants with a total depth of coverage of less than 20 or an alternate allelic depth of less than 3 were removed. The remaining mutations were annotated for oncogenicity using OncoKB API (v4.19) (29, 30).

Visualization was performed using ggplot2 (RRID:SCR_014601, v3.5.1) (31) and complexHeatmap (RRID:SCR_017270, v2.18.0) (32) in R software (v4.3.3) (33).

Supplemental References

1. Vecchio DD, Murray RM. *Biomolecular Feedback Systems*. Princeton University Press; 2014.

2. Eliaš J, Macnamara CK. Mathematical Modelling of p53 Signalling during DNA Damage Response: A Survey. *Int J Mol Sci*. 2021;22(19):10590.

3. Fischer M, et al. TargetGeneReg 2.0: a comprehensive web-atlas for p53, p63, and cell cycle-dependent gene regulation. *NAR Cancer*. 2022;4(1):zcac009.

4. Houben R, et al. Mechanisms of p53 Restriction in Merkel Cell Carcinoma Cells Are Independent of the Merkel Cell Polyoma Virus T Antigens. *Journal of Investigative Dermatology*. 2013;133(10):2453–2460.

5. Cheng J, et al. Merkel Cell Polyomavirus Large T Antigen Has Growth-Promoting and Inhibitory Activities. *J Virol*. 2013;87(11):6118–6126.

6. Branigan TB, et al. MMB-FOXM1-driven premature mitosis is required for CHK1 inhibitor sensitivity. *Cell Rep*. 2021;34(9):108808.

7. Lee PC, et al. Reversal of viral and epigenetic HLA class I repression in Merkel cell carcinoma. *J Clin Invest*. 2022;132(13):e151666.

8. Wang Y, et al. Reversed-phase chromatography with multiple fraction concatenation strategy for proteome profiling of human MCF10A cells. *Proteomics*. 2011;11(10):2019–2026.

9. Paulo JA, et al. Quantitative mass spectrometry-based multiplexing compares the abundance of 5000 S. cerevisiae proteins across 10 carbon sources. *J Proteomics*. 2016;148:85–93.

10. Chambers MC, et al. A Cross-platform Toolkit for Mass Spectrometry and Proteomics. *Nat Biotechnol*. 2012;30(10):918–920.

11. Huttlin EL, et al. A Tissue-Specific Atlas of Mouse Protein Phosphorylation and Expression. *Cell*. 2010;143(7):1174–1189.

12. Beausoleil SA, et al. A probability-based approach for high-throughput protein phosphorylation analysis and site localization. *Nat Biotechnol*. 2006;24(10):1285–1292.

13. Elias JE, Gygi SP. Target-Decoy Search Strategy for Mass Spectrometry-Based Proteomics. *Methods Mol Biol*. 2010;604:55–71.

14. Elias JE, Gygi SP. Target-decoy search strategy for increased confidence in large-scale protein identifications by mass spectrometry. *Nat Methods*. 2007;4(3):207–214.

15. McAlister GC, et al. Increasing the multiplexing capacity of TMT using reporter ion isotopologues with isobaric masses. *Anal Chem*. 2012;84(17):7469–7478.

16. Cox J, et al. Accurate Proteome-wide Label-free Quantification by Delayed Normalization and Maximal Peptide Ratio Extraction, Termed MaxLFQ. *Mol Cell Proteomics*. 2014;13(9):2513–2526.

17. Tyanova S, Temu T, Cox J. The MaxQuant computational platform for mass spectrometry-based shotgun proteomics. *Nature Protocols*. 2016;11(12):2301–2320.

18. Tyanova S, Cox J. Perseus: A Bioinformatics Platform for Integrative Analysis of Proteomics Data in Cancer Research. In: von Stechow L, ed. *Cancer Systems Biology: Methods and Protocols*. New York, NY: Springer; 2018:133–148.

19. Mudge JM, et al. GENCODE 2025: reference gene annotation for human and mouse. *Nucleic Acids Research*. 2025;53(D1):D966–D975.

20. Love MI, Huber W, Anders S. Moderated estimation of fold change and dispersion for RNA-seq data with DESeq2. *Genome Biology*. 2014;15(12):550.

21. qvalue [Internet]. *Bioconductor*. http://bioconductor.org/packages/qvalue/. Accessed April 2, 2025.

22. Korotkevich G, Sukhov V, Sergushichev A. Fast gene set enrichment analysis [preprint]. 2019;060012.

23. Danecek P, et al. Twelve years of SAMtools and BCFtools. *GigaScience*. 2021;10(2):giab008.

24. Picard Tools - By Broad Institute [Internet]. https://broadinstitute.github.io/picard/. Accessed April 2, 2025.

25. Kluin RJC, et al. XenofilteR: computational deconvolution of mouse and human reads in tumor xenograft sequence data. *BMC Bioinformatics*. 2018;19(1):366.

26. McKenna A, et al. The Genome Analysis Toolkit: A MapReduce framework for analyzing next-generation DNA sequencing data. *Genome Res*. 2010;20(9):1297–1303.

27. DePristo MA, et al. A framework for variation discovery and genotyping using next-generation DNA sequencing data. *Nat Genet*. 2011;43(5):491–498.

28. Van der Auwera GA, et al. From FastQ data to high confidence variant calls: the Genome Analysis Toolkit best practices pipeline. *Curr Protoc Bioinformatics*. 2013;11(1110):11.10.1-11.10.33.

29. Suehnholz SP, et al. Quantifying the Expanding Landscape of Clinical Actionability for Patients with Cancer. *Cancer Discov*. 2024;14(1):49–65.

30. Chakravarty D, et al. OncoKB: A Precision Oncology Knowledge Base. *JCO Precis Oncol*. 2017;1:PO.17.00011.

31. Create Elegant Data Visualisations Using the Grammar of Graphics [Internet]. https://ggplot2.tidyverse.org./. Accessed April 2, 2025.

32. Gu Z. Complex heatmap visualization. *Imeta*. 2022;1(3):e43.

33. R: The R Project for Statistical Computing [Internet]. https://www.r-project.org/. Accessed April 2, 2025.
