# Supplementary material for: Targeted degradation of MDM2 overcomes feedback regulation of p53 signaling in Merkel cell carcinoma models
Source: J Clin Invest. 2026 Jul 1;136(13):e199049. doi: 10.1172/JCI199049 (PMC13318125; doi:10.1172/JCI199049)

Figure 2A
(MKL-1 panel)

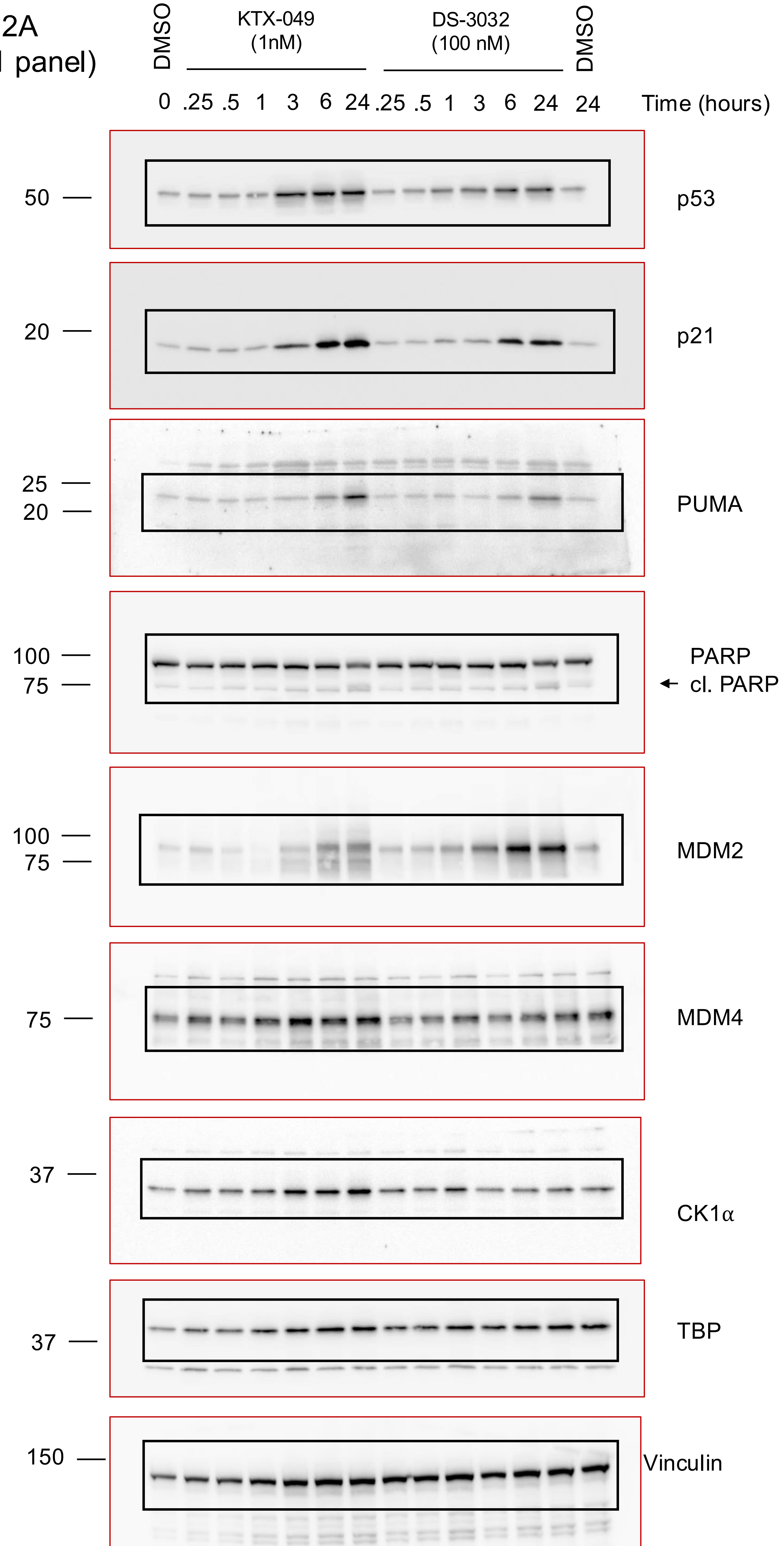

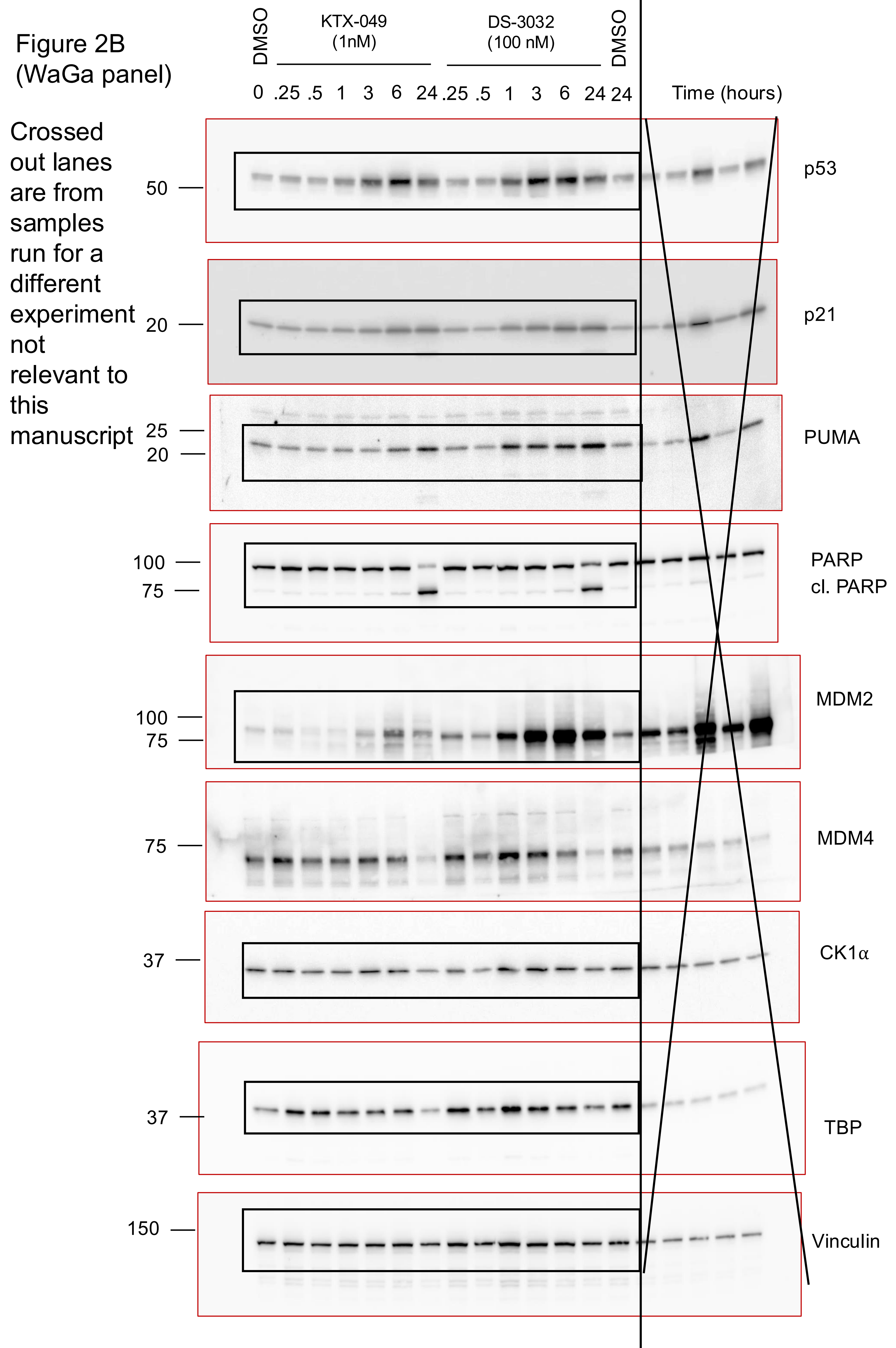

Figure 2B
(WaGa panel)
Crossed out lanes are from samples run for a different experiment not relevant to this manuscript
DMSO
KTX-049
(1nM)
DS-3032
(100 nM)
DMSO
0 .25 .5 1 3 6 24 .25 .5 1 3 6 24 24
Time (hours)
50
p53
20
p21
25
20
PUMA
100
75
PARP
cl. PARP
100
75
MDM2
75
MDM4
37
CK1α
37
TBP
150
Vinculin

Figure 2C
(MKL-1 panel)

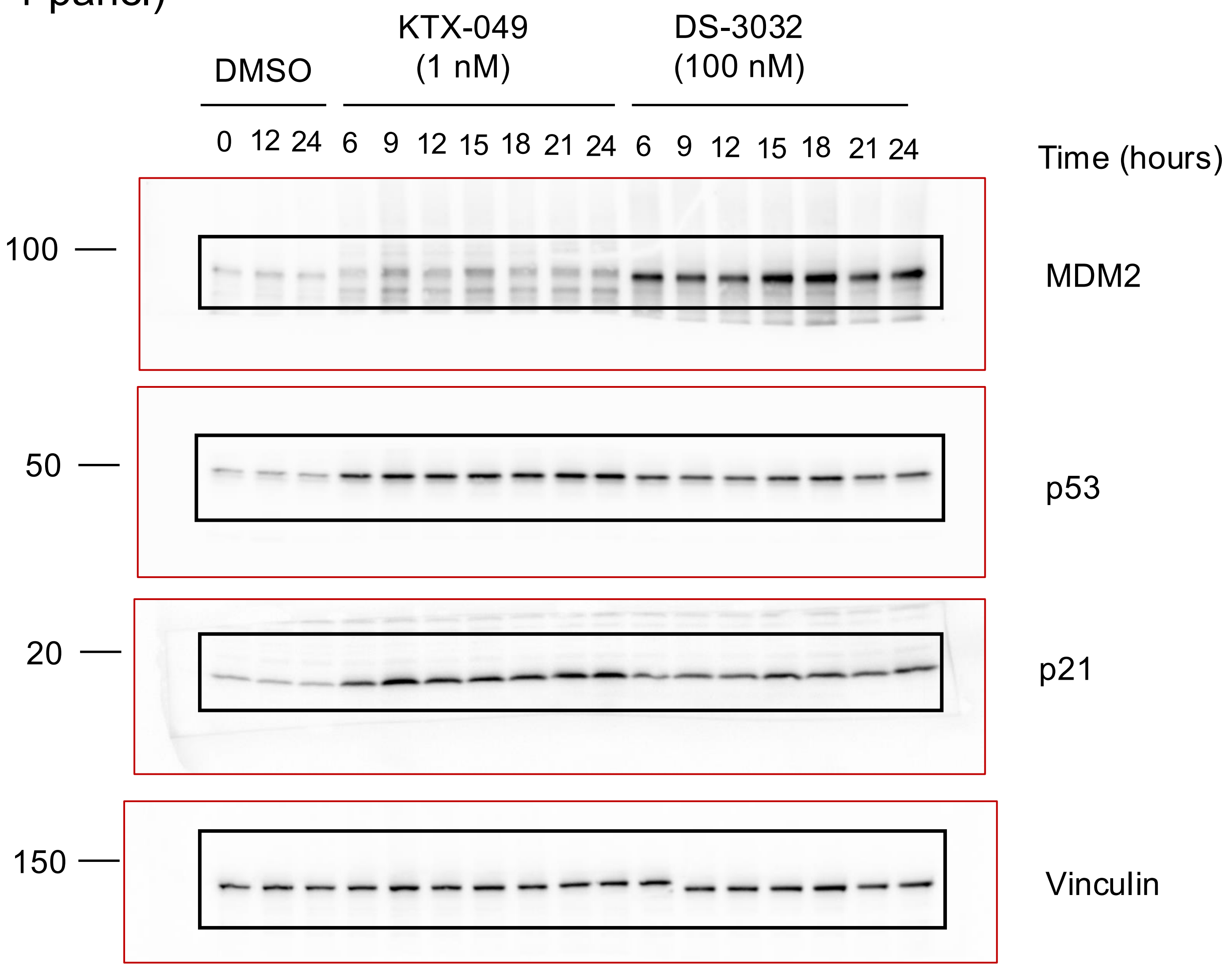

Figure 2D
(WaGa panel)

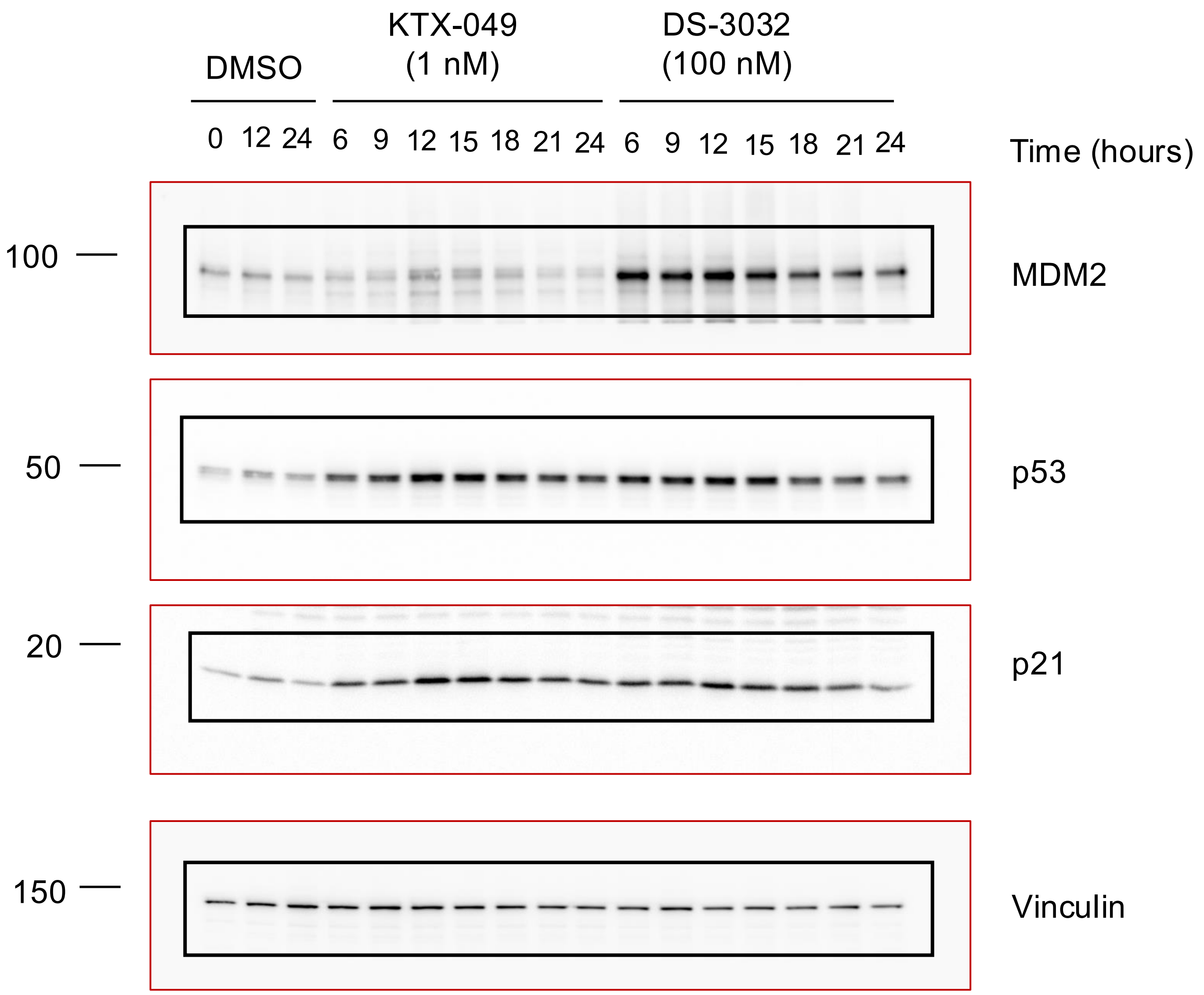

Supplemental Figure 2B (UISO panel)

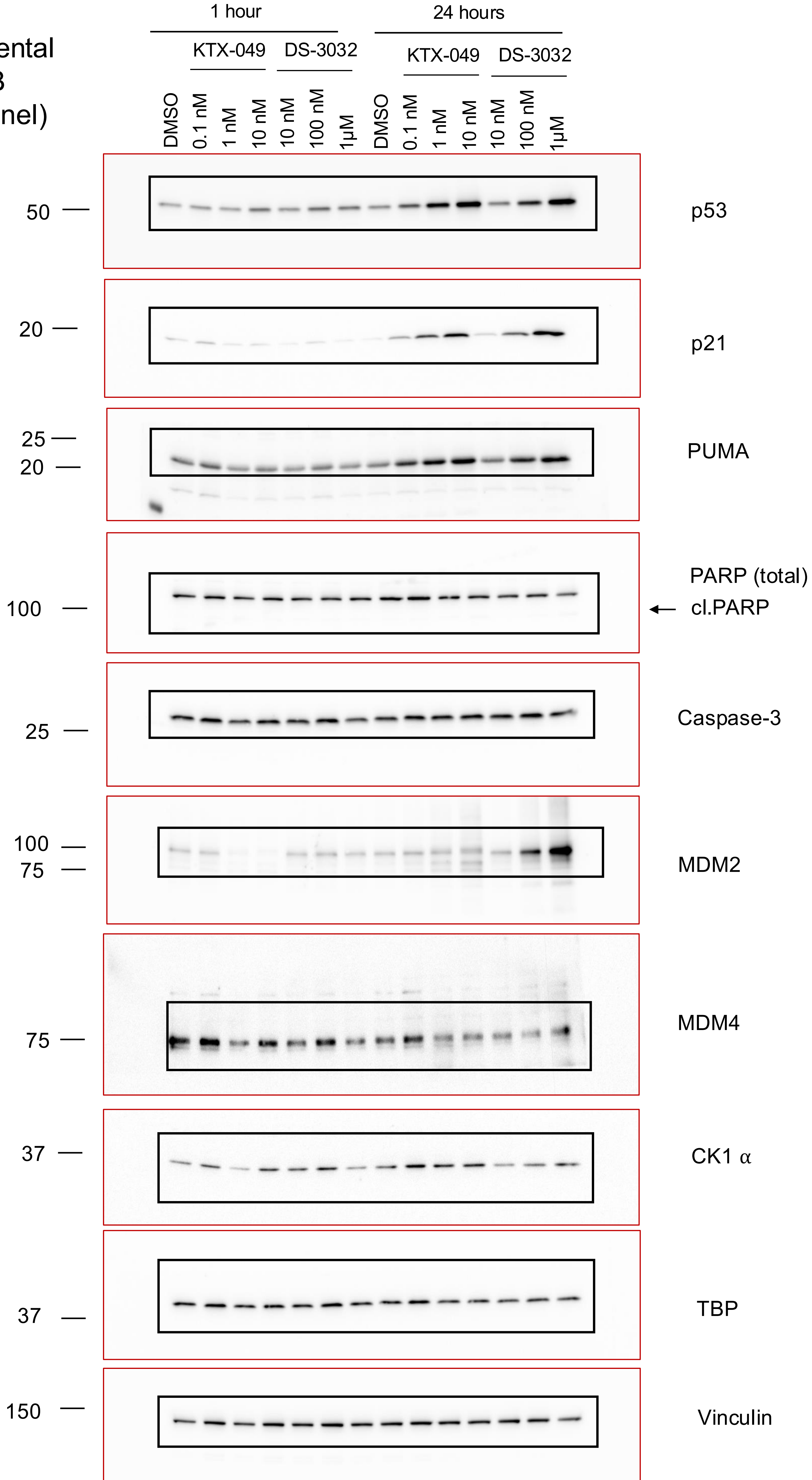

Supplemental
Figure 3
(MKL-1 panel)

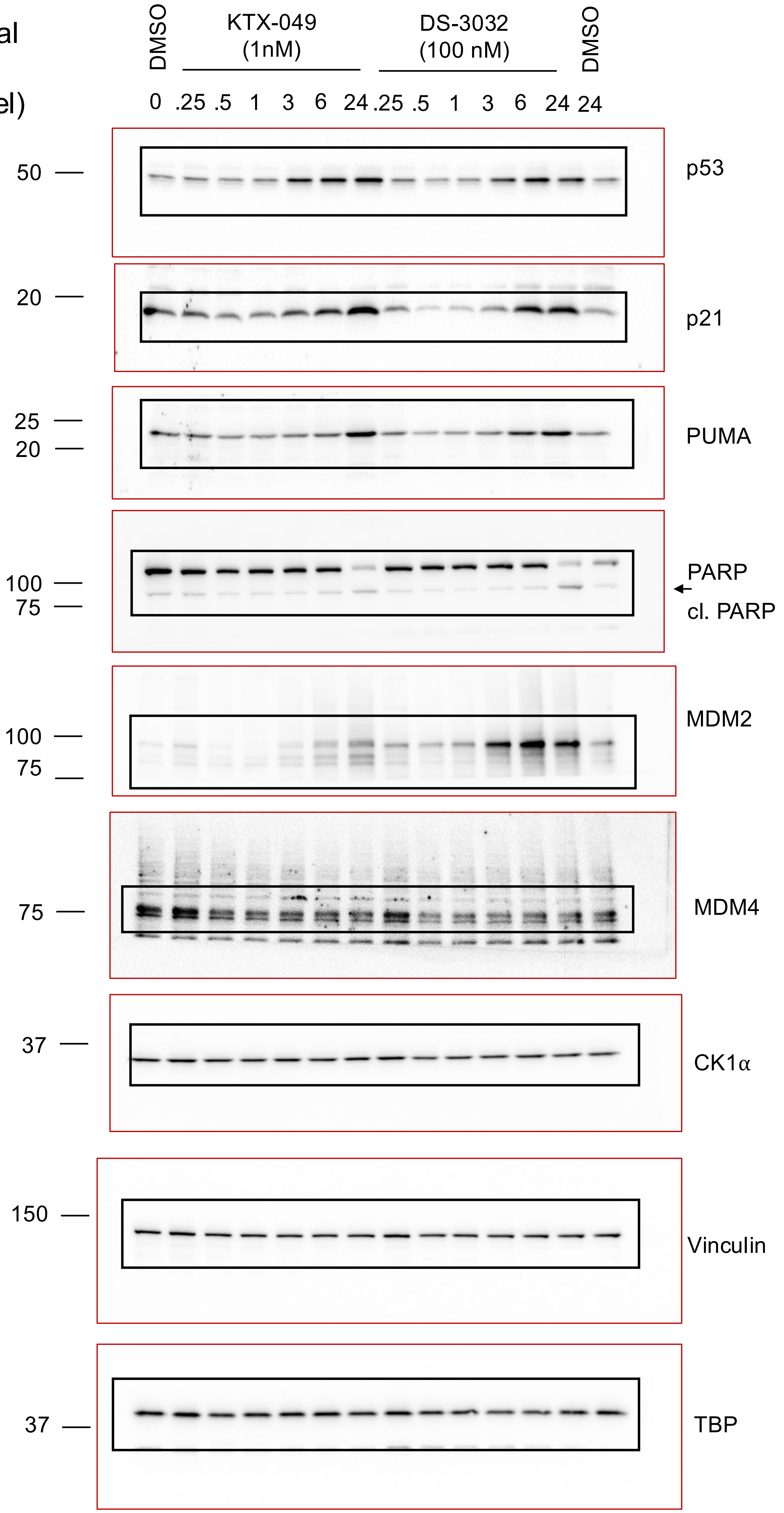

Supplemental Figure 3 (WaGa panel)

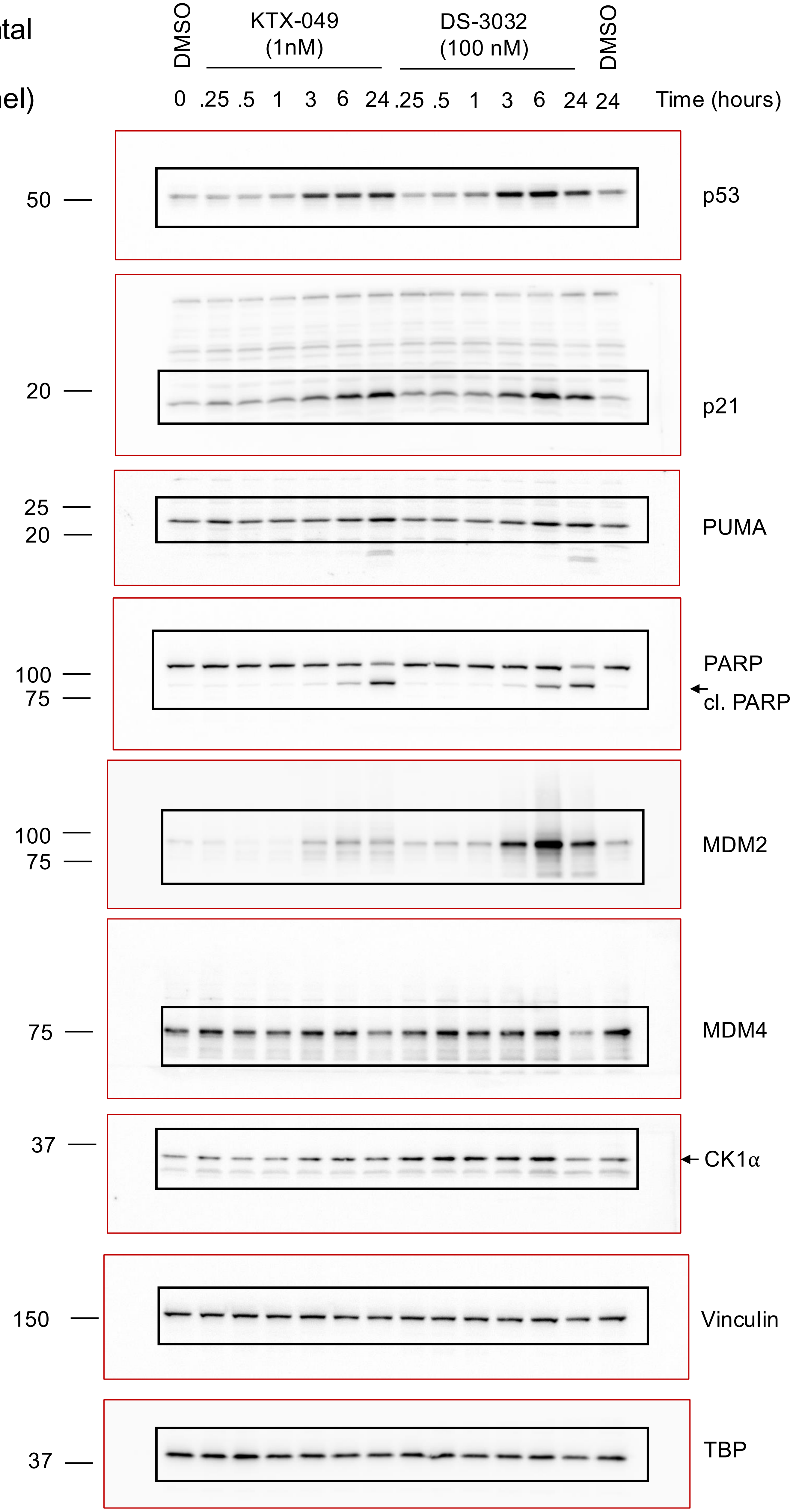

Supplemental Figure 4

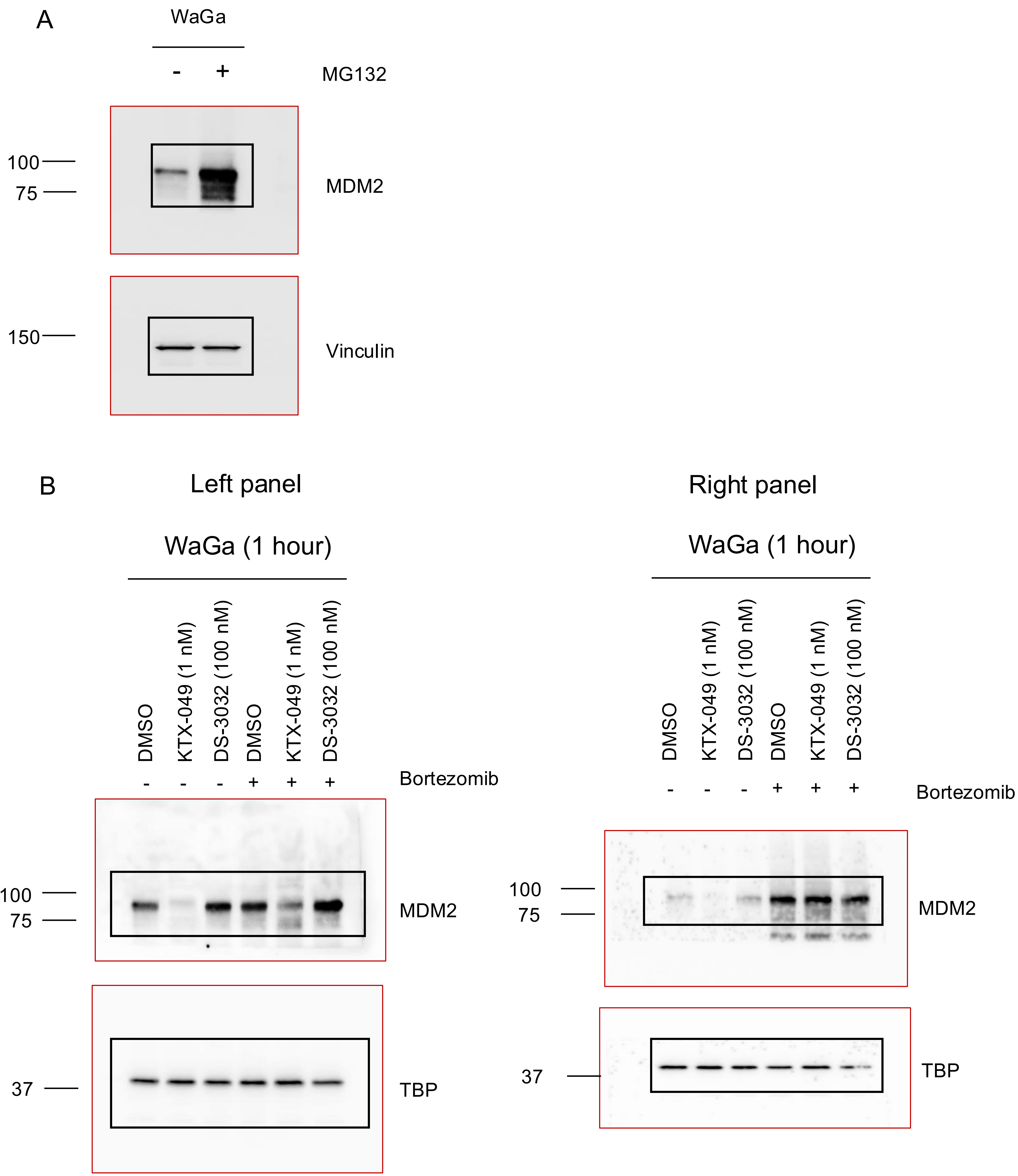

Supplemental Figure 5A

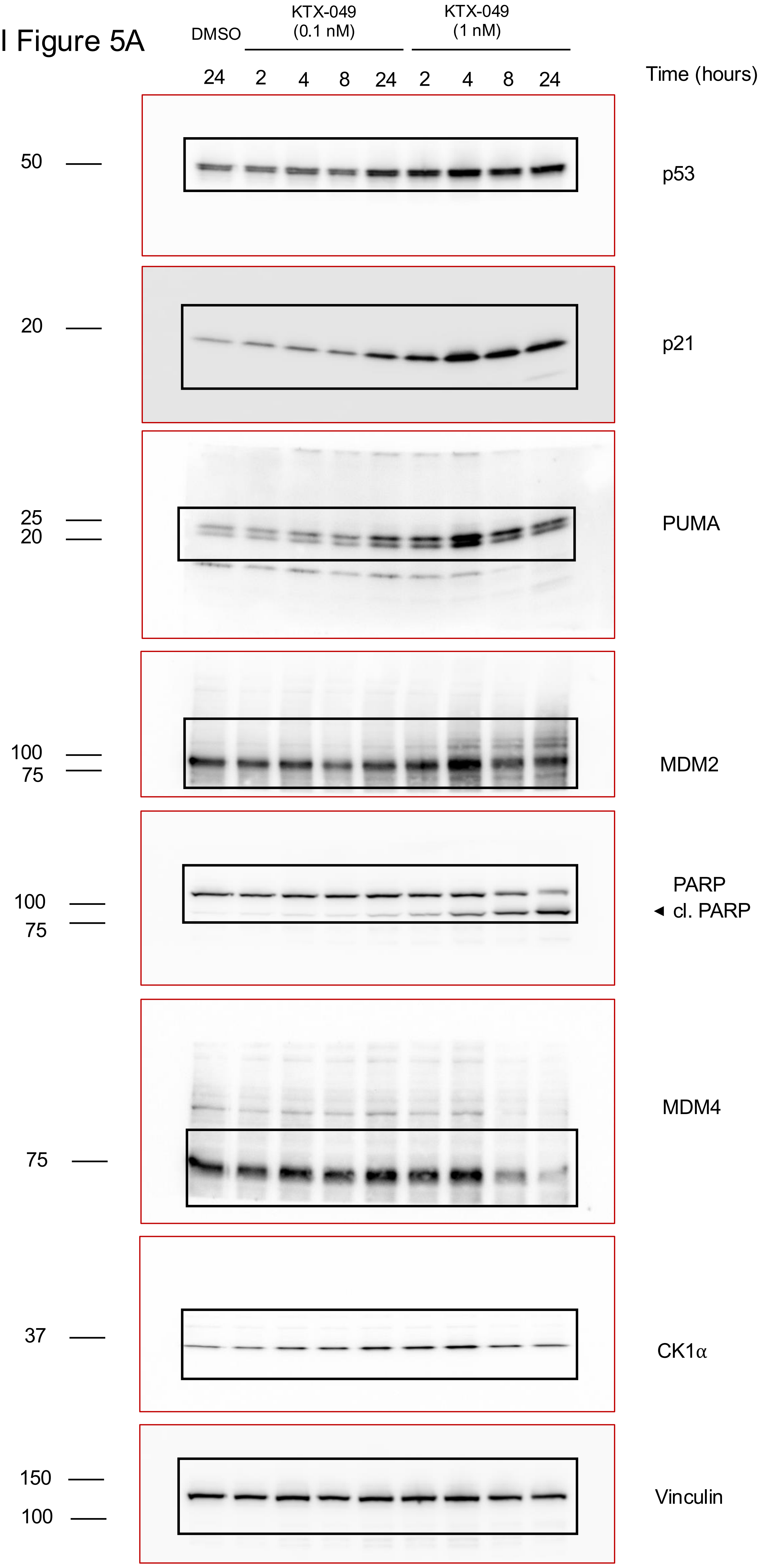

Supplemental Figure 5B

C is DMSO control
K is KTX-049
D is DS-3032

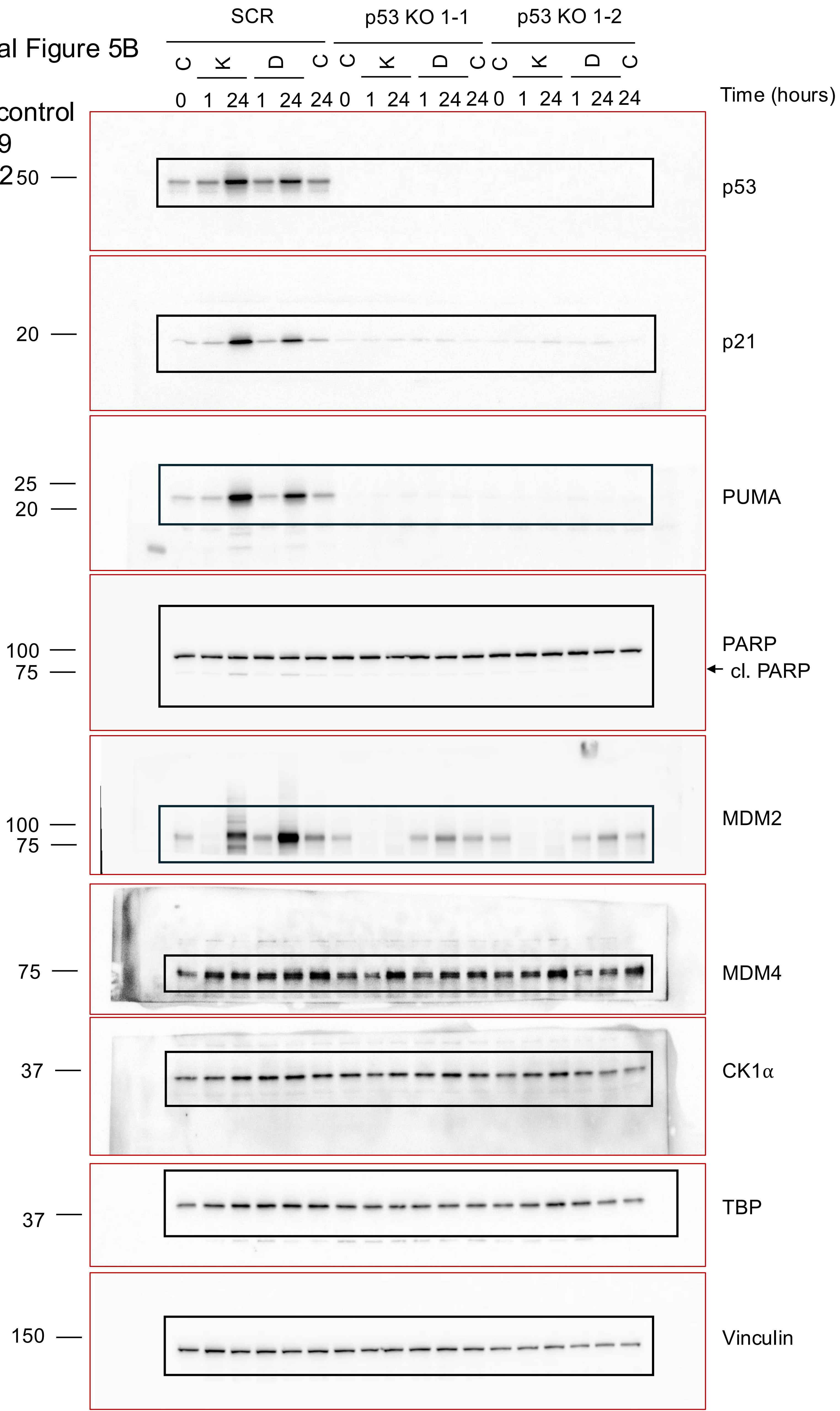

Supplemental Figure 12

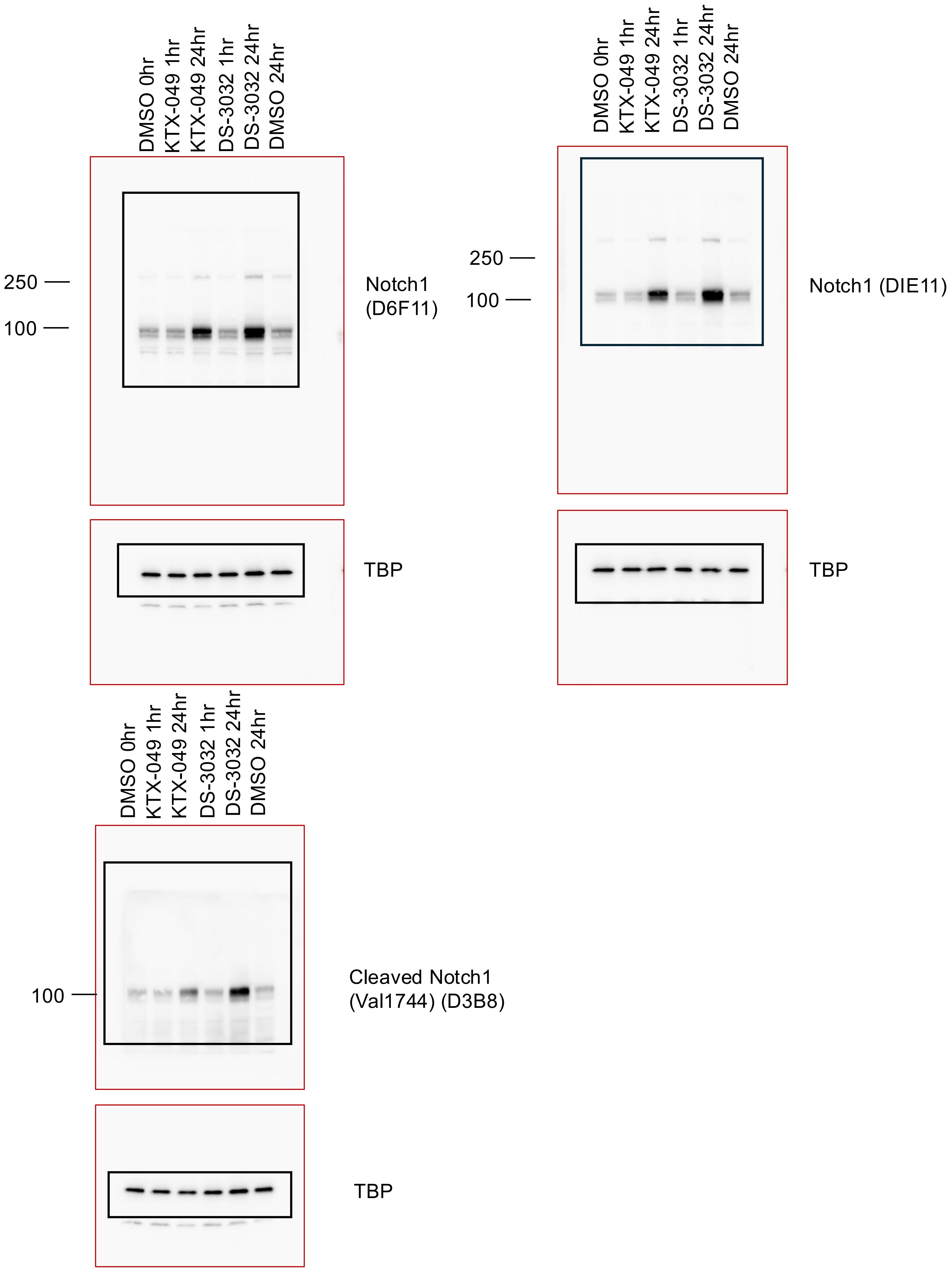

Supplemental Figure 17A Part I

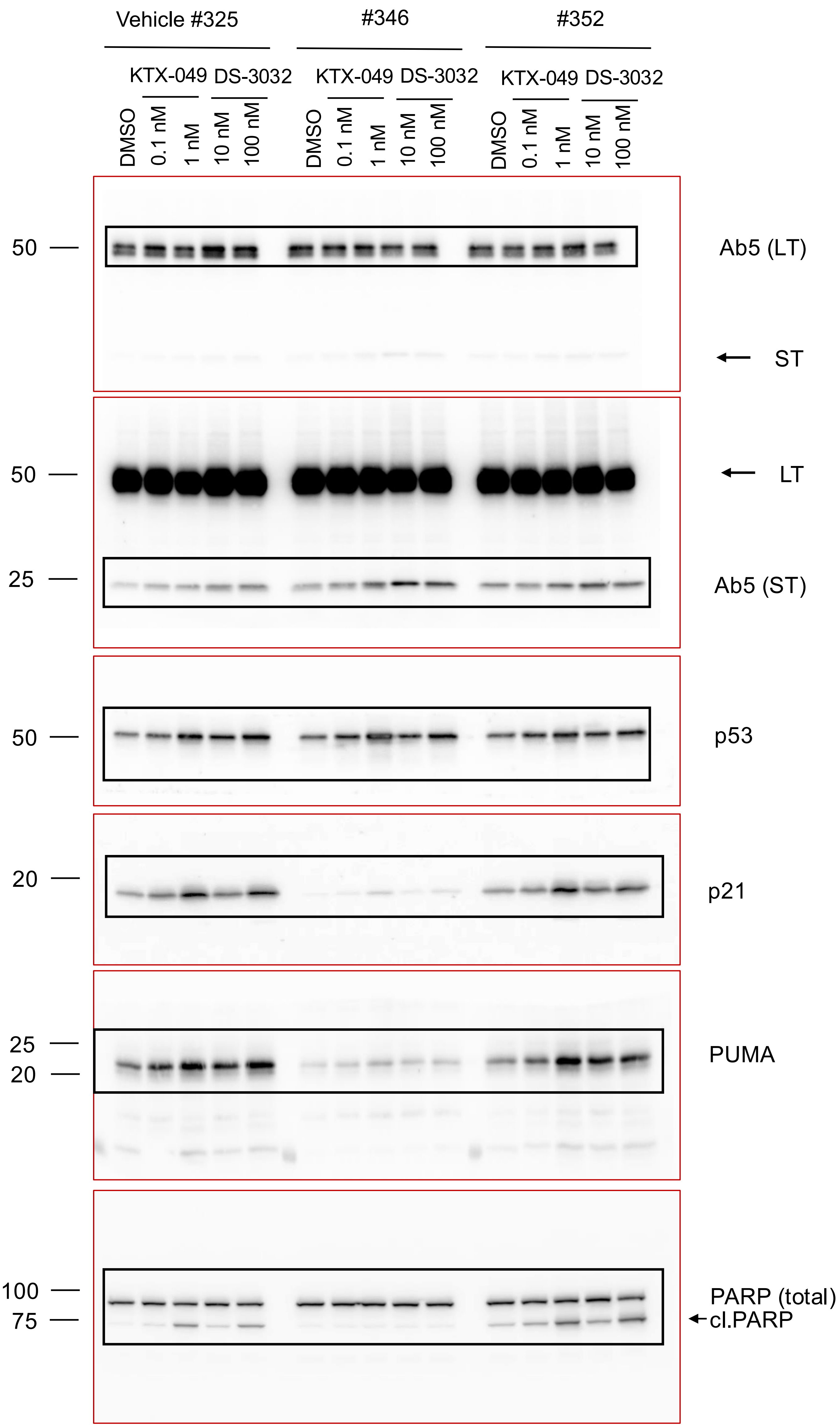

Supplemental Figure 17A Part II

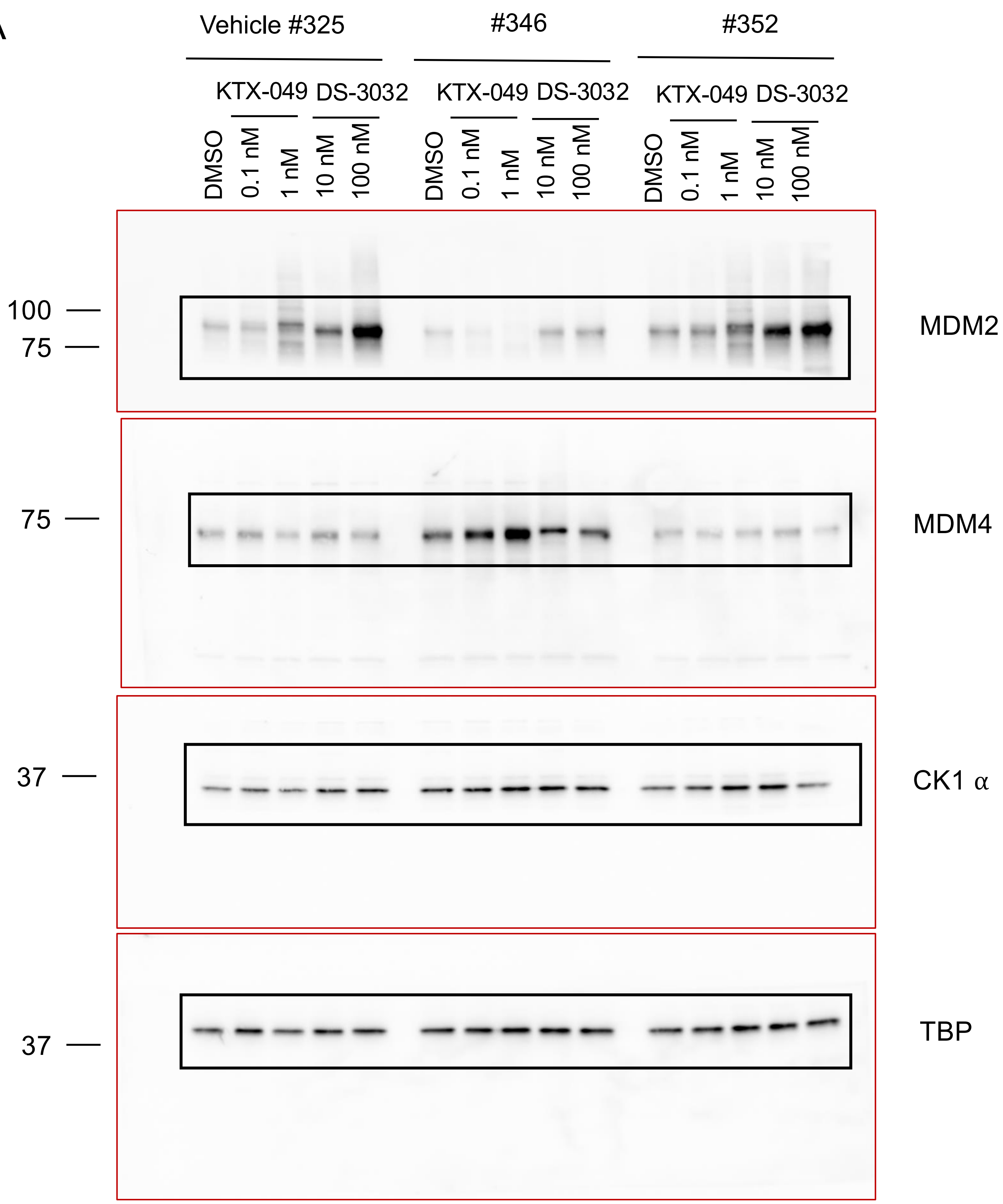

Supplemental Figure 17B Part I

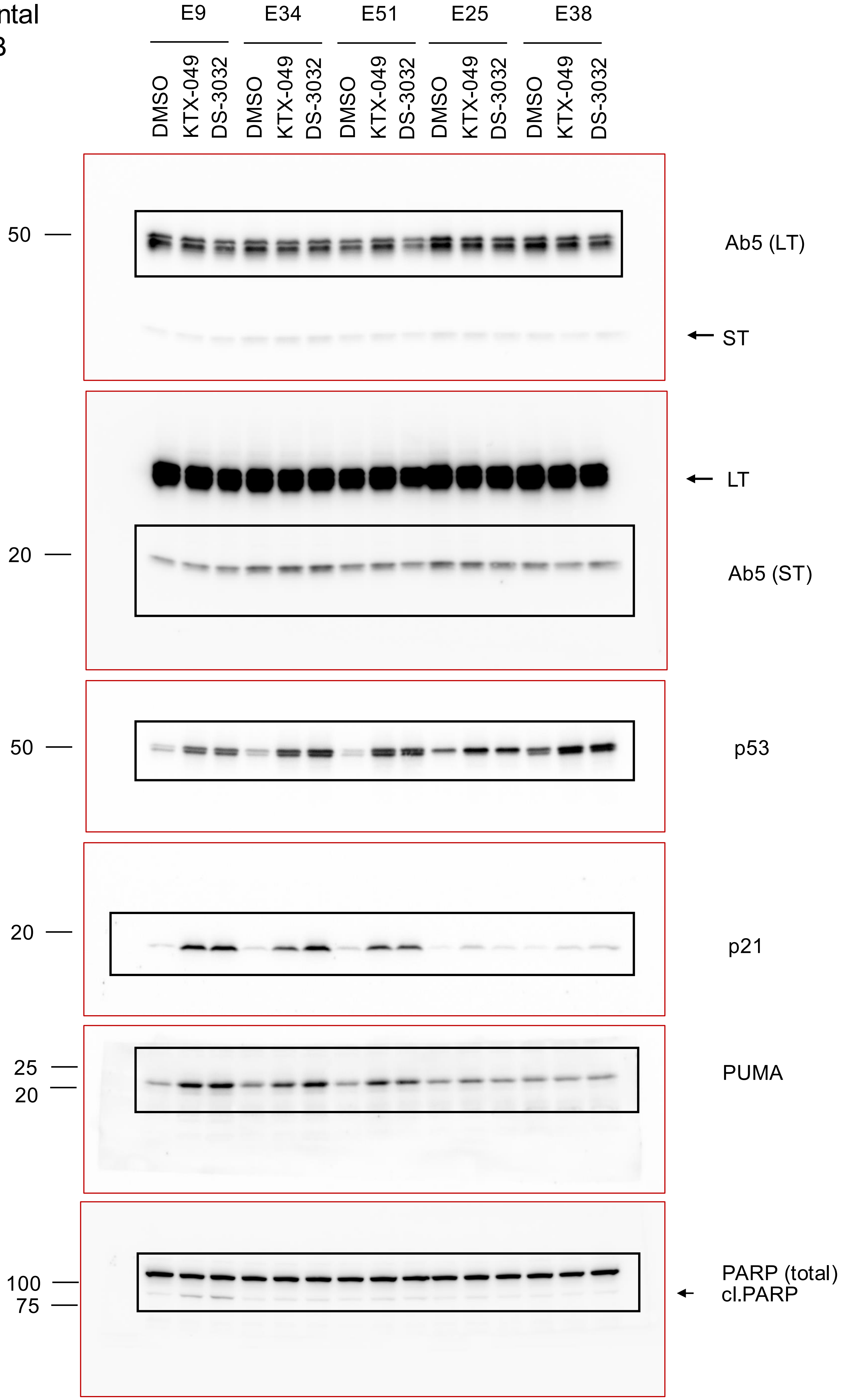

Supplemental Figure 17B Part II

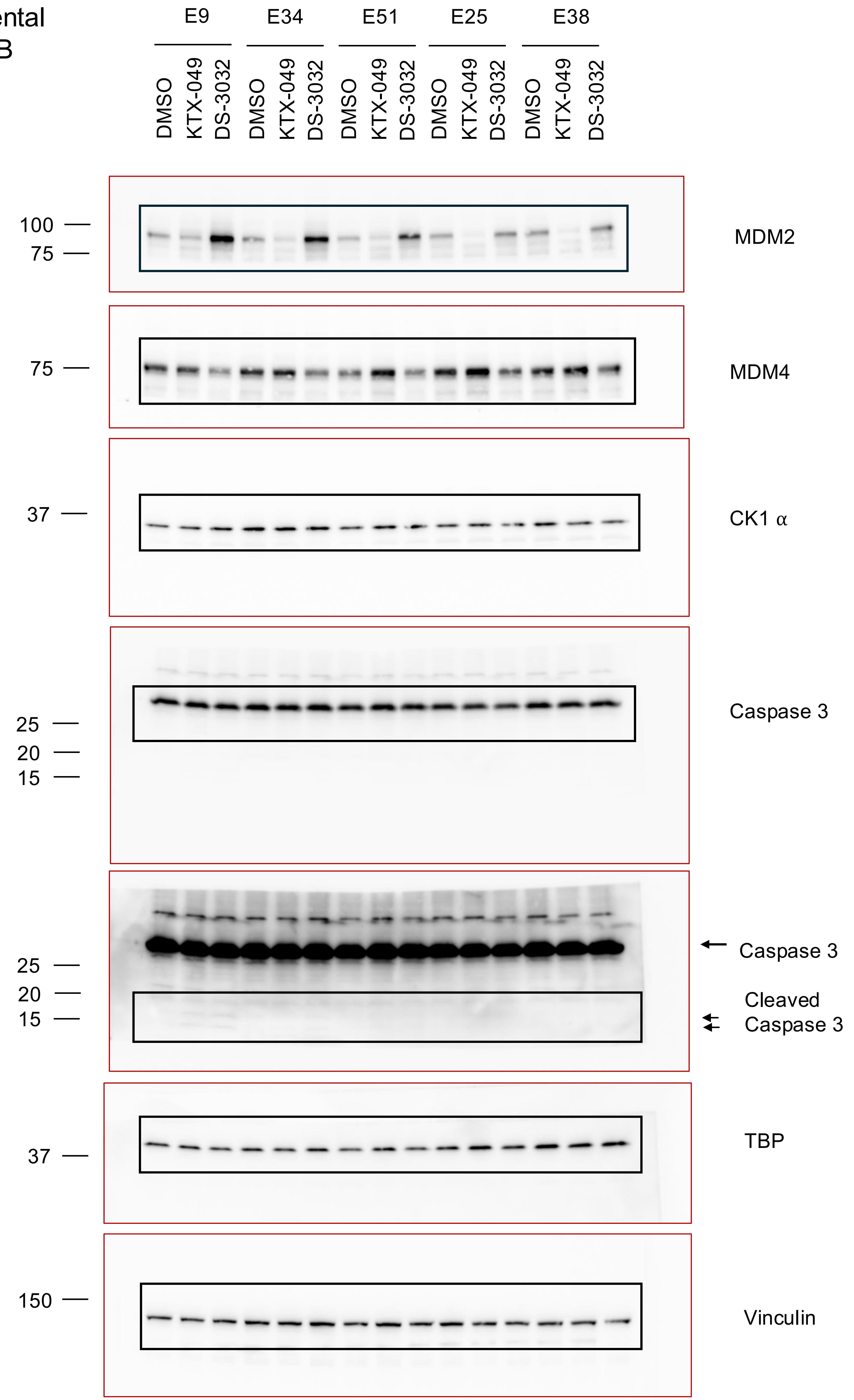

Supplemental
Figure 19B
Part I

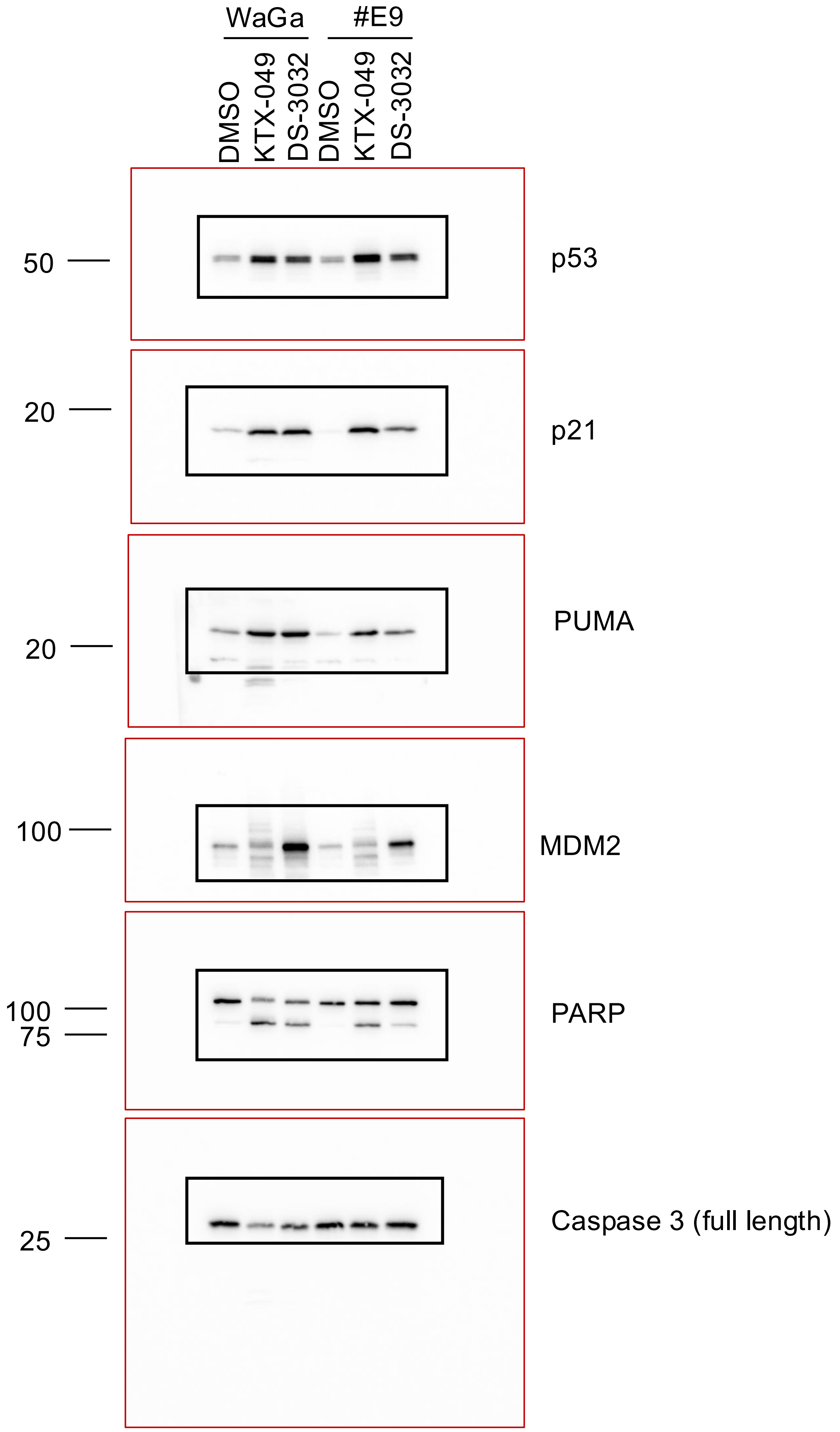

Supplemental
Figure 19B
Part II

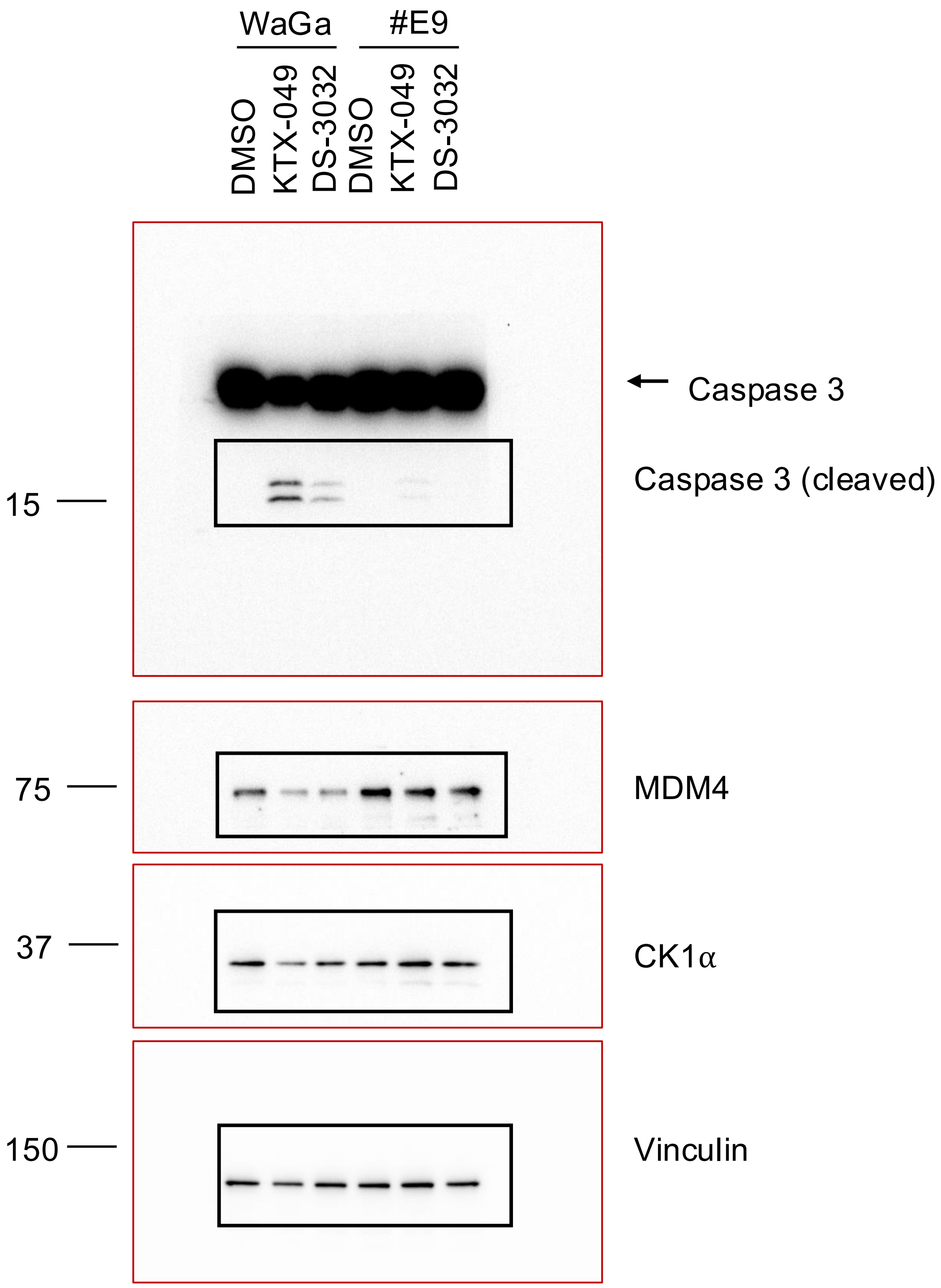

Supplement: Unedited blot and gel images [file jci-136-199049-s257.pdf]
